# Comprehensive genomic analysis of the *CNGC* gene family in *Brassica oleracea*: novel insights into synteny, structures, and transcript profiles

**DOI:** 10.1186/s12864-017-4244-y

**Published:** 2017-11-13

**Authors:** Kaleem U. Kakar, Zarqa Nawaz, Khadija Kakar, Essa Ali, Abdulwareth A. Almoneafy, Raqeeb Ullah, Xue-liang Ren, Qing-Yao Shu

**Affiliations:** 1 State Key Laboratory of Rice Biology, Institute of Crop Science, Zhejiang University, Hangzhou, 310058 China; 2 Molecular Genetics Key Laboratory of China Tobacco, Guizhou Academy of Tobacco Science, Guiyang, 550081 China; 3 Wuxi Hupper Bioseed Technology Academy Ltd., Wuxi, 214000 China; 4 Department of Biotechnology, BUITEMS, Quetta, Pakistan; 5 Department of Biological sciences, College of Education and Science, Albaydaa University, Rada’a, Yemen; 6 Department of Environmental Sciences, Quaid –i- Azam University, Islamabad, Pakistan; 7 Institute of Crop Sciences, Zhejiang University, 866 Yuhangtang Road, Hangzhou, 310029 China; 8 Guizhou Academy of Tobacco Science, Longtanba Road No. 29, Guanshanhu District, Guiyang, (550081) Guizhou People’s Republic of China

**Keywords:** Abiotic and biotic stress, Ion channels, CNGC, Expression pattern, *Brassica oleracea*, Evolution, RNA-seq, qRT-PCR analysis

## Abstract

**Background:**

The cyclic nucleotide-gated ion channel (CNGC) family affects the uptake of cations, growth, pathogen defence, and thermotolerance in plants. However, the systematic identification, origin and function of this gene family has not been performed in *Brassica oleracea*, an important vegetable crop and genomic model organism.

**Results:**

In present study, we identified 26 *CNGC* genes in *B. oleracea* genome, which are non-randomly localized on eight chromosomes, and classified into four major (I-IV) and two sub-groups (i.e., IV-a and IV-b). The *BoCNGC* family is asymmetrically fractioned into the following three sub-genomes: least fractionated (14 genes), most fractionated-I (10), and most fractionated-II (2). The syntenic map of *BoCNGC* genes exhibited strong relationships with the model *Arabidopsis thaliana* and *B. rapa CNGC* genes and provided markers for defining the regions of conserved synteny among the three genomes. Both whole-genome triplication along with segmental and tandem duplications contributed to the expansion of this gene family. We predicted the characteristics of BoCNGCs regarding exon-intron organisations, motif compositions and post-translational modifications, which diversified their structures and functions. Using orthologous *Arabidopsis* CNGCs as a reference, we found that most CNGCs were associated with various protein–protein interaction networks involving CNGCs and other signalling and stress related proteins. We revealed that five microRNAs (i.e., bol-miR5021, bol-miR838d, bol-miR414b, bol-miR4234, and bol-miR_new2) have target sites in nine *BoCNGC* genes. The *BoCNGC* genes were differentially expressed in seven *B. oleracea* tissues including leaf, stem, callus, silique, bud, root and flower. The transcript abundance levels quantified by qRT-PCR assays revealed that *BoCNGC* genes from phylogenetic Groups I and IV were particularly sensitive to cold stress and infections with bacterial pathogen *Xanthomonas campestris* pv. *campestris*, suggesting their importance in abiotic and biotic stress responses.

**Conclusion:**

Our comprehensive genome-wide analysis represents a rich data resource for studying new plant gene families. Our data may also be useful for breeding new *B. oleracea* cultivars with improved productivity, quality, and stress resistance.

**Electronic supplementary material:**

The online version of this article (10.1186/s12864-017-4244-y) contains supplementary material, which is available to authorized users.

## Background

Calcium is a universal secondary messenger that participates in multiple eukaryotic signalling pathways [1]. In plants, Ca^2+^ signal transduction via calcium-conducting channels is an important mechanism for transducing the signals derived from diverse environmental and developmental stimuli [2, 3]. Additionally, signal transductions contribute to growth, plant biotic interactions, and responses to hormones, light, and salt stress [4]. Cyclic nucleotide-gated ion channels (CNGCs) are components of Ca^2+^-conducting signal transduction pathways [5]. They are Ca^2+^-permeable cation-conducting channels that transport sodium, calcium, and potassium cations across membranes. Localized in the plasma membrane [6, 7], vacuole membrane [8], or nuclear envelope [9], CNGCs are controlled from inside the cell by secondary messengers such as Ca^2+^/calmodulin (CaM) and cyclic nucleotide monophosphates (cNMPs; 3′,5′-cAMP and 3′,5′-cGMP) [3, 6, 10, 11]. The CNGCs are hypothesized to be involved in the uptake of both essential and toxic cations, Ca^2+^ signalling, development, pollen fertility and tip growth, gravitropism, leaf senescence, innate immunity, pathogen defence, and abiotic stress tolerance [6, 12–15].

The application of bioinformatics tools (for genes/proteins prediction and phylogenetic analysis), and experimental approaches (gene expression, mutant analysis and overexpression in yeast/Escherichia coli) have led to the identification, characterization, and functional analysis (in exceptional cases) of *CNGC* family genes in important plant species, including *Arabidopsis thaliana* [5], rice [16], tomato [17], pear [18], and *Physcomitrella patens* [19]. Researchers have only recently started to investigate the evolution, function (and underlying regulatory mechanism) of plant CNGCs, as well as their phylogenetic relationships with other channels. Briefly, plant CNGCs are characterised by conserved structural components, including a short cytosolic N-terminus, six transmembrane helices (S1–S6) with a pore-forming region between S5 and S6, and a cytosolic C-terminus containing a cNMP-binding domain (CNBD). The CNBD is the most conserved region of CNGCs carrying a plant CNGC-specific motif spanning the phosphate-binding cassette (PBC) and hinge region, which mediates channel gating by cAMP and/or cGMP [3, 20]. A latest study of the *A. thaliana CNGC12* gene suggested that plant CNGCs have multiple CaM-binding domains (CaMBDs) at cytosolic N- and C-termini [3]. Moreover, channel functionality depends on CaM binding to the conserved isoleucine–glutamine (IQ) motif in the C-terminus of the channel, indicating CaM positively and negatively regulates CNGCs [3]. Studies on individual isoforms and the *A. thaliana CNGC* family revealed that plant *CNGC* genes may be functionally distinguished in a group-dependent manner. For example, *AtCNGC19* and *AtCNGC20*, which belong to Group IV-a, are involved in salt stress responses [8]. Additionally, *AtCNGC2* and *AtCNGC4*, which are Group IV-b members, affect disease resistance against various pathogens and thermotolerance [21, 22]. Mumtaz et al. [4, 17] recently concluded that Group IV-b *SlCNGC* genes regulate different types of resistance against diverse pathogens in tomato. It is unclear whether this also applies to other plant species.

*Brassica oleracea* (2n = 18) is a member of the family *Brassicaceae* (approximately 338 genera and 3709 species), which consists of many important vegetable and oilseed crops, including brussels sprout, kohlrabi, and kale [23]. Among the cultivated species, *B. oleracea* exhibits the largest genetic and morphological diversity, making it highly adaptable to different environments. Sexually compatible *B. oleracea* crops, such as cabbage, cauliflower, and broccoli, are valued for their economic, nutritional, and potent anticancer properties [24]. The whole-genome sequence of this plant species was recently published [24], which enabled us to study the *B. oleracea CNGC* family. We used in silico and experimental approaches to identify, characterise, and functionally verify *CNGC* gene family members. We applied multiple tools and programs to complete in-depth analyses of each *CNGC* gene family member, including an analysis of the physiological and biochemical properties of the encoded proteins. Our objective was to elucidate the diversification, expansion, and evolution of the *CNGC* gene family. Furthermore, we investigated *CNGC* expression patterns to clarify the mechanisms underlying their responses to biotic and abiotic stresses, and to identify novel genes potentially useful for breeding.

## Results

### Genome-wide identification of *CNGC* genes in *Brassica oleracea*

For a complete overview of the *B. oleracea CNGC* gene family, we used the 20 *A. thaliana CNGC* genes as queries in BLAST searches of the Ensembl Plants database. Out of the 34 non-redundant putative gene sequences retrieved, eight gene accessions with truncated sequences or lacking *CNGC*-specific domains (CNBD and transmembrane) were eliminated from analyses (Additional file 1). Finally, 26 *CNGC* genes containing both essential domains (PF00520/PF07885 and PF00027) and a CNGC-specific motif were identified in the *B. oleracea* genome (i.e., *BoCNGC1–26*). Of the 26 *BoCNGC* genes identified in the latest genome assembly version in Ensembl Plants, 16 and 24 were detected in earlier versions from Bolbase (v.1.3) and GenBank (v.2.1) respectively (Table 1).

**Table 1:** Properties of 29 *BoCNGC* genes identified in *Brassica oleracea*

Name	Genes ID (Ensemble v.2.1)	brassicadb (v.1.3)	GeneBank	Transcript (bp)	Protein length (aa)	Mol.Wt. (kDa)	pI	GRAVY	Aliphatic index	II	Total atoms	Ave. residue Wt. (g/mol)	Net charge
BoCNGC1	Bo3g054400	Bol010786	LOC106335861	2115	704	81.33	9.18	−0.212	90.88	47.95	11,473	115.5	22
BoCNGC2	Bo4g009240	Bol000930	LOC106342665	2112	703	81.09	9.78	−0.139	87.92	37.21	11,462	115.3	33.5
BoCNGC3	Bo4g158880	Bol026503	LOC106336663	1950	649	75.08	9.89	−0.023	99.14	39.96	10,654	115.7	39
BoCNGC4	Bo8g100190	Bol045794	LOC106312466	2214	737	84.58	9.74	−0.177	92.51	49.74	11,989	114.8	31
BoCNGC5	Bo4g154120	Bol041958	LOC106342509	2238	745	85.27	9.85	−0.193	89.69	50.69	12,073	114.5	36.5
BoCNGC6	Bo7g114580	Bol033673	LOC106306102	2226	741	84.49	9.53	−0.169	91.61	48.38	11,959	114.0	25.5
BoCNGC7	Bo2g028960	Bol015575	LOC106326694	2166	721	82.52	9.35	−0.122	89.28	50.45	11,653	114.4	26
BoCNGC8	Bo5g021460	Bol038188	LOC106294829	2226	741	85.08	9.17	−0.266	86.22	51.02	11,997	114.8	19.5
BoCNGC9	Bo4g071760	Bol013404	LOC106337043	2055	684	79.43	10.08	−0.11	91.23	49.17	11,253	116.1	38
BoCNGC10	Bo1g013250	Bol001429	LOC106308171	2184	727	84.17	8.8	−0.187	91.21	45.73	11,876	115.8	17
BoCNGC11	Bo4g154790	Bol041907	LOC106340005	2187	728	83.84	9.12	−0.096	93.48	46.34	11,863	115.2	21.5
BoCNGC12	Bo8g099660	Bol044680	LOC106309975	2202	733	84.52	9.29	−0.13	94.69	48.3	11,982	115.3	23
BoCNGC13	Bo9g165600	Bol030411	LOC106316400	2106	701	79.63	8.23	−0.149	84.86	45.08	11,147	113.6	12
BoCNGC14	Bo8g076590	Bol008733	LOC106311631	2121	706	81.58	8.36	−0.247	86.08	52.08	11,403	115.6	15
BoCNGC15	Bo2g042020	Bol037445	LOC106327817	2088	695	80.24	8.25	−0.176	91.12	55.15	11,285	115.5	13.5
BoCNGC16	Bo9g116160	Bol038844	LOC106319027	2097	698	80.78	8.19	−0.192	90.6	55.83	11,357	115.7	12
BoCNGC17	Bo9g164130		LOC106317764	2151	716	81.93	9.81	0.018	94.61	54.04	11,569	114.4	38.5
BoCNGC18	Bo3g070140		LOC106335359	2160	674	76.69	8.38	−0.121	89.96	50.37	10,797	114.0	13.5
BoCNGC19	Bo3g070160			1677	558	63.94	7.26	−0.187	87.63	45.22	8953	114.6	5
BoCNGC20	Bo5g122720		LOC106343117	2262	753	85.98	9.8	−0.075	92.19	52.29	12,121	114.2	32
BoCNGC21	Bo1g119310		LOC106344554	2280	759	86.21	9.81	−0.067	90.59	47.96	12,147	113.6	32.5
BoCNGC22	Bo1g119340			2205	734	82.99	9.2	0.033	93.37	47.16	11,692	113.1	28
BoCNGC23	Bo1g079060		LOC106299574	2370	789	89.00	10.18	−0.055	95.64	46.62	12,604	112.8	42
BoCNGC24	Bo1g119320		LOC106326537	2232	743	85.06	10.06	−0.103	90.93	53.01	11,990	114.5	37
BoCNGC25	Bo5g122750		LOC106293228	2346	781	89.78	10.11	−0.176	90.73	47.88	12,686	115.0	34.5
BoCNGC26	Bo5g122740		LOC106293974	2235	744	85.72	9.3	−0.231	85.44	53.09	12,036	115.2	21.5

The physiological and biochemical properties of the 26 BoCNGC proteins were determined by computing different parameters, and are tabulated in Table 1. These proteins varied in length from 558 to 789 amino acids, with an average of 717 amino acids. The ProtParam tool revealed that there was a considerable range in BoCNGC residue weight (112.795–116.128 g/mol) and molecular weight (63.938–89.775 kDa) depending on the number of atoms present. The computed average *pI* of majority of BoCNGC proteins was relatively high (range 8.23 to 10.18), signifying that these proteins are localized to membranes, and will supposedly participate in basic buffers. The BoCNGC19, which had *pI* than 7.4, indicate that this protein likely participate in the acidic buffers. Approximately one third of BoCNGC proteins had a low net charge (<17), while other are composed of more charged amino acids. Nearly all BoCNGC were hydrophilic, with BoCNGC17 and BoCNGC22 being slightly hydrophobic, which endorses its multifaceted role in cellular membrane transport. According to the instability index (II), only two proteins were stable in test tubes, namely BoCNGC2 and BoCNGC3. Aliphatic index showed that most BoCNGC proteins were thermostable at a wide temperature ranges, similar to other globular proteins.

### Phylogenetic analysis of *BoCNGC* genes

**Fig. 1:**
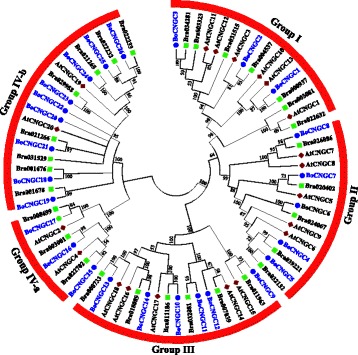
Phylogenetic tree of *Brassica oleracea*, *Arabidopsis thaliana,* and *Brassica rapa* CNGC proteins. A maximum likelihood phylogenetic tree was created with MEGA 6.0, using the Jones–Taylor–Thornton model. The bootstrap values from 1000 replications are provided at each node. The BoCNGC proteins identified in this study are indicated with blue circles, while the AtCNGCs and BrCNGCs are indicated with maroon diamonds and green rectangles, respectively

Multiple sequence alignments and a maximum likelihood phylogenetic tree constructed between BoCNGCs and AtCNGCs were used to determine the similarity and homology between the *B. oleracea* and *A. thaliana CNGC* families. To strengthen the phylogenetic analysis, we identified and included 29 *CNGC* homolog genes from sister specie *Brassica rapa* (*BrCNGCs*) in current analysis. The sequence alignment revealed high similarity between the amino acid sequences of the three species, especially at the conserved domain regions (Additional file 2). The topology of the inferred maximum likelihood scoring tree revealed that the *BoCNGC* gene family can be divided into four major groups (i.e., Groups I–IV), which are based on the *A. thaliana* groups (Fig. 1) [5]. Groups I–III are monophyletic, while Group IV is sub-divided into two distinct clades (i.e., Groups IV-a and IV-b). Group IV contains 12 *BoCNGC* genes, while the other groups contain three to six members. Moreover, individual phylogenetic trees that were constructed based on the aligned *B. oleracea* and *A. thaliana* CNGC proteins produced similar clustering patterns (Additional files 3 and 4).

### Chromosomal distribution and diversification of *BoCNGC* genes

**Fig. 2:**
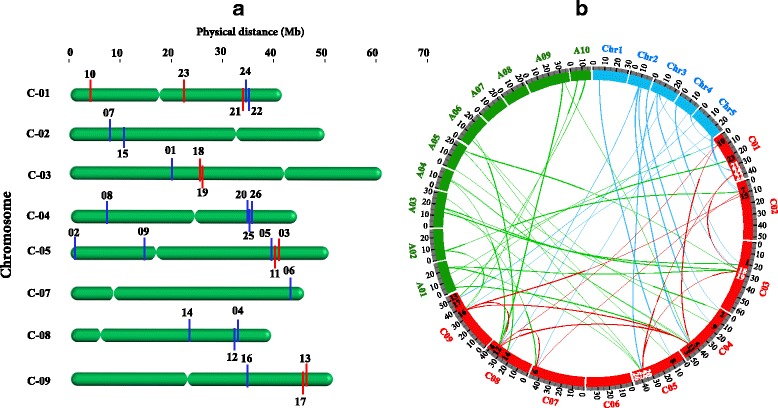
Chromosomal localization, synteny, and expansion of the *B. oleracea CNGC* gene family. **a** Physical locations and distances of the *BoCNGC* genes across the eight *Brassica oleracea* chromosomes. Red and blue lines correspond to forward and reverse orientations of each locus, respectively. **b** Circos plot presenting gene duplication (tandem and segmental) events and synteny of the *BoCNGC* genes. The *BoCNGC* genes are presented as numbers on the *B. oleracea* chromosomes (red). Tandem and segmental duplications are indicated by white numbers and red lines, respectively. Syntenic relationships with 10 *Brassica rapa* (A01 to A10) and five *Arabidopsis thaliana* (Chr1 to Chr5) chromosomes are represented as green and blue lines, respectively. The maps are based on orthologous pair positions, and reveal highly conserved syntenic relationships

The 26 *BoCNGC* genes were mapped onto *B. oleracea* chromosomes, and the position of each locus was determined. These genes were randomly distributed across the genome, and were detected on eight of nine chromosomes (i.e., C1–5 and C7–C9). The *BoCNGC* genes were unevenly distributed, with some chromosomes (i.e., C1 and C5) carrying five genes, while the rest had fewer genes (e.g. C7). Chromosome 6 did not carry any of the *BoCNGC* genes (Fig. 2a).

### Gene duplication events

Gene family expansion occurs via the following three mechanisms: tandem duplication, segmental duplication, and whole-genome duplication [25]. We investigated gene duplication events to clarify the genome expansion mechanism of the *B. oleracea BoCNGC* superfamily. An evaluation of the physical distance between *BoCNGC* gene loci revealed that eight genes (i.e., *BoCNGC18*/*BoCNGC19*, *BoCNGC21/BoCNGC22/BoCNGC24*, and *BoCNGC20/BoCNGC25/BoCNGC26*) were tandemly duplicated. These genes were detected on C3, C1, and C5, respectively. The data obtained from the Plant Genome Duplication Database revealed that 13 *BoCNGC* genes distributed across the *B. oleracea* genome were associated with segmental duplications (Fig. 2b). The *BoCNGC* gene clusters likely formed via tandem and segmental duplication events may have expanded and enhanced the functional diversity of the gene family.

### Comparative syntenic and evolutionary analyses of orthologous *CNGC* gene pairs

The *B. oleracea* and *B. rapa* genomes are currently divided into three sub-genomes, namely LF (least fractionated), MF-I (most fractionated), and MF-II [26]. We observed that the *B. oleracea* LF sub-genome contains the most *BoCNGC* genes (14), followed by sub-genomes MF-I (10) and MF-II (2) (Additional file 5). Because of a *Brassica*-lineage specific whole-genome triplication (WGT) [27], each *A. thaliana CNGC* gene was expected to generate three *Brassica* copies. However, there were 20 *A. thaliana CNGC* genes, 26 *B. oleracea CNGC* genes, and 29 *B. rapa CNGC* genes. To detect the retention or loss of *CNGC* genes after a WGT event, the syntenic map of *BoCNGC* genes with the model *A. thaliana* and *B. rapa CNGC* genes provided markers for defining the regions of conserved synteny among the three genomes (Fig. 2b). Compared with the ancestral *Brassicaceae* blocks (A to X) in *A. thaliana*, the synteny of 15 *AtCNGC* genes was preserved in *Brassica* species, based on the number of corresponding genes. Ten of the 20 *AtCNGC* genes were retained as a single copy in the equivalent blocks of both *Brassica* species. Three *AtCNGC* genes (i.e., AT2G23980, AT2G24610, and AT5G54250) located on the I and W syntenic blocks, were preserved as two copies in *Brassica* genomes, which were asymmetrically fractionated into three sub-genomes. Two *AtCNGC* genes (i.e., AT3G17690 and AT3G17700) in the F syntenic block were retained as three copies in each species. Two extra gene copies (i.e., *BoCNGC20* and *BoCNGC22*) were located on potential overlap/tandem repeat regions of the *B. oleracea* genome, thus producing phylogenetic cluster IV-b. Approximately 25 *B. oleracea CNGC* genes and 24 *B. rapa CNGC* genes exhibited clear syntenic relationships among the three species. Two gene pairs (i.e., *BoCNGC3* and *BoCNGC23*; Bra034281 and Bra029958) were not part of an *A. thaliana* syntenic block (Additional file 6), suggesting that these genes originated after the divergence from *A. thaliana*. The remaining four *B. rapa* genes were likely generated after the speciation event. In addition, 11 *BoCNGC* genes exhibited strong syntenic relationships with the genes from other plant species, implying this gene family is important for plant growth, development, and stress resistance (Additional file 6).

The orthologous *CNGC* gene pairs between the *B. oleracea* and *A. thaliana* genomes were used to estimate the Ka, Ks, and Ka/Ks values (Table 2). The mean Ka/Ks value of all orthologous gene pairs in the *B. oleracea CNGC* gene family was 1.98. Most of the *BoCNGC* genes had Ka/Ks ratios greater than 1. Additionally, the minimum and maximum Ka/Ks ratios were 1.05 (*BoCNGC26*) and 7.7 (*BoCNGC6*), respectively. These findings indicate that the *BoCNGC* gene family is under positive selection pressure, and might preferentially conserve functions and structures under this selective pressure.

**Table 2:** Comparative analysis of Ka, Ks and Ka/Ks values for *CNGC* gene pairs between *B. oleracea* compared to *A. thaliana*. Ka/Ks ratio greater than 1 indicates positive selection, a ratio less than 1 indicates functional constraint, and a Ka/Ks ratio equal to 1 indicates neutral selection

*A. thaliana* genes	*B. oleracea* genes	KA	KS	KA/KS
AtCNGC13	BoCNGC1	0.137	0.032	4.303387233
AtCNGC3	BoCNGC2	0.147	0.029	5.117236906
AtCNGC6	BoCNGC4	0.136	0.052	2.615208996
	BoCNGC5	0.167	0.048	3.468082016
AtCNGC9	BoCNGC6	0.188	0.024	7.768521972
AtCNGC5	BoCNGC7	0.124	0.040	3.098687155
AtCNGC7	BoCNGC8	0.111	0.033	3.40004813
AtCNGC15	BoCNGC9	0.147	0.060	2.456018066
AtCNGC17	BoCNGC10	0.113	0.034	3.357834045
AtCNGC14	BoCNGC11	0.133	0.031	4.245278743
	BoCNGC12	0.145	0.040	3.651430365
AtCNGC18	BoCNGC13	0.094	0.048	1.949453718
AtCNGC16	BoCNGC14	0.111	0.066	1.676233706
AtCNGC4	BoCNGC15	0.101	0.025	4.039237878
	BoCNGC16	0.103	0.034	3.056103924
AtCNGC2	BoCNGC17	0.118	0.029	4.091069466
AtCNGC19	BoCNGC18	0.246	0.126	1.950211367
AtCNGC20	BoCNGC19	0.202	0.178	1.133449904
	BoCNGC20	0.136	0.049	2.79662626
	BoCNGC21	0.099	0.041	2.44174101
AtCNGC19	BoCNGC22	0.202	0.119931313	1.680530313
	BoCNGC24	0.146	0.081	1.792823624
	BoCNGC25	0.146	0.081	1.792823624
AtCNGC20	BoCNGC26	0.131	0.125	1.054276842

### Domain architecture and alignment of BoCNGC proteins

**Fig. 3:**
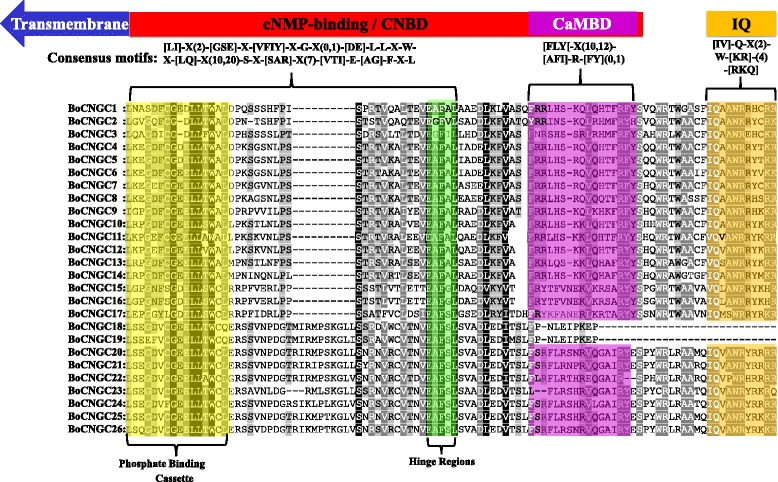
Multiple sequence alignment of BoCNGC-specific domains, and a three-dimensional model of BoCNGC1. Cartoon model with characteristic CNGC domains provided on top. The BoCNGC-specific consensus motif keys are listed below the cartoon. Amino acids allowed in a specific position are presented in square brackets. X represents any amino acid, while numbers in round brackets indicate the number of amino acids. The multiple sequence alignment of BoCNGC proteins is presented with the CNBD, CaMBD, and IQ domain indicated with different colours. The CNBD domain includes a conserved PBC and hinge region, followed by the CaMBD. Residues shaded in black and grey indicate 100% and >50% similarity among the 26 BoCNGCs

Domain composition analyses revealed that BoCNGC proteins contain two primary domains, namely CNBD and TM (Additional file 7). The sequence alignment of 26 BoCNGCs indicated that the two most conserved regions within the CNBD domain are a PBC, and an adjacent hinge region (Fig. 3; Additional file 8). The following highly conserved consensus motif was identified: [LI]-X(2)-[GSE]-X-[VFIY]-X-G-X(0,1)-[DE]-L-L-X-W-X-[LQ]-X(10,20)-S-X-[SAR]-X(7)-[VTI]-E-[AG]-F-X-L. This sequence can be used to classify newly annotated or un-annotated candidate sequences as *Brassica* CNGCs. Additionally, there was a relatively conserved IQ domain and a less conserved CaMBD adjacent to a CNBD present in 24 of the 26 BoCNGC proteins. Two proteins (i.e., BoCNGC18 and BoCNGC19) were observed to lack the CaMBD and IQ domains because their sequences are truncated at the C-terminal end of the CNBD. A high sequence divergence was noted among different groups, particularly between members of Sub-groups IV-a and IV-b. For example, the CaMBD [FLY[−X(10,12)-[AFI]-R-[FY](0,1), was not particularly conserved between Group IV-b and the other groups. However, the IQ motif [IV]-Q-X-X-W-R-X-X-X-[RKQ] was relatively conserved among the BoCNGC proteins (Fig. 3). Alignments between BoCNGCs, AtCNGCs, and BrCNGCs revealed a high sequence divergence at the C-terminal of the CNBD, in which several Group IV-b members lack the CaMBD and IQ motif (Additional files 9 and 10). Overall, our in silico analyses suggest that ion transport and CNBDs along with the PBC and hinge region are conserved in all three species, and are characteristic of plant CNGCs.

### Gene structure and motif composition analysis

**Fig. 4:**
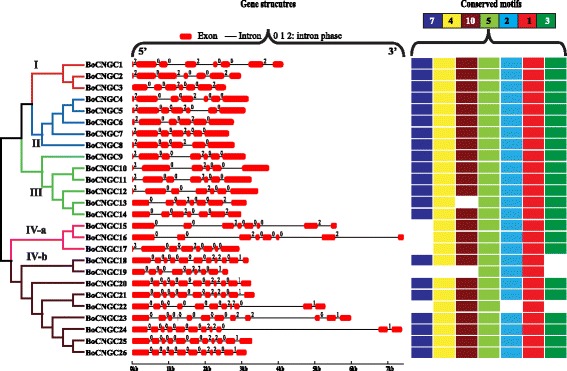
Schematic diagram presenting the *BoCNGC* gene structures and the conserved motifs in the encoded proteins. The neighbour-joining phylogenetic tree is provided on the left side of the figure, followed by the exons–introns, which are indicated as red boxes and black lines, respectively. Motifs are represented by numbers in coloured boxes on the right. Numbers [0, 1 and 2] provided on the gene structures represent the respective intron phases. The length of each exon and intron can be determined using the provided scale. The order of the motifs corresponds to the motif positions in the protein sequence. However, the length of the box does not correspond to the length of the motif

To characterise the structural diversity of the *BoCNGC* family members, we analysed the exon–intron organization of individual *BoCNGC* genes. The majority of the *BoCNGC* genes from phylogenetic Groups I–III contained six or seven exons, while the Group IV members had 8–11 exons (Fig. 4). Closely clustered *BoCNGC* genes in the same clades were similar regarding the number of exons and intron lengths. Most of the introns in *BoCNGC* genes were phase 0 introns, which occur in between complete codons. Fifty-four phase 2 introns (i.e., located between the second and third nucleotides of a codon) were observed in the *BoCNGC* family, in which the genes carried two phase 2 introns. The exceptions were *BoCNGC1* and *BoCNGC2*, which contained three phase 2 introns. Only the members of phylogenetic Group IV-b had single phase 1 introns at the terminal end of their sequences. A comparison between the exon–intron organizations of *BoCNGC* genes and the *AtCNGC* genes clustered in the same phylogenetic groups revealed several differences (Additional file 11). Most of the phase 1 introns were present in *AtCNGC* genes, implying that intron loss during evolution resulted in a decrease in the number of introns in *BoCNGC* genes, particularly those in Groups I–III and IV-a.

The BoCNGC protein sequences were used for domain or motif structure analyses with the Multiple Expectation Maximization for Motif Elicitation suite [28]. Ten conserved motifs were identified. According to Pfam codes [29] and WebLogo, only seven motifs (i.e., 1–5, 7, and 10) encode domains with known functions (Fig. 4; Additional files 12 and 13). Motif 2 was the biggest motif encoding a conserved domain, which is probably associated with peptidase_C50, putative aminopeptidase, or DNA polymerase III subunit tau_4. Motifs 1 and 5, which encode a CNBD and an ion transport domain, respectively, were conserved among all BoCNGC family members. The ion transport domain had the most motifs, including motifs 4, 5, 7, and 10. The IQ CaM-binding motif (PF00612) was conserved among BoCNGC family members, with the exception of BoCNGC18, 19, and 22. Group IV proteins contained the fewest functionally annotated motifs, suggesting that the closely related proteins in each group have similar motifs and are also probably functionally similar. The functions of the remaining motifs (i.e., 6, 8, and 9) remain to be determined.

### Post-translational modification and phosphorylation of BoCNGC proteins

When BoCNGC protein sequences were analysed using ScanProsite [30], multiple putative phosphorylation sites were revealed. These sites may act as substrates for several kinases, including casein kinase II, protein kinase C, tyrosine kinase, and cAMP/cGMP kinases. Protein kinase C, a family of ten isoenzymes that play a vital role in cellular signal transduction [31], were the most abundant, with 16 sites in BoCNGC4, BoCNGC5, BoCNGC8, and BoCNGC12. Casein kinase II sites, which were the most abundant in Group IV members, are reported to influence different developmental and stress responsive pathways in Arabidopsis [32]. All BoCNGC proteins had multiple N-myristoylation/N-glycosylation motif sites, which are highly conserved compared with the other PTMs. The lipid modification by N-myristoylation might controls the redox disproportions originating from different stresses in plants [33], while glycosylation is crucial for correct growth [34]. The BoCNGC5 and BoCNGC18 proteins contained the most N-myristoylation (11) and N-glycosylation (10) sites, respectively. Other PTM sites, such as those for amidations, tyrosine kinase, serine- and glutamic acid- rich regions, cell attachment sequences, and leucine zipper patterns, were less conserved and randomly distributed (Table 3). Such phosphorylations deliver effective means to regulate most physiological activities, including metabolism, transcription, DNA replication and repair, cell proliferation [35].

**Table 3:** The number of predicted post-translational modification sites in BoCNGC protein sequences

Protein ID	CAMP	CK2	AMD	PKC	ASN	TYR	MYR	RGD	LEU	SER	GLU	ATP
BoCNGC1		7	2	8	7	1	4					
BoCNGC2	2	6		7	4	1	7					
BoCNGC3	2	7		10	4	1	3					
BoCNGC4	4	3		16	5	1	8					
BoCNGC5	3	4		16	4	1	10					
BoCNGC6	2	8		14	6	1	11					
BoCNGC7	1	5	1	9	5	2	7		1			
BoCNGC8	3	6		16	3	1	7					1
BoCNGC9	1	4		12	4	2	8					
BoCNGC10	1	6		12	4	2	7					
BoCNGC11	1	7		15	2	1	5		3			
BoCNGC12	2	9		16	2	1	5					
BoCNGC13	2	8	1	11	7		9		3			
BoCNGC14	1	8		13	5	1	8					
BoCNGC15	1	12	1	8	4		9		1		1	
BoCNGC16	2	13		10	5		8				1	
BoCNGC17	2	9		6	3	1	6					
BoCNGC18	2	11	1	9	10	1	5		1			
BoCNGC19	1	10		9	7	1	3	1	1			
BoCNGC20		13	1	9	2	1	5					
BoCNGC21		12	1	8	2		7	1				
BoCNGC22		11		6	4		5			1		
BoCNGC23	2	11	1	14	3		7					
BoCNGC24		14		15	6		8					
BoCNGC25		13		13	5		6					
BoCNGC26	1	14		6	7		4					

*cAMP/cGMP* cAMP/cGMP-binding motif profile, *SER* serine-rich region profile, *GLU* glutamic acid-rich region profile, *CAMP* cAMP- and cGMP-dependent protein kinase phosphorylation site; *CK2* casein kinase II phosphorylation site, *AMD* amidation site, *PKC* protein kinase C phosphorylation site, *ASN* N-glycosylation site, *TYR* tyrosine kinase phosphorylation site, *MYR* N-myristoylation site, *RGD* cell attachment sequence, *LEU* leucine zipper pattern, *ATP* ATP/GTP-binding site motif A (P-loop). Numbers given in each cell refer the total count of PTM sites found in each protein

### Prediction of functional association network of BoCNGC proteins

**Fig. 5:**
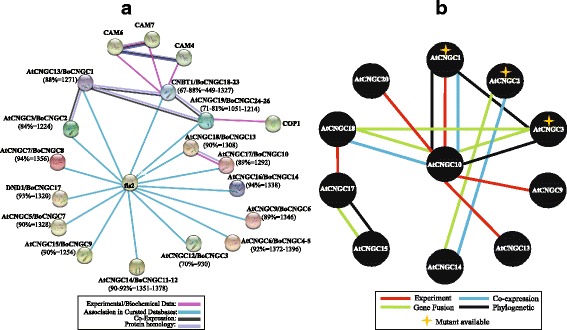
Hypothetical functional association network of CNGC proteins according to the orthologues relationship between *Arabidopsis thaliana* and *Brassica oleracea*. **a** STRING based protein-protein interaction network of CNGC proteins. Blast match results of string-network database showing similarity of *CNGC*-encoded proteins between of *B. oleracea* and *A. thaliana* are given in brackets (i.e., percentage and similarity index score). **b** AtPID (*Arabidopsis thaliana* Protein Interactome Database) based protein-protein interaction network of CNGC proteins. The details of each node, interaction type and reference are provided in Additional file 15

To explore the relationships among different BoCNGC proteins, a hypothetical protein–protein interaction network was in silico predicted with the STRING program (accessed in April 2016) [36] and AtPID (*Arabidopsis thaliana* Protein Interactome Database), using using orthologous AtCNGCs as query. The STRING interaction network for the first shell of interactors of AtCNGC proteins, supported by confidence score, is presented in Fig. 5a. Fourteen AtCNGCs, having 24 orthologs in *B. oleracea*, interact with flagellin-sensitive 2 (i.e., FLS2 or MPL12.8), represented by association in curated databases (confidence score: 0.8). This association was traced to manually curated plant–pathogen interaction pathway imported from the Kyoto Encyclopedia of Genes and Genomes database (Additional file 14). Supported by principal component analysis, a positive interaction (confidence score: 0.154) was observed between BoCNGC10 and BoCNGC13, which are the orthologues of AtCNGC17 and AtCNGC18, respectively. In another interaction network, BoCNGC1 interacts with BoCNGC2 and BoCNGC18–26, which are orthologues of AtCNGC13, 2, 19 and 20 respectively. This interaction is based on protein homology, association in curated human pathways (http://www.reactome.org/), or genes encoding these proteins have correlated expression levels. We also observed that the Group IV proteins are associated with constitutive photomorphogenic 1 and CaM proteins (i.e., CaM4, CaM6, and CaM7) (Fig. 5a).

Using orthologous *Arabidopsis* CNGCs as query in the AtPID uncover more potential interactions between CNGCs, and to other proteins, which are validated by experimental data from different assays (Fig. 5b; Additional file 15). The results exhibited strong interactions of co-expression and gene fusion between CNGC functional partners belonging to similar clades. For example, AtCNGC10 interacted with AtCNGC1, 3 and 13, while AtCNGC17 interacted with AtCNGC18 as mentioned earlier. AtCNGC10 interacted with more CNGCs than other proteins. In addition, some CNGCs (AtCNGC1, 5, 6, 9, 10, 13, 17, 18 and 19) interacted with many important signaling and stress related regulatory proteins, including calmodulins. These interactions are supported by data from yeast two-hybrid, and Affinity Capture-MS assays. Five *CNGC* genes (AtCNGC 1–4, and 11) were found to have available phenotypes of mutant data from seedlings, leaves and embryos, showing that these genes play important roles in hyper-sensitivity, pathogen and abiotic stress resistance (Additional file 15).

Additional evidence from experimental/biochemical data detected by protein kinase (MI:0424) and anti tag coimmunoprecipitation (MI:0007) assays in human putative homologs (i.e., Potassium voltage-gated channel 2 and Leucine rich repeat containing 47/Per-Arnt-Sim domain kinase) suggest a functional link between CNGCs and FLS2 [37, 38]. The experimental details and LC-MS/MS, yeast two-hybrid and phosphorylation of peptide arrays of human interacting KCNH2 and LRRC47/PASK proteins can be found in supplementary material of Behrends et al. [38]. Using Mating-Based Split Ubiquitin Assays in *A. thaliana*, Chen et al. [39] reported strong, positive (in both 500 μM methionine and at least one 150 μM methionine conditions), and statistically significant interaction between these protein pairs, which are required for polarized tip growth of pollen tube [40]. In another interaction network, BoCNGC1 interacts with BoCNGC2 and BoCNGC18–26, which are orthologues of AtCNGC13, 2, 19 and 20 respectively. Additionally, we observed a weak interaction (confidence score: 0.151) between AtCNGC13 (i.e., orthologues of BoCNGC1) and BRI-associated receptor kinase 1 (BAK1), which was previously observed between AtCNGC17 and BAK1 [41]. Though, it is reported that evidence transfer from one model organism to the other seems feasible approach to study interaction conservation, and it has been implemented in several frameworks already [42]. However, these experimental proofs are essential to support this analysis in *B. oleracea*.

### Identification of microRNA target sites

Identifying the targets of the predicted microRNAs (miRNAs) may provide insights into the biological functions of miRNAs influencing plant development, signal transduction, and stress adaptations [43]. We searched for potential miRNA targets in a set of identified *BoCNGC* transcripts using the plant small-RNA target analysis server (psRNATarget) [44]. Using a cut-off threshold of 5 for the search parameters, we identified 14 miRNAs with target sites in 17 *BoCNGC* transcripts, with expectation scores of 1.5–5 (Additional file 16). To decrease the number of false positive predictions, small-RNA/target site pairs with an expectation score and cut-off threshold of 3 were considered. Consequently, five miRNAs with target sites in nine *BoCNGC* genes were identified (Table 4). These miRNAs were localized to the 3′ arm of the stem-loop hairpin structure. Unlike bol-miR838d, which has five target genes, the remaining miRNAs have only one target gene. Moreover, only bol-miR838d has multiple target sites (i.e., complementary regions) on *BoCNGC15* and *BoCNGC16* transcripts. The accessibility of the target site varied from 2.883 (bol-miR838d) to 16.4 (bol-miR5021), where lower values correspond to a greater possibility of contact between the miRNA and target site. Four miRNAs were determined to be involved in cleaving the target transcript, while two miRNAs were predicted to inhibit the translation of target genes.

**Table 4:** Putative microRNA targets predicted in 26 *BoCNGC* transcripts

miRNA Acc.	Target Acc.	Expectation	Target Accessibility	Alignment	Inhibition	Multiplicity
bol-miR5021a/j	BoCNGC4	2.5	16.412	miRNA 20 AGAAGAAGAAGAAGAAGAAU 1 .::::::::::::::::: Target 60 CUUUCCUCUUCUUCUUCUUA 79	Cleavage	1
bol-miR838d	BoCNGC5	2.5	15.037	miRNA 20 CUUGUUCUUCUUCUUCUUCU 1 :::: .::::::::.::.:: Target 2884 GAAGAGGAAGAAGAGGAGGA 2903	Cleavage	1
bol-miR838d	BoCNGC6	2.0	15.121	miRNA 20 CUUGUUCUUCUUCUUCUUCU 1 :::::::.::.:::::::: Target 2593 GAAGAAGAGGAGGAAGAAGA 2612	Cleavage	1
bol-miR414b	BoCNGC8	3.0	16.071	miRNA 21 ACUUCUACUUCUACUUCUACU 1 :::::::::.::::::::: Target 2589 UGAAGAUGAGUAUGAUGAUGA 2609	Translation	1
bol-miR838d	BoCNGC10	2.0	8.16	miRNA 22 CACUUGUUCUUCUUCUUCUUCU 1 :::::::::::::::::::: Target 3520 GUGAUGAAGAAGAAGAAGAAGA 3541	Cleavage	1
bol-miR4234	BoCNGC12	3.0	15.182	miRNA 22 UGACGGUUGAUCAAAAUUCAAC 1 :.:.:::.:.:::::.::::: Target 2630 AUUUUCAAUUGGUUUUGAGUUG 2651	Cleavage	1
bol-miR838d	BoCNGC15	1.5	6.045	miRNA 20 CUUGUUCUUCUUCUUCUUCU 1 :::::::.::::::::::: Target 103 GAAGAAGAGGAAGAAGAAGA 122	Cleavage	2
bol-miR838d	BoCNGC15	2.5	8.952	miRNA 21 ACUUGUUCUUCUUCUUCUUCU 1 :::.::::::::::::::: Target 135 UGAGAAAGAUGAAGAAGAAGA 155	Cleavage	2
bol-miR838d	BoCNGC16	3.0	6.192	miRNA 20 CUUGUUCUUCUUCUUCUUCU 1 :::: .::.:::::::::: Target 94 GAAGAGGAGGACGAAGAAGA 113	Translation	2
bol-miR838d	BoCNGC16	1.5	2.883	miRNA 20 CUUGUUCUUCUUCUUCUUCU 1 ::::::::.:::::.::.:: Target 124 GAACAAGAGGAAGAGGAGGA 143	Cleavage	2
bol-miR_new2	BoCNGC26	3.0	11.462	miRNA 20 UGGGAUUUAGUAUUUAGGAU 1 :::..::::::::::::: Target 639 ACCUGGAAUCAUAAAUCCUC 658	Cleavage	1

### Gene ontology enrichment analysis

Using Blast2GO (v.3.3.5), we assigned 31 gene ontology (GO) classes to 26 *BoCNGC* genes with BLAST matches to known proteins in the InterPro database. The majority of the genes were assigned to biological process (22), followed by molecular function (7) and cellular components (3). All genes encoded integral membrane components associated with ion channel activity for transmembrane transport. Notably, *BoCNGC1* was associated with salicylic acid biosynthesis, negative regulation of defence responses, regulation of plant-type hypersensitive responses, and responses to chitin. Additionally, *BoCNGC6* was associated with DNA-mediated transformation (Additional file 17).

The level 2 GO enrichment analysis revealed that all 26 BoCNGC proteins are integral cell membrane components, with four proteins (i.e., BoCNGC1, BoCNGC4, BoCNGC5, and BoCNGC17) forming cell parts, and two proteins (i.e., BoCNGC4 and BoCNGC5) forming macromolecular complexes (Additional files 18-a and 19). These proteins are involved in cellular processes associated with transport, binding, and transduction (Additional files 18-b and 19). The biological process category at GO level 2 indicated that BoCNGC1 and BoCNGC17 are associated with cell death and immune responses to stimuli, while another eight CNGCs, including BoCNGC19, are associated with localization (Additional files 18-c and 19). Moreover, we mapped the 26 annotated sequences to reference pathways in the Kyoto Encyclopedia of Genes and Genomes database [45]. Twenty-four of these genes were defined as “cyclic nucleotide gated channels”, and assigned to the “plant-pathogen interaction” pathway (Additional files 14 and 20).

### Expression patterns in different plant parts

**Fig. 6:**
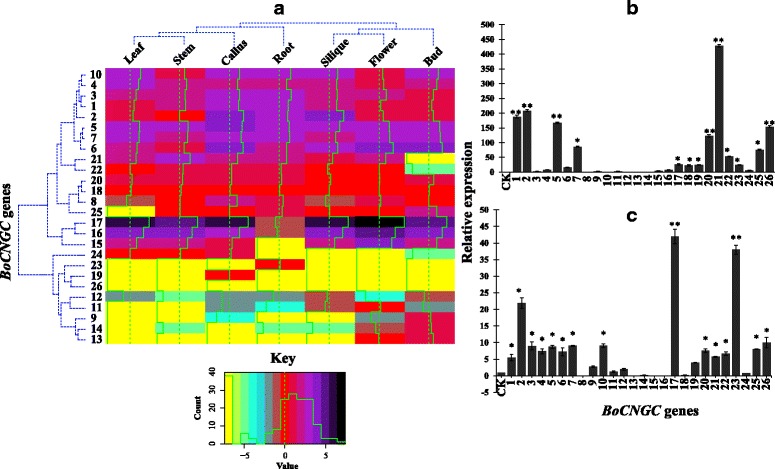
*BoCNGC* expression profiles in different plant parts and in response to stress. **a** Normalized *BoCNGC* expression levels (fragments per kilobase of exon model per million mapped reads) in different *Brassica oleracea* plant parts*.* **b** Effect of biotic stress on the expression levels of *BoCNGC* genes in the leaves of 25 days old seedlings inoculated with *Xanthomonas campestris* pv. *campestris* at 24 hpi. **c** Effect of abiotic stress on the expression levels of *BoCNGC* genes in the leaves of 25 days old seedlings subjected to cold stress at 4 °C for 24 h. The Y-axis indicates the relative expression levels of treated versus untreated control (CK). The error bars were calculated based on three biological replicates using standard deviation. The asterisks on bars represent the statistical significance of each gene at *p ≤* 0.01 based on LSD test

We investigated the steady-state *B. oleracea BoCNGC* expression patterns in seven tissues (i.e., leaf, stem, callus, root, silique, flower, and bud) using Illumina RNA-sequencing data from the Gene Expression Omnibus database. Of the 26 *BoCNGCs*, 19 were expressed at relatively high levels (fragments per kilobase of exon model per million mapped reads value >1) in at least one tissue, including 15 in the roots and siliques, 16 in leaves, and 17 in stems, buds, and flowers. The 19 genes were also expressed in calli (Fig. 6a). Some of the syntenic duplicates have diverged in expression patterns indicating sunfunctionalization. For example, BoCNGC26 and BoCNGC19 have very similar expression patterns. But their duplicates BoCNGC21 and BoCNGC20 now have different expression patterns. An additional investigation revealed that *BoCNGC17* and *BoCNGC16* were the most highly expressed genes, especially in flowers, implying they may be important for *Brassica* species development. Among the other genes, *BoCNGC3* was highly expressed in roots, while *BoCNGC2* was highly expressed in siliques and calli, suggesting that the expression of this genes is induced by wounding. Most of the Group III and IV genes were expressed at low levels in the leaves, stems, calli, roots, and siliques, while *BoCNGC26* was not expressed in any tissue.

A review of the reported expression profiles of orthologus *Arabidopsis CNGCs* in the tissues of wild and mutant plants suggest that a) the mRNAs of this gene family are expressed in all plant tissues, b) expression in leaves is greater than in roots, stem and flower, c) group-I, II and IV *CNGCs* are highly expressed in flowers and apex of *Arabidopsis* mutants (Additional file 21) [46]. Some of these observations have been confirmed during earlier investigation of *CNGC* mutants in *Arabidopsis* plants, for example *AtCNGC1* [47]. Moreover, the expression patterns of *BoCNGC1* and *BoCNGC7* were consistent with their orthologs (*ATCNGC10* and *ATCNGC5*), which are highly expressed in roots than leaves [7]. Our results are also corroborated by the findings of Borsics et al. [6], showing that *AtCNGC10* mutant plants exhibited reduced mRNA levels in flower than its closest related member *AtCNGC13* and WT plants.

### Expression patterns in response to abiotic and biotic stresses

Based on the *BoCNGC* expression patterns in different tissues, we attempted to determine whether these genes were associated with plant defence responses, especially against race- and species-specific *Brassica* pathogens. Therefore, we analysed the *BoCNGC* expression profiles in the shoots of 25-day-old *Brassica* plants infiltrated with *Xanthomonas campestris* pv. *campestris* (Xcc). The *BoCNGC* expression levels at 24 h post-inoculation are presented in Fig. 6b. The pathogen induced considerable changes to *BoCNGC* expression levels, including the up-regulation of the expression of 10 *BoCNGC* genes in infiltrated seedlings, with the highest levels observed for *BoCNGC21*. This was followed by *BoCNGC2* and *BoCNGC1* from Group I, *BoCNGC5* and *BoCNGC7* from Group II, and *BoCNGC26* and *BoCNGC20* from Group IV-b. Interestingly, none of the Group III and IV-a genes were affected.

We also examined the *BoCNGC* expression levels under cold conditions. The expression of 13 of the 26 *BoCNGC* genes was up-regulated in cold-stressed plants, although the expression levels were lower than the levels induced by Xcc (i.e., biotic stress) (Fig. 6c). The expression levels of genes from Groups I, II, and IV were significantly induced by cold stress, with the highest levels observed for *BoCNGC17* and *BoCNGC23*. In contrast, the Group III *BoCNGCs* were expressed at low levels or not at all under cold conditions. Moreover, most of the duplicated gene pairs and genes encoding interacting proteins produced similar expression patterns (especially in response to Xcc). The exception was *BoCNGC24* whose expression was not significantly up-regulated like its duplicates (i.e., *BoCNGC21* and *BoCNGC22*).

The expression patterns of many *BoCNGCs* under pathogen stress were consistent with the expression patterns of their *Arabidopsis* orthologs obtained from the AtGenExpress project (Additional file 22) [46]. The involvement of group-IV CNGCs in disease resistance and hyper-sensitivity has been documented earlier [21, 22]. However, the cumulative profiles of group-I and IV CNGCs in *Arabidopsis* seedlings showed apposite trend of down-regulation by cold stress at 4 °C for 24 h, showing specie-specific divergence of expression pattern.

## Discussion

The *CNGC* gene family has been reported for many agriculturally important plants [17, 18, 20]. However, a genome-wide identification and annotation of *CNGC* genes has not been reported for *B. oleracea*. In this study, we identified 26 *B. oleracea CNGC* genes, and determined that the *BoCNGC* gene family is larger than the *CNGC* families of most of the reported crops [4]. The isoelectric point (*pI*) and charge of a protein is important for solubility, subcellular localization, and interaction, depending on both insertion and deletions between orthologs, and the ecology of the organism [48]. It is reported that proteins in cytoplasm possess an acidic *pI* (*pI* < 7.4), nuclear proteins have more neutral *pI* (7.4 < *pI* < 8.1), while those in membrane have more basic *pI* [48]*,* where basic residues located on either side of membrane spanning region play a role in the stabilization of the protein in membrane [49]. The net charge of a protein is a fundamental physical property, and its value directly influences the solubility, aggregation, and crystallization of the protein [50]. The 26 BoCNGCs were localized to membranes, greatly varied in physicochemical properties, and will theoretically participate in basic buffers. These variations reflects the changes in protein composition, and their effects on association of receptors with charged ligands, folding and stability, solubilization and precipitation, and selective transport of ions in protein channels [50].

Homologous genes within the same taxonomic group are assumed to exhibit similar structural, functional, and evolutionary properties, which may help clarify the role(s) of *B. oleracea CNGC* genes. Because of the close relationship between *B. oleracea* and *A. thaliana*, the *BoCNGC* genes were highly similar (>90%) to the corresponding *AtCNGC* genes regarding plant CNGC-specific domains, amino acid compositions, gene structures, and phylogenetic classifications. Interestingly, we revealed the absence of the CaMBD and IQ domain in BoCNGC18 and BoCNGC19, which raised the possibility that these were abnormal CNGC proteins. However, we found that many of their homologs in *A. thaliana*, pear and *B. rapa* reportedly lack the CaMBD [18]. Similar to other CNGCs, these proteins have regular 3D structural and membrane topologies, with conserved binding sites for cGMP/cAMP. Furthermore, the presence of conserved nickel- and zinc-binding sites suggests that BoCNGC18 and BoCNGC19 may have lost their secondary domains during evolution, but gained functional diversity. Additional research is required to clarify this point.

Proteins undergo post-translational modifications (PTMs), which increase the range of their functions through different mechanisms [51]. The associated PTMs likely affected protein function, localization, and stability, as well as the dynamic interactions with other molecules [52]. Following gene annotations and phylogenetic analyses, we predicted the presence of multiple PTM sites in BoCNGCs. Apart from evolutionarily conserved PTMs, other types of modification sites were detected in BoCNGCs, which diversified the functions and underlying mechanisms of CNGC-specific PTMs. Protein–protein interaction networks provide a base for systematic understanding of cellular processes that can be used to filter and assess the functional genomics data and provide an instinctive platform to annotate the structures, functions and evolutionary properties of proteins [53]. Using two different approaches, and orthologous *Arabidopsis* CNGCs as a reference, we found that most CNGCs were associated with various protein–protein interaction networks involving CNGCs and other proteins related to light signalling [54], regulation of enzyme activities [55] and cellular processes [56], brassinosteroid signal transduction [57], and resistance against pathogens [58]. These aanalyses can offer new information for future experimental research and provide cross-species predictions for efficient interaction mapping [53]. Additionally, of the 26 *BoCNGC* genes, nine included target sites for diverse groups of novel and conserved miRNAs. These miRNA families are highly conserved in *Brassicaceae* species, where they are expressed in leaves, siliques, and flowers. These miRNAs are reported to function in regulation of genes related to growth (miR157/171/824) [59], *Brassica*-specific stomatal organization (miR824), pollen development (miR824) [60], abiotic stress tolerance, and plant–pathogen interactions (miR398) [61].

Gene duplications during evolution increase the genomic content and expand gene functions to optimise the adaptability of plants [25]. *Brassica oleracea* is an ancient polyploid, whose genome underwent a WGT event approximately 16 million years ago, after diverging from *A. thaliana*, followed by large-scale chromosomal re-arrangements (i.e., re-diploidisation). As a member of the classical triangle of U [62], the assembled genome of *B. oleracea* (540 Mb) is larger than that of its sister species, *B. rapa* (312 Mb) [63] that diverged from a common ancestor nearly 4 million years ago [64]. The less number of *CNGC* genes in *Brassica* genomes suggest that most of the duplicated gene copies were lost post-polyploidization. Reversion of the few duplicated *CNGC* genes to single copy might be due to neutral loss of unnecessary duplicates over time. Another possible explanation could be that CNGC proteins participate in dosage sensitive interactions that is affected by the copy number of each protein subunit (gene balance hypothesis) [24]. Synteny analysis revealed that more than 80% of the *BoCNGC* genes are located in conserved syntenic blocks, which lost and gained some genes. These results are consistent with the findings of Liang et al. [65]. We presume that functionally redundant gene copies are reportedly lost after genome duplication events, while some copies of functionally important genes are kept [51]. Our findings suggest that the WGT and segmental duplication events were important for the expansion of the *B. oleracea CNGC* family, where tandem duplications only affected the expansion of Group IV-b. Altogether, the conservation of *CNGC* genes after substantial genome reshuffling suggests that these genes are crucial for plant development [66]. Finally, the detailed analyses of gene expression in different tissues and under stress conditions further supported the importance of various *CNGC* genes for *B. oleracea* growth, development, and survival. To the best of our knowledge, this manuscript is the first to describe a comprehensive and systematic analysis of the *B. oleracea CNGC* gene family. The generated data may be useful for constructing protein–protein interaction networks and experimentally validating the miRNA targets, which regulate the development of *B. oleracea*. Besides, our results might help in understanding the functions of BoCNGCs related to the regulation of signal transduction pathways, and elucidate the expression profiles of the corresponding genes during plant development and stress responses. The results of the bioinformatics and comparative genomic analyses are also valuable for studying CNGC protein functions, with potential implications for the economic, agronomic, and ecological enhancement of *B. oleracea* and other *Brassica* species.

## Conclusions

In conclusion, this work is the first comprehensive and systematic analyses of *CNGC* gene family in *B. oleracea*. There are 26 *CNGC* genes in *B. oleracea*, which are classified into 4 groups (I-IV) and fractionated into three sub-genomes; this gene family appears to have expanded through WGT, segmental and tandem duplication events; the BoCNGC gene family is under positive selection pressure. All the BoCNGC protein sequences contain a CNGC specific domain CNBD that comprises a PBC and a “hinge” region, featured by a stringent motif: LI]-X(2)-[GSE]-X-[VFIY]-X-G-X(0,1)-[DE]-L-L-X-W-X-[LQ]-X(10,20)-S-X-[SAR]-X(7)-[VTI]-E-[AG]-F-X-L. This study provided comprehensive information about domain structure, exon-intron structure, and the phylogenetic tree and expression analysis of *CNGC* genes in Chinese cabbage. These data are useful to construct protein-protein interaction network and experimentally validate the miRNA targets, which regulates and induces multiple responses in *B. oleracea*. The bioinformatics analysis and comparative genomic analysis also provides valuable information in the study of CNGC protein functions for the improvement of the economic, agronomic, and ecological benefits of Chinese cabbage. Furthermore, this study assists to elucidate the functions of differentially expressed candidate genes in the regulation of signal transduction pathway, plant development and stress resistance in *B. oleracea*.

## Methods

### Identification of *Brassica oleracea CNGC* genes

To identify the *B. oleracea CNGC* genes, 20 *Arabidopsis* CNGC protein sequences obtained from TAIR10 (https://www.arabidopsis.org/) [67] were used as queries to perform a homology-based search of the Ensembl Plants database (genome version v.2.1) [68]. This search was conducted with the default parameters of the BLASTP program. All non-redundant protein sequences were retrieved, and their domains were analysed with online servers: Simple Modular Architecture Research Tool (SMART) (http://smart.embl-heidelberg.de/) [69] and the Conserved Domains Database (CDD) (http://www.ncbi.nlm.nih.gov/Structure/cdd/wrpsb.cgi) [70]. The analyses were completed with the default cut-off parameters. Sequences containing the cNMP/CNBD (IPR000595) and transmembrane/ion transport protein (PF00520) domains as well as a plant CNGC-specific motif in the PBC and hinge region within the CNBD were recognized as CNGC proteins. The identified *BoCNGC* genes were named according to their positions in the phylogenetic tree.

### Protein characterisation and amino acid properties

Details regarding gene and protein lengths as well as chromosomal locations were obtained from the Ensembl Plants database. Amino acid properties, including charge, molecular weight (kDa), aliphatic and instability indices, isoelectric points (pI), and grand average of hydropathy (GRAVY), were determined using the online available ProtParam tool (http://web.expasy.org/protparam/) [71]. The PTM sites were predicted with the ScanProsite web server (http://prosite.expasy.org/scanprosite/) [30].

### Multiple sequence alignment and phylogenetic analysis

The identified CNGC proteins were aligned using the default settings of the ClustalX 2.0 program [72]. The conserved CNGC-specific domains were manually checked and shaded with the DNAMAN program (version 6.0.3.40; Lynnon Corporation, Quebec, Canada). The BoCNGC protein sequences were also aligned with CNGC sequences from *A. thaliana* and *B. rapa* (downloaded from the *Brassica* database; http://brassicadb.org/brad/) [73] using the default settings of the ClustalX 2.0 program. The alignments were viewed with the GeneDoc program [74]. A phylogenetic tree was constructed using the maximum likelihood method of MEGA 6.0 (1000 bootstrap replications) [75].

### Chromosomal locations and gene duplication events

Details regarding the chromosomal locations of the *BoCNGC* genes were obtained from the Ensembl Plants database. The Plant Genome Duplication Database [76] was searched to identify segmentally duplicated genes. *BoCNGC* genes were defined as tandemly duplicated if the distance between the homologous loci was <50 kb [65]. The syntenic relationships among *BoCNGCs*, *AtCNGCs*, and *BrCNGCs* were evaluated using the Search Syntenic Genes tool in Bolbase [77].

### Gene structure, motif composition, and prediction of three-dimensional models

Gene exon/intron structures were predicted with the Gene Structure Display Server (version 2.0) [78], with genomic and coding sequences as the input data. The conserved motifs in the CNGC sequences were identified using the Multiple Expectation Maximization for Motif Elicitation suite and the Motif Alignment and Search Tool [28] with the following parameters: optimal motif width: 6–200; maximum number of different motifs: 10. The detected motifs were annotated with Pfam [29]. Gene ontology enrichment analysis was performed using Blast2GO (v.3.3.5) [79].

### Analysis of microRNA target sites and protein–protein interactions

The *B. oleracea* miRNA sequences obtained from the miRBase database at http://mirbase.org/ [80]. To detect potential miRNA target sites within the *BoCNGC* genes, the obtained miRNAs were analysed with the psRNATarget server (http://plantgrn.noble.org/psRNATarget/) [44] The information about protein-protein interaction, and available mutant information for *Arabidopsis CNGC*-encoded proteins was obtained from STRING (v10) [36] and AtPID (http://www.megabionet.org/atpid/webfile/query.php).

### Analysis of *BoCNGC* transcriptome data

To investigate the *BoCNGC* expression profiles, we used the Illumina RNA-sequencing data available in the Gene Expression Omnibus database (accession number GSE42891) [24]. Transcript abundance was calculated as fragments per kilobase of exon model per million mapped reads, and the resulting values were log_2_ transformed. A hierarchical cluster was created and a heat map was generated with R language program [81].

### Experimental conditions and quantitative real-time polymerase chain reaction assay

We used a quantitative real-time polymerase chain reaction (qRT-PCR) to quantify the *BoCNGC* expression levels in response to biotic (bacterial pathogen) and abiotic (cold) stresses. Cabbage (*B. oleracea* var. *capitata* L.) seedlings were grown for 25 days in a greenhouse at 23 ± 2 °C under natural light. For the cold stress treatment, seedlings were incubated at 4 °C for 24 h. For the bacterial infection, Xcc was first cultured in medium B [82] at 28 °C. Cells were collected by centrifugation, re-suspended in sterilized distilled water, and adjusted to an optical density at 600 nm of 0.1. The midvein of the first fully opened leaf (just above the petiole) was inoculated with the Xcc suspension using a 1-ml syringe. Sterilized ddH_2_O was used as the control solution. The treated plants were returned to the greenhouse and sampled 24 h later. The extraction of RNA and synthesis of cDNA were completed as previously described [20]. Gene-specific primers were designed with Primer 5.0 (Additional file 23). The qRT-PCR was conducted using a StepOne Real-Time PCR System (Applied Biosystems, USA) and SYBR Premix Ex Taq reagents (TAKARA, Japan) as described by Kabouw et al. [83]. Finally, the 2^−ΔΔCt^ method [84] was used to calculate the relative gene expression values, which were subsequently transformed to log_2_- expression ratios and plotted in figures. Each experiment was performed with three technical replicates. The *Actin* gene (AF044573) was used as an endogenous control.

### Statistical analysis

The RT-qPCR expression data was subjected to analysis of variance (ANOVA) using computer statistical package (SAS software SAS Institute, Cary, NC). Least significant difference (LSD) test at *p ≤* 0.01 was used to check the significant differences between the expression levels of different *BoCNGC* genes compared to control.

## Additional files

Additional file 1: List of truncated gene accessions discarded during preliminary investigation. (XLSX 9 kb)

Additional file 2: Multiple sequence alignment of CNGC proteins from *B. oleracea*, *B. rapa* and *A. thaliana*. (PDF 2222 kb)

Additional file 3: Phylogenetic tree of CNGC proteins from *B. oleracea* (encoded by *BoCNGCs*). A multiple sequence alignment was performed using ClustalX 2.0 program with default settings. Maximum likelihood (ML) tree was create with MEGA 6.0, under the Jones-Taylor-Thornton (JTT) model. The bootstrap values from 1000 resampling are given at each node. (PDF 201 kb)

Additional file 4: Phylogenetic tree of *CNGC* genes from *Arabidopsis* (*AtCNGCs*). A multiple sequence alignment was performed using ClustalX 2.0 program with default settings. Maximum likelihood (ML) tree was create with MEGA 6.0, under the Jones-Taylor-Thornton (JTT) model. The bootstrap values from 1000 resampling are given at each node. (PDF 180 kb)

Additional file 5: Syntenic ancestral block structure between *A. thaliana* and three sub-genomes of *B. oleracea* and *B. rapa*. (XLSX 10 kb)

Additional file 6: Synteny of *BoCNGC* in other plant species. (XLSX 18 kb)

Additional file 7: Primary domain architecture of BoCNGC proteins. Information about domain annotation is obtained from SMART database. (PDF 339 kb)

Additional file 8: Multiple sequence alignment of BoCNGC proteins. Multiple sequence alignment was performed by clustalX2and viewed by GeneDoc software package. (PDF 767 kb)

Additional file 9: Multiple sequence alignment of *CNGC*-encoded proteins of *Arabidopsis* and *B. oleracea*. Multiple sequence alignment was performed by clustal X2 and viewed by GeneDoc software package. (PDF 1208 kb)

Additional file 10: Multiple sequence alignment of CNGC-encoded proteins of *B. oleracea* and *B. rapa.* Multiple sequence alignment was performed by clustal X2 and viewed by GeneDoc software package. (PDF 1436 kb)

Additional file 11: Schematic diagram showing the structures of *Arabidopsis CNGC* family genes. The exons-introns indicated as red boxes and black lines respectively, and the intron phases are displayed as numbers [0, 1 and 2]. The lengths of each exon and intron can be mapped to the scale given in the bottom. (PDF 154 kb)

Additional file 12: Functional annotation of the identified conserved MEME motifs. (XLSX 10 kb)

Additional file 13: Web logos of MEME-identified conserved functional motifs in BoCNGC proteins. The heights of the amino acids indicates the degree of conservation. (PDF 423 kb)

Additional file 14: KO pathway associated with plant-pathogen interaction (K05391). The pathway map was obtained from http://www.kegg.jp/kegg/kegg1.html. Details of *BoCNGC* genes allocated to K05391 pathway are given in Additional file 20. (PDF 126 kb)

Additional file 15: Table showing the details of protein-protein interaction, and available mutant information for *Arabidopsis CNGC*-encoded proteins. The information was obtained from AtPID. (XLSX 37 kb)

Additional file 16: The potential miRNA targets in the set of 26 *BoCNGC* transcripts using cut-off threshold of 5 in the search parameters. (XLSX 14 kb)

Additional file 17: GO term enrichment analysis of *BoCNGC* genes for Molecular function (MF), Biological process (BP) and Cellular component (CC). (XLSX 17 kb)

Additional file 18: Distribution of *BoCNGC* genes in major functional terms (GO terms Level 2) for categories Molecular Function **(a)**, Biological Process **(b)** and Cellular Component **(c)**. The details are given in Additional file 19. (PDF 97 kb)

Additional file 19: GO term enrichment analysis at level 2 for category: P: Biological process, F: Molecular function and C: Cellular component. (XLSX 9 kb)

Additional file 20: Reference KO pathway associated with *BoCNGC* genes. The pathway map was obtained from http://www.kegg.jp/kegg/kegg1.html. (XLSX 10 kb)

Additional file 21: Cumulative values of expression for *Arabidopsis CNGC* genes in different developmental samples. The expression data for 21 days old of wild type and mutant plants was obtained from Schmid et al. [48]. The information about different genotype mutants is given below the figures. (PDF 225 kb)

Additional file 22: Cumulative values of expression for *Arabidopsis CNGC* genes in response to pathogen (biotic) and cold (Abiotic) stress. The expression data for 21 days old of wild type and mutant plants was obtained from Schmid et al. [48]. (PDF 294 kb)

Additional file 23: List of primers used for gene expression via qRT-PCR. (XLSX 12 kb)

## Abbreviations

AMD: Amidation
ASN: N-glycosylation
CaM: calmodulin
CaMBD: CaM-binding domain
cAMP: cyclic adenosine monophosphate
cGMP: cyclic guanosine monophosphate
CK2: Casein kinase II
CNBD: cNMP-binding domain
CNGC: Cyclic nucleotide-gated ion channel
IQ: Isoleucine-glutamine
IT: Ion transport
LEU: Leucine zipper pattern
MYR: N-myristoylation
PBC: Phosphate-binding cassette
p*I*: Isoelectric point
PKC: Protein kinase C
PTM: Post-translational modification
RGD: Cell attachment sequence
TYR: Tyrosine kinase
WGD: Whole-genome duplication
Xcc: *Xanthomonas campestris* pv. *campestris*
