# Supplementary material for: Comprehensive genomic analysis of the CNGC gene family in Brassica oleracea: novel insights into synteny, structures, and transcript profiles
Source: BMC Genomics. 2017 Nov 13;18:869. doi: 10.1186/s12864-017-4244-y (PMC5683364; doi:10.1186/s12864-017-4244-y)
Supplement: Supplementary file 2 — Multiple sequence alignment of CNGC proteins from B. oleracea, B. rapa and A. thaliana. (PDF 2222 kb) [file 12864_2017_4244_MOESM2_ESM.pdf]

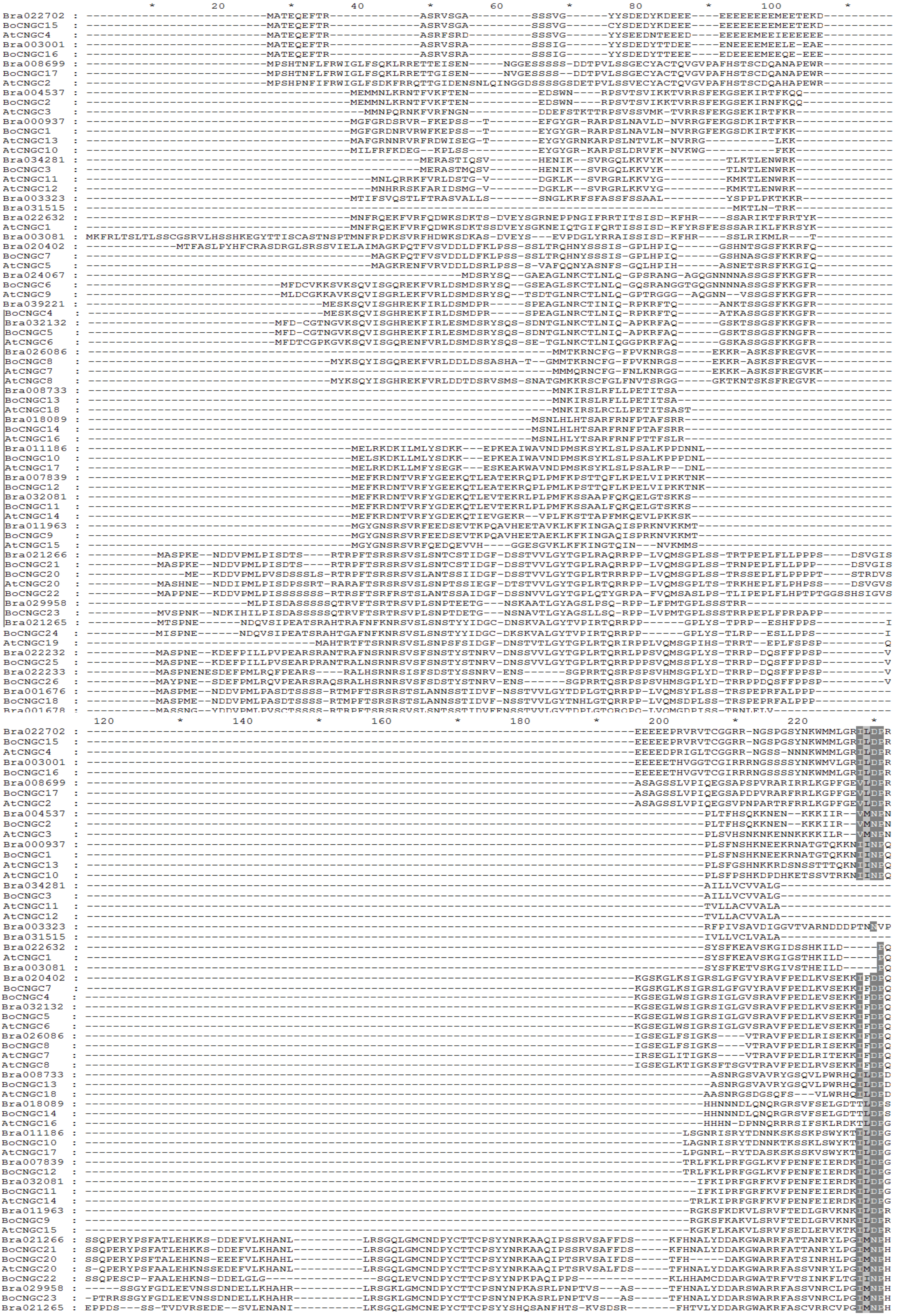
20
40
60
80
100
Bra022702
BoCNGC15
AtCNGC4
Bra003001
BoCNGC16
Bra008699
BoCNGC17
AtCNGC2
Bra004537
BoCNGC2
AtCNGC3
Bra000937
BoCNGC1
AtCNGC13
AtCNGC10
Bra034281
BoCNGC3
AtCNGC11
AtCNGC12
Bra003323
Bra031515
Bra022632
AtCNGC1
Bra003081
Bra020402
BoCNGC7
AtCNGC5
Bra024067
BoCNGC6
AtCNGC9
Bra039221
BoCNGC4
Bra032132
BoCNGC5
AtCNGC6
Bra026086
BoCNGC8
AtCNGC7
AtCNGC8
Bra008733
BoCNGC13
AtCNGC18
Bra018089
BoCNGC14
AtCNGC16
Bra011186
BoCNGC10
AtCNGC17
Bra007839
BoCNGC12
Bra032081
BoCNGC11
AtCNGC14
Bra011963
BoCNGC9
AtCNGC15
Bra021266
BoCNGC21
BoCNGC20
AtCNGC20
BoCNGC22
Bra029958
BoCNGC23
Bra021265
BoCNGC24
AtCNGC19
Bra022232
BoCNGC25
Bra022233
BoCNGC26
Bra001676
BoCNGC18
120
140
160
180
200
220

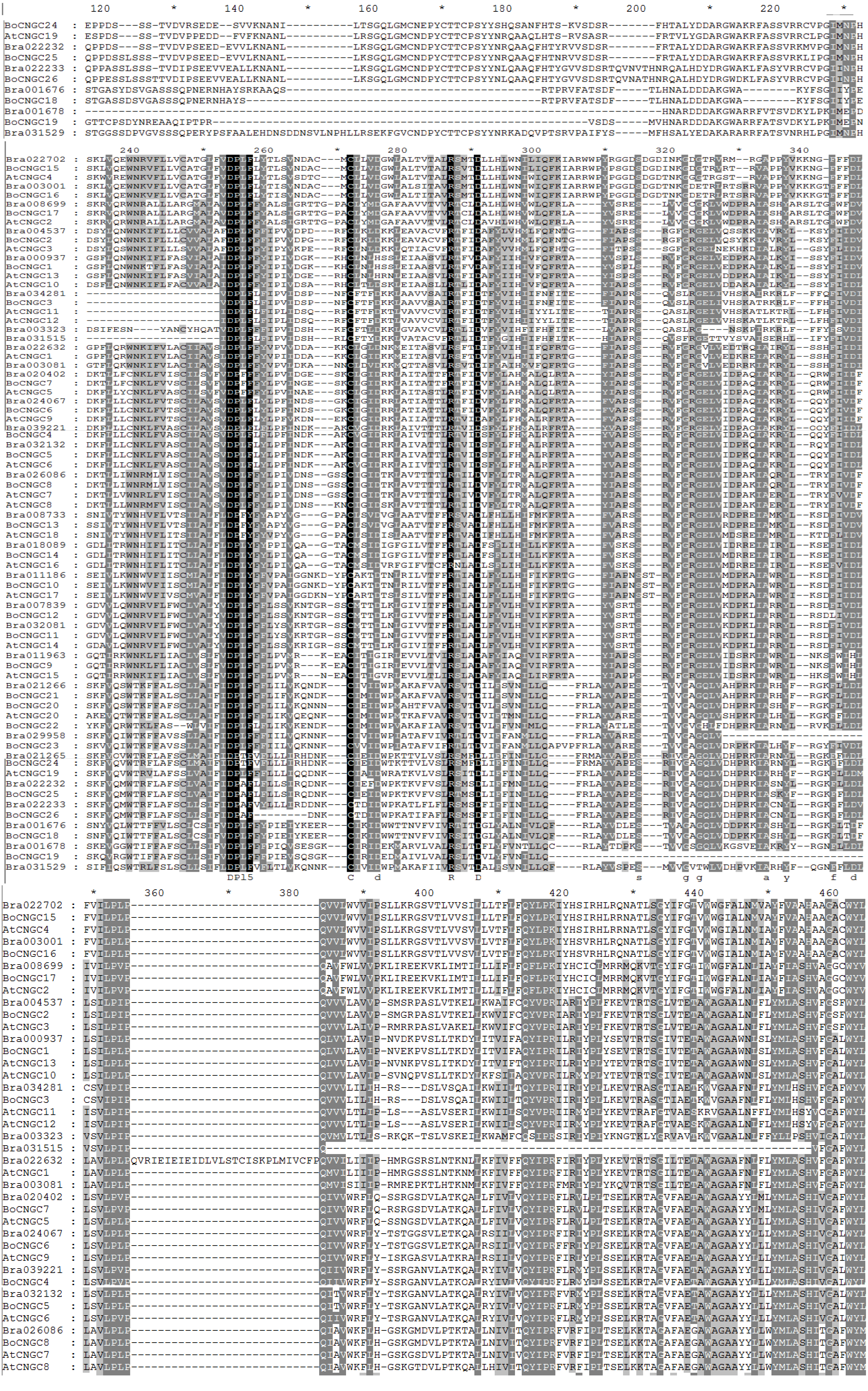

120 140 160 180 200 220
BoCNGC24
AtCNGC19
Bra022232
BoCNGC25
Bra022233
BoCNGC26
Bra001676
BoCNGC18
Bra001678
BoCNGC19
Bra031529
240 260 280 300 320 340
Bra022702
BoCNGC15
AtCNGC4
Bra003001
BoCNGC16
Bra008699
BoCNGC17
AtCNGC2
Bra004537
BoCNGC2
AtCNGC3
Bra000937
BoCNGC1
AtCNGC13
AtCNGC10
Bra034281
BoCNGC3
AtCNGC11
AtCNGC12
Bra003323
Bra031515
Bra022632
AtCNGC1
Bra003081
Bra020402
BoCNGC7
AtCNGC5
Bra024067
BoCNGC6
AtCNGC9
Bra039221
BoCNGC4
Bra032132
BoCNGC5
AtCNGC6
Bra026086
BoCNGC8
AtCNGC7
AtCNGC8
Bra008733
BoCNGC13
AtCNGC18
Bra018089
BoCNGC14
AtCNGC16
Bra011186
BoCNGC10
AtCNGC17
Bra007839
BoCNGC12
Bra032081
BoCNGC11
AtCNGC14
Bra011963
BoCNGC9
AtCNGC15
Bra021266
BoCNGC21
BoCNGC20
AtCNGC20
BoCNGC22
Bra029958
BoCNGC23
Bra021265
BoCNGC24
AtCNGC19
Bra022232
BoCNGC25
Bra022233
BoCNGC26
Bra001676
BoCNGC18
Bra001678
BoCNGC19
Bra031529
DP15
C d R D s g g a y f d
360 380 400 420 440 460
Bra022702
BoCNGC15
AtCNGC4
Bra003001
BoCNGC16
Bra008699
BoCNGC17
AtCNGC2
Bra004537
BoCNGC2
AtCNGC3
Bra000937
BoCNGC1
AtCNGC13
AtCNGC10
Bra034281
BoCNGC3
AtCNGC11
AtCNGC12
Bra003323
Bra031515
Bra022632
AtCNGC1
Bra003081
Bra020402
BoCNGC7
AtCNGC5
Bra024067
BoCNGC6
AtCNGC9
Bra039221
BoCNGC4
Bra032132
BoCNGC5
AtCNGC6
Bra026086
BoCNGC8
AtCNGC7
AtCNGC8

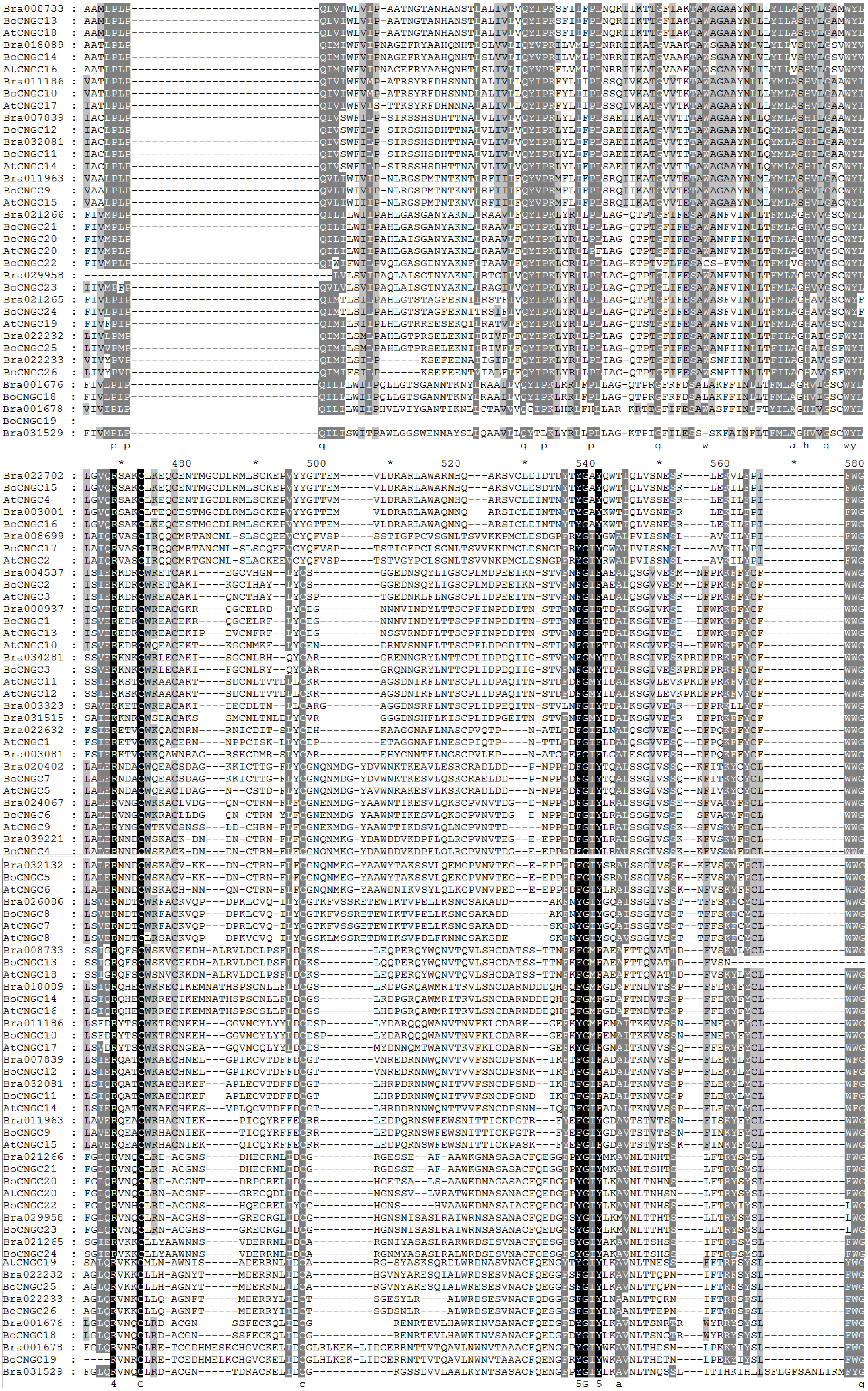

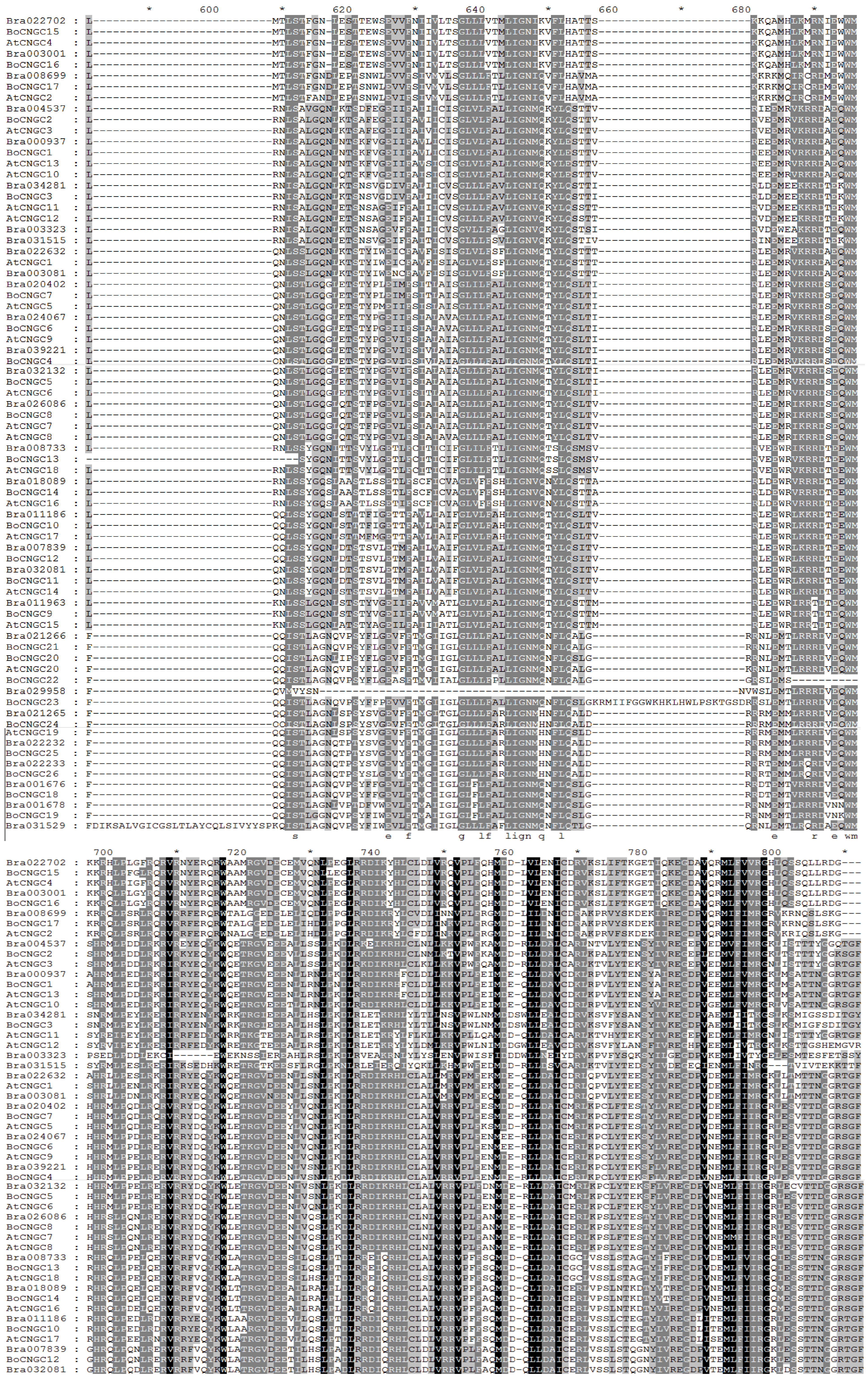

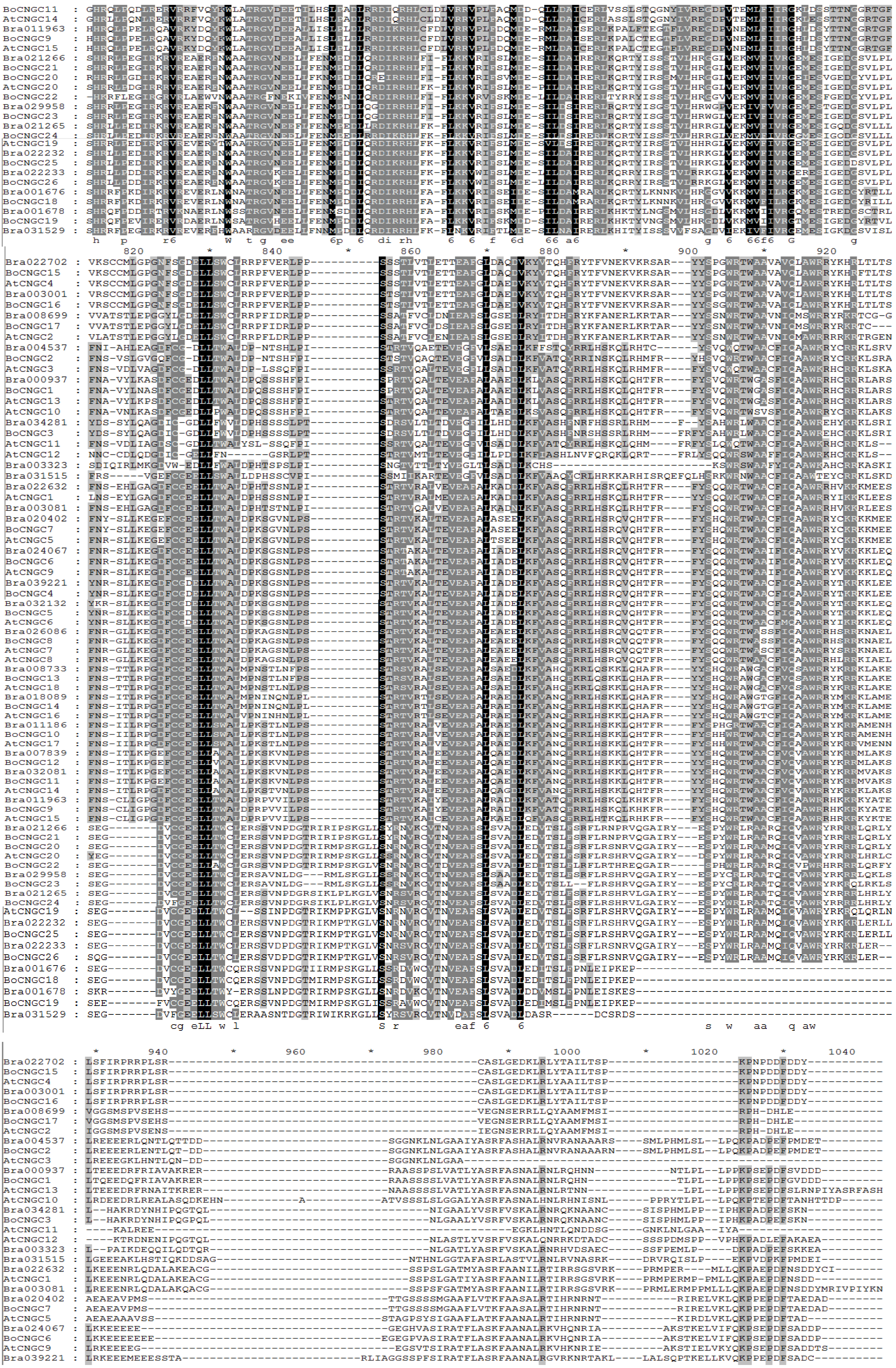

```
BoCNGC4    : LRKEEEMEEESSAA--------------------------RLIAGGSSPYSIRATFLASRFAANALRGVRKNRTAKL----LALSQPTKELLKVQKPPEPDFSADC----------
Bra032132  : LRKEEEEEEEESAA--------------------------RLIAGGS-PYSIRATFLASKFAANALRSVHKNRIRKS----NLAPPSTKELVKFQKPPEPDFSADC----------
BoCNGC5    : LRKEEEEEEE-TAA--------------------------RLIAGGS-PYSIRATFLASKFAANALRSVHKNRIRKS----NLAPPSTKELVKFQKPPEPDFSADC----------
AtCNGC6    : LRKEEEEEEA-AAA--------------------------SVIAGGS-PYSIRATFLASKFAANALRSVHKNRTAKS----TLLLSSTKELVKFQKPPEPDFSAEDH---------
Bra026086  : RRIEEEEDEMGYED-EYDDD-----------DAEEEDERTPVFTRTESSSRLRSTIFASRFAANALKG-HRLRST----------ESSKRLLNLQKPPEPDFDAE-----------
BoCNGC8    : RRIEEEEDEMGYED-EYDDD-----------DAEEEDERTPVFTRTESSSRLRSTIFASRFAANALKG-HRLRST----------ESSKRLLNLQKPPEPDFDAE-----------
AtCNGC7    : RRIEEKEEELGYED-EYDD---------------ESDKRPMVITRSESSSRLRSTIFASRFAANALKG-HRLRSS----------ESSKTLINLQKPPEPDFDAE-----------
AtCNGC8    : RRKEEEEEEMDYEDDEYYDD-----------N--MGGMVTRSDSSVGSSSTLRSTVFASRFAANALKG-HKLRVT----------ESSKSLMNLTKPSEPDFEALDTDDLN-----
Bra008733  : LSLHESSGYYYRDETGYNEEGDE--ENYYGSDDDDFEGERLSVDNTNNSQNLGATMLASKFAANTRRGTNQKASSSTSAGKKDGSSNSLKMPQLFKPDEPDFSMDKEDV-------
BoCNGC13   : LSLHESSGYYYRDETGYNEEGDE--ENYYGSDDDDFEGERLSVDNTNNSQNLGATMLASKFAANTRRGTNQKASSSSSTGKKDGSSNSLKMPQLFKPDEPDFSMDKEDV-------
AtCNGC18   : LSLHESSGYYYPDETGYNEEDEETREYYYGSDE---EGG--SMDNTN----LGATILASKFAANTRRGTNQKASSS-STGKKDGSSTSLKMPQLFKPDEPDFSIDKEDV-------
Bra018089  : LARQEEGDDYYYDDDD---------DDQYGGEDMPESSNN-VDDNSSNSQNLSATILASKFAANTKRGVLGNQRGS---SRIDPDDPTLKMPKMFKPEDPGFF-------------
BoCNGC14   : LARQEEGDDYYYDDDD---------DDQYGGEDMPESSNN-VDDNSSNNQNLSATILASKFAANTKRGVLGNQRGS---SRIDPDDPTLKMPKMFKPEDPGFF-------------
AtCNGC16   : LARQEEEDDYFYDDDG---------DYQF-EEDMPESNNNNGDENSSNNQNLSATILASKFAANTKRGVLGNQRGS---TRIDPDHPTLKMPKMFKPEDPGFF-------------
Bra011186  : LTAVESKQS-DEDDEEEE----VVVRKVVEEEEEGVGSSPK------TKMNIGVMVLASRFAANTRRGVAAQRVKD------------VEMPRFKKPEEPDFSAEPDD--------
BoCNGC10   : LTAVESKQS-DEE-EEEE----VVVGKVVEEEEEGVGPSPN------TKMNIGVMVLASRFAANTRRGVAAQRVKD------------VEMPRFKKPEEPDFSAEPDD--------
AtCNGC17   : LTAIESMEN-EEG----E----VGEELVVVEEEECVEESPR------TKMNLGVMVLASRFAANTRRGVAAQRVKD------------VELPRFKKPEEPDFSAEHDD--------
Bra007839  : LSLAESYSSYEEEEALAA----AAAEEIMSQQEERQSSTPSRHHTSIGKPHFAATVLASRFAKNTRR--ASRKMKD------------VDVPMLPKPEEPDFSVDAD---------
BoCNGC12   : LSLAESYSSYEEEEALAT----AAAEEIMSQQEERQSSTPSRHHTSIEKPHFAATVLASRFAKNTRR--ASRKIKD------------VDVPMLPKPEEPDFSVDAD---------
Bra032081  : LSLAESFSSYEEEEAVA-----VAAEEIMSQQGERQSSNPSRHSTSIGKPHFAATILASRFAKNTRK--TAHKLKD------------VEVPMLPKPDEPDFSVDGD---------
BoCNGC11   : LSLAESFSSYEEEEAVA-----VAAEEIMSQQGERQSSNPSRHSTSIGKPHFAATILASRFAKNTRK--TAHKLKD------------VEVPMLPKPDEPDFSVDGD---------
AtCNGC14   : LSLAESFSSYDEEEAVA-----VAATEEMSHEGEAQSGAKARHHTSNVKPHFAATILASRFAKNTRR--TAHKLKD------------VEIPMLPKPDEPDFSVDD----------
Bra011963  : LRVKEEFQCMFET---------------------------------------ASM-VRLNSGKFTRSGSDSGMVSS-----------------IQKPVEPDFSSE-----------
BoCNGC9    : LRVKEEFQCMFET---------------------------------------ASM-VRLNSGKFTRSGSDSGMVSS-----------------IQKPVEPDFSSE-----------
AtCNGC15   : LRAKEEFHYRFEA---------------------------------------ATARLAVNGGKYTRSGSDSGMMSS-----------------IQKPVEPDFSSE-----------
Bra021266  : TAQS-SYSL-----------------------------------------------------------------------------------------------------------
BoCNGC21   : TAQS-SYSL-----------------------------------------------------------------------------------------------------------
BoCNGC20   : TDQS-SYSL-----------------------------------------------------------------------------------------------------------
AtCNGC20   : TPQS-SYSL-----------------------------------------------------------------------------------------------------------
BoCNGC22   : SAQS-SYSL-----------------------------------------------------------------------------------------------------------
Bra029958  : TAQKKQYSSELIQKDMAKT-------------------------------------------------------------------------------------------------
BoCNGC23   : TAQKKQYSLELIKKDMAKT-------------------------------------------------------------------------------------------------
Bra021265  : TAQSTSRR------------------------------------------------------------------------------------------------------------
BoCNGC24   : TAQSTSRR------------------------------------------------------------------------------------------------------------
AtCNGC19   : TAHSNSNR------------------------------------------------------------------------------------------------------------
Bra022232  : LQDNRVMKEMSDVRIGYRLSWSGVVEGSGKQLDTRGSS------------------------------------------------------------------------------
BoCNGC25   : LQDNRVMKEMSDVPIGYRLSWSGVVEGSGK--------------------------------------------------------------------------------------
Bra022233  : KKQNGDIDE-----------------------------------------------------------------------------------------------------------
BoCNGC26   : RKQNGEMDE-----------------------------------------------------------------------------------------------------------
Bra001676  : --------------------------------------------------------------------------------------------------------------------
BoCNGC18   : --------------------------------------------------------------------------------------------------------------------
Bra001678  : --------------------------------------------------------------------------------------------------------------------
BoCNGC19   : --------------------------------------------------------------------------------------------------------------------
Bra031529  : --------------------------------------------------------------------------------------------------------------------

                    *      1060         *      1080
Bra022702  : -------------------------------------
BoCNGC15   : -------------------------------------
AtCNGC4    : -------------------------------------
Bra003001  : -------------------------------------
BoCNGC16   : -------------------------------------
Bra008699  : -------------------------------------
BoCNGC17   : -------------------------------------
AtCNGC2    : -------------------------------------
Bra004537  : -------------------------------------
BoCNGC2    : -------------------------------------
AtCNGC3    : -------------------------------------
Bra000937  : -------------------------------------
BoCNGC1    : -------------------------------------
AtCNGC13   : ALRNLRANAAARNSRFPHMLTLLPQKPADPEFPMDET
AtCNGC10   : -------------------------------------
Bra034281  : -------------------------------------
BoCNGC3    : -------------------------------------
AtCNGC11   : -------------------------------------
AtCNGC12   : -------------------------------------
Bra003323  : -------------------------------------
Bra031515  : -------------------------------------
Bra022632  : -------------------------------------
AtCNGC1    : -------------------------------------
Bra003081  : RSL----------------------------------
Bra020402  : -------------------------------------
BoCNGC7    : -------------------------------------
AtCNGC5    : -------------------------------------
Bra024067  : -------------------------------------
BoCNGC6    : -------------------------------------
AtCNGC9    : -------------------------------------
Bra039221  : -------------------------------------
BoCNGC4    : -------------------------------------
Bra032132  : -------------------------------------
BoCNGC5    : -------------------------------------
AtCNGC6    : -------------------------------------
Bra026086  : -------------------------------------
BoCNGC8    : -------------------------------------
AtCNGC7    : -------------------------------------
AtCNGC8    : -------------------------------------
Bra008733  : -------------------------------------
BoCNGC13   : -------------------------------------
AtCNGC18   : -------------------------------------
Bra018089  : -------------------------------------
BoCNGC14   : -------------------------------------
AtCNGC16   : -------------------------------------
Bra011186  : -------------------------------------
BoCNGC10   : -------------------------------------
AtCNGC17   : -------------------------------------
Bra007839  : -------------------------------------
BoCNGC12   : -------------------------------------
Bra032081  : -------------------------------------
BoCNGC11   : -------------------------------------
AtCNGC14   : -------------------------------------
Bra011963  : -------------------------------------
BoCNGC9    : -------------------------------------
AtCNGC15   : -------------------------------------
Bra021266  : -------------------------------------
BoCNGC21   : -------------------------------------
BoCNGC20   : -------------------------------------
AtCNGC20   : -------------------------------------
BoCNGC22   : -------------------------------------
Bra029958  : -------------------------------------
BoCNGC23   : -------------------------------------
Bra021265  : -------------------------------------
BoCNGC24   : -------------------------------------
AtCNGC19   : -------------------------------------
Bra022232  : -------------------------------------
BoCNGC25   : -------------------------------------
Bra022233  : -------------------------------------
BoCNGC26   : -------------------------------------
Bra001676  : -------------------------------------
BoCNGC18   : -------------------------------------
Bra001678  : -------------------------------------
BoCNGC19   : -------------------------------------
Bra031529  : -------------------------------------
```
