# Supplementary figures and images for: Comprehensive genomic analysis of the CNGC gene family in Brassica oleracea: novel insights into synteny, structures, and transcript profiles
Source: BMC Genomics. 2017 Nov 13;18:869. doi: 10.1186/s12864-017-4244-y (PMC5683364; doi:10.1186/s12864-017-4244-y)

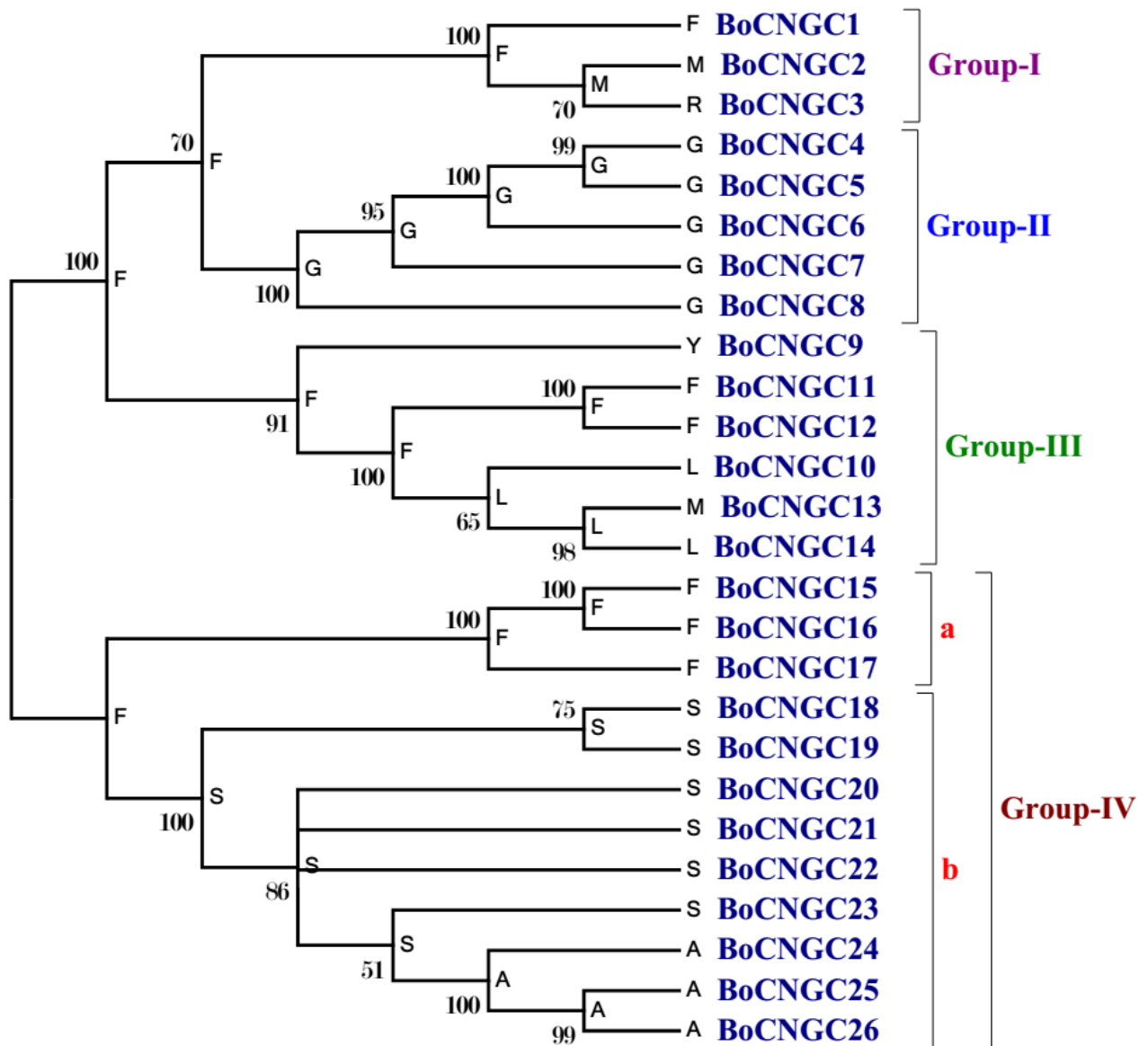

Supplement: Supplementary file 3 — Phylogenetic tree of CNGC proteins from B. oleracea (encoded by BoCNGCs). A multiple sequence alignment was performed using ClustalX 2.0 program with default settings. Maximum likelihood (ML) tree was create with MEGA 6.0, under the Jones-Taylor-Thornton (JTT) model. The bootstrap values from 1000 resampling are given at each node. (PDF 201 kb) [file 12864_2017_4244_MOESM3_ESM.pdf]

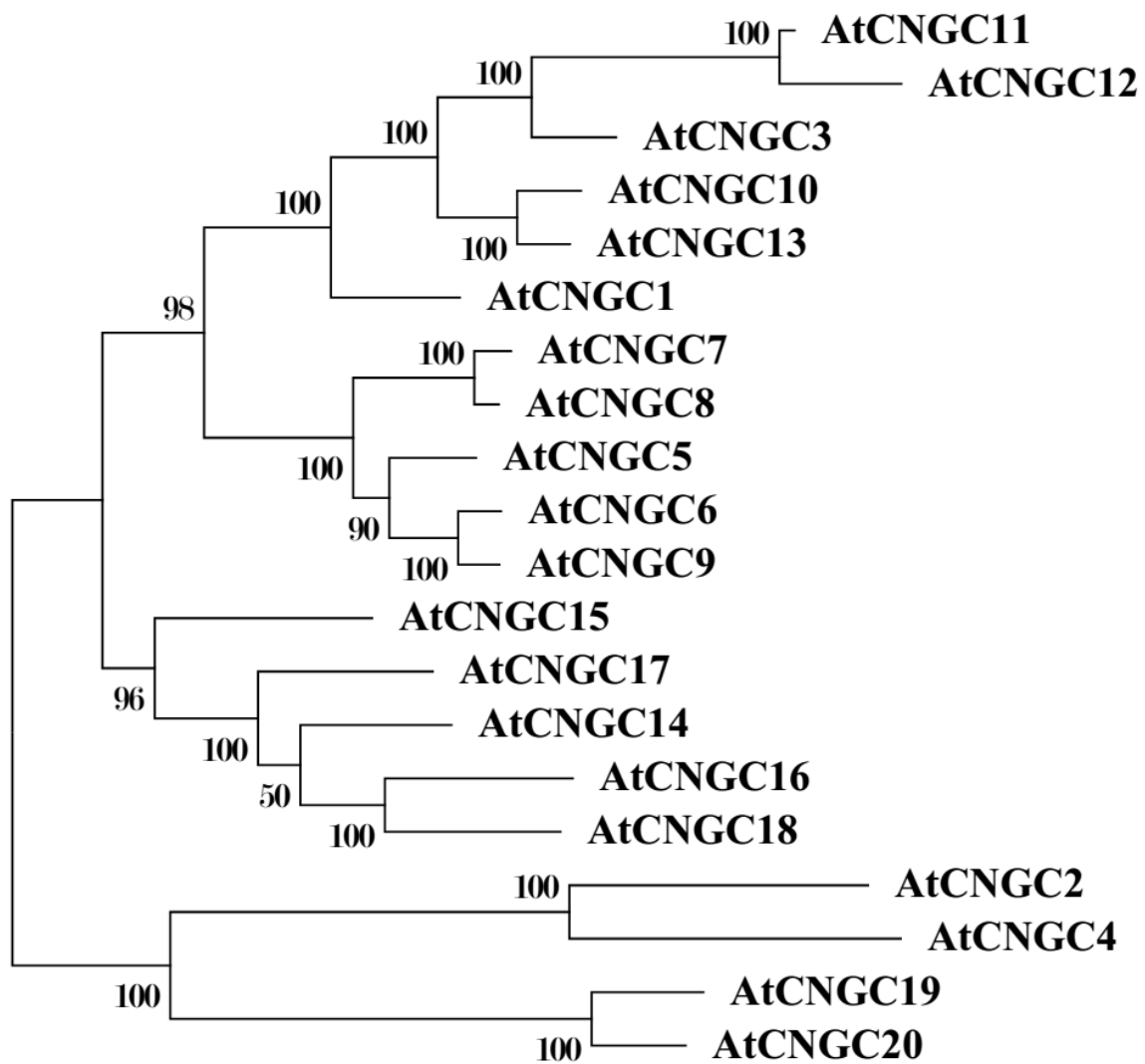

Supplement: Supplementary file 4 — Phylogenetic tree of CNGC genes from Arabidopsis (AtCNGCs). A multiple sequence alignment was performed using ClustalX 2.0 program with default settings. Maximum likelihood (ML) tree was create with MEGA 6.0, under the Jones-Taylor-Thornton (JTT) model. The bootstrap values from 1000 resampling are given at each node. (PDF 180 kb) [file 12864_2017_4244_MOESM4_ESM.pdf]

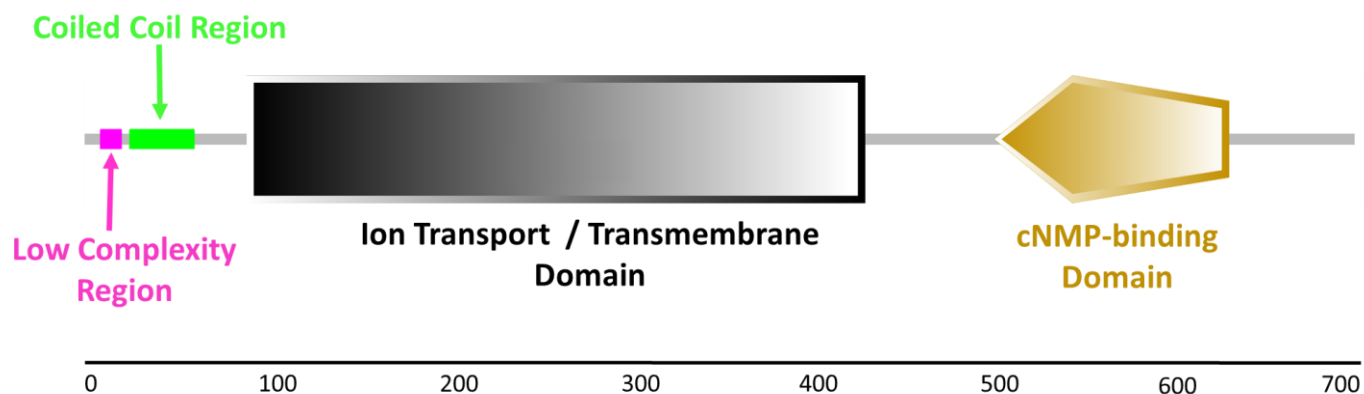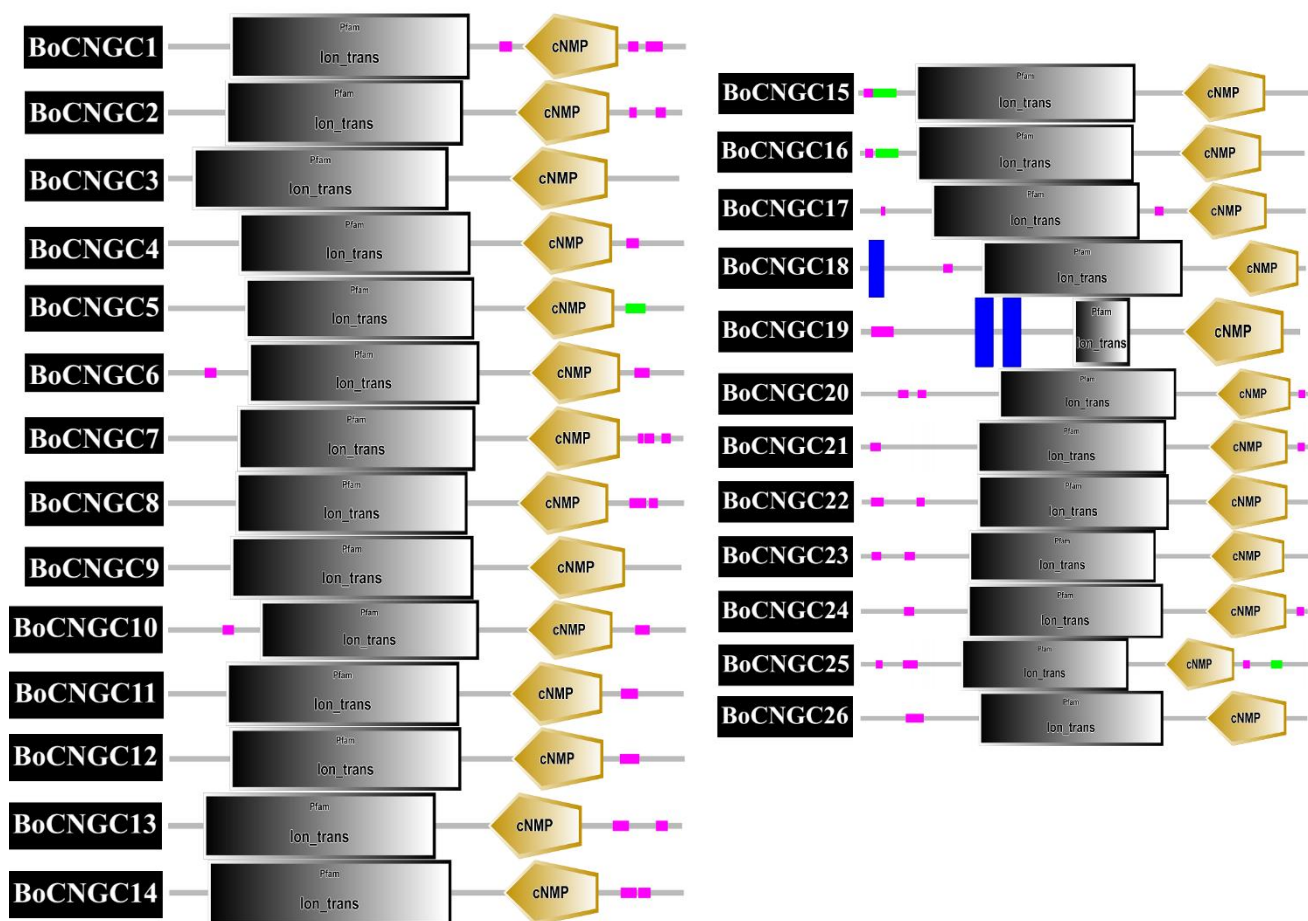

Supplement: Supplementary file 7 — Primary domain architecture of BoCNGC proteins. Information about domain annotation is obtained from SMART database. (PDF 339 kb) [file 12864_2017_4244_MOESM7_ESM.pdf]

## Motif 1

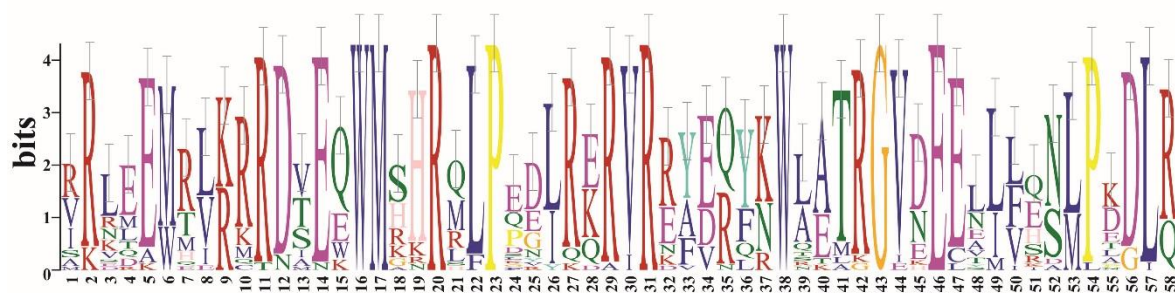

## Motif 2

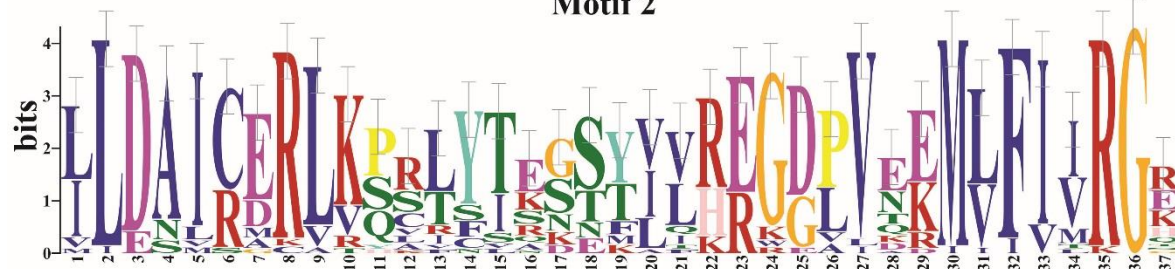

### Motif 3

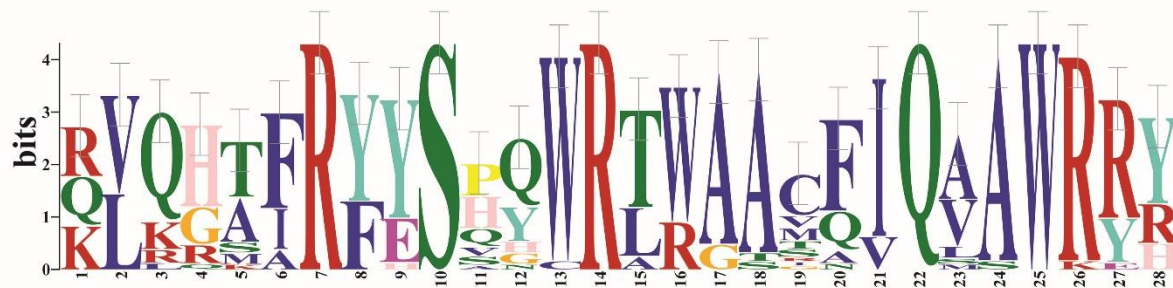

## Motif 5

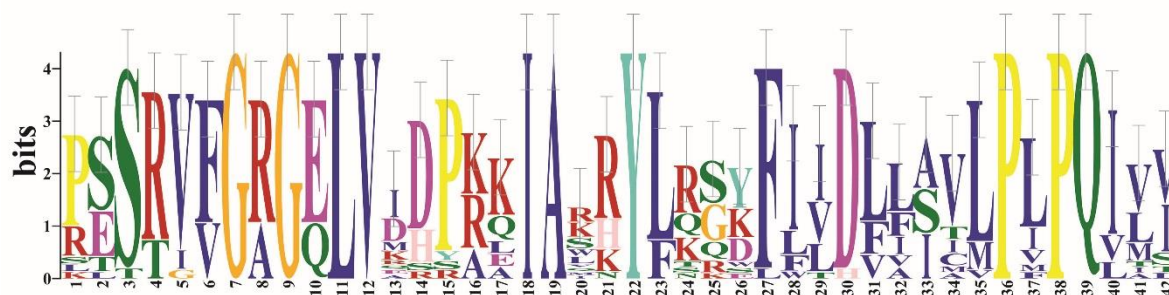

Supplement: Supplementary file 13 — Web logos of MEME-identified conserved functional motifs in BoCNGC proteins. The heights of the amino acids indicates the degree of conservation. (PDF 423 kb) [file 12864_2017_4244_MOESM13_ESM.pdf]

# PLANT-PATHOGEN INTERACTION

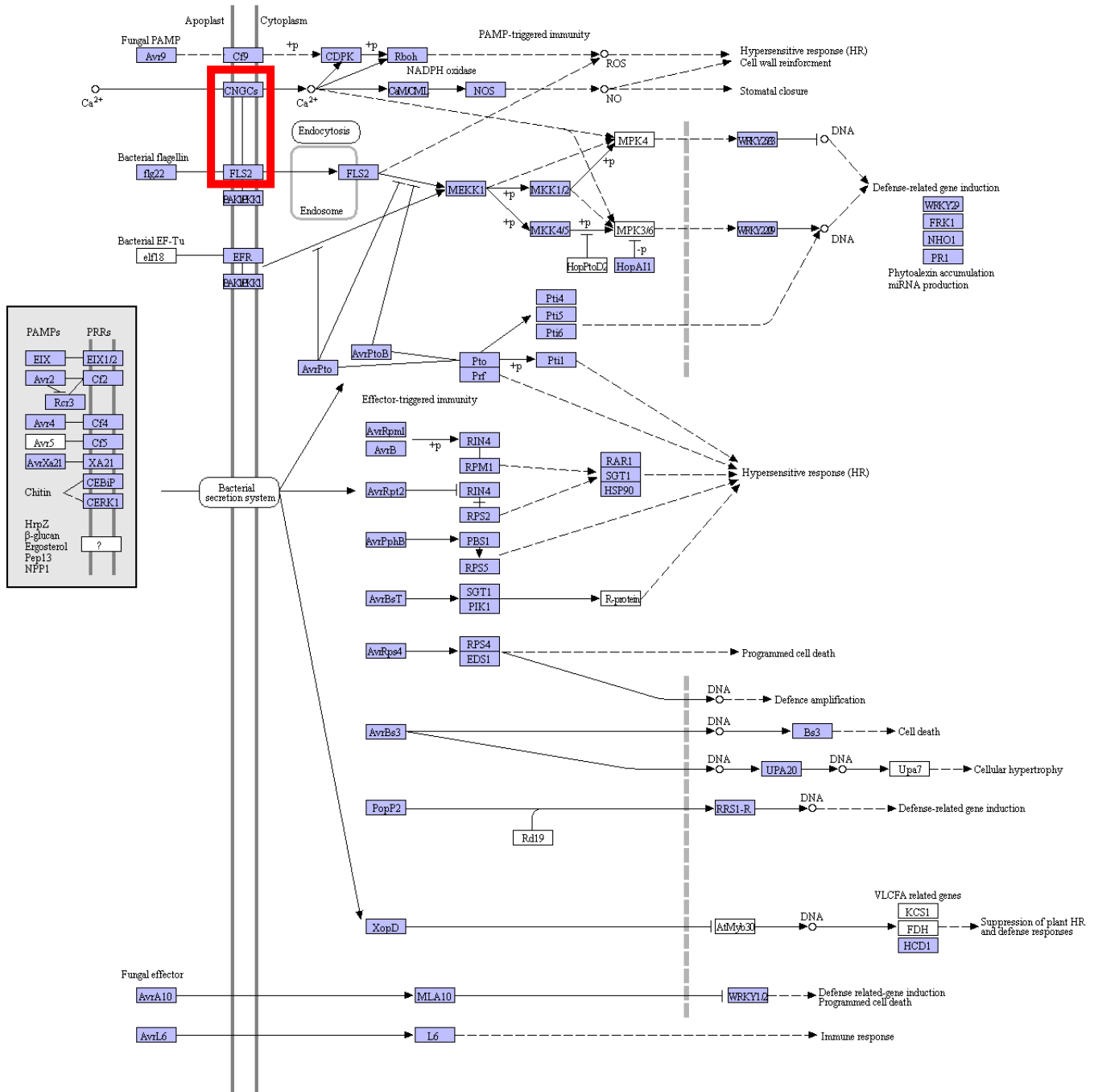

Supplement: Supplementary file 14 — KO pathway associated with plant-pathogen interaction (K05391). The pathway map was obtained from http://www.kegg.jp/kegg/kegg1.html. Details of BoCNGC genes allocated to K05391 pathway are given in Additional file 20. (PDF 126 kb) [file 12864_2017_4244_MOESM14_ESM.pdf]

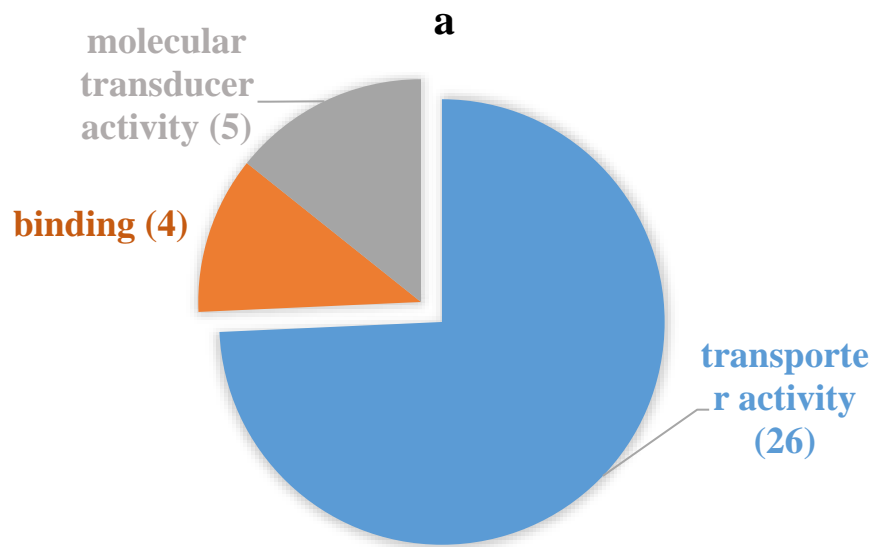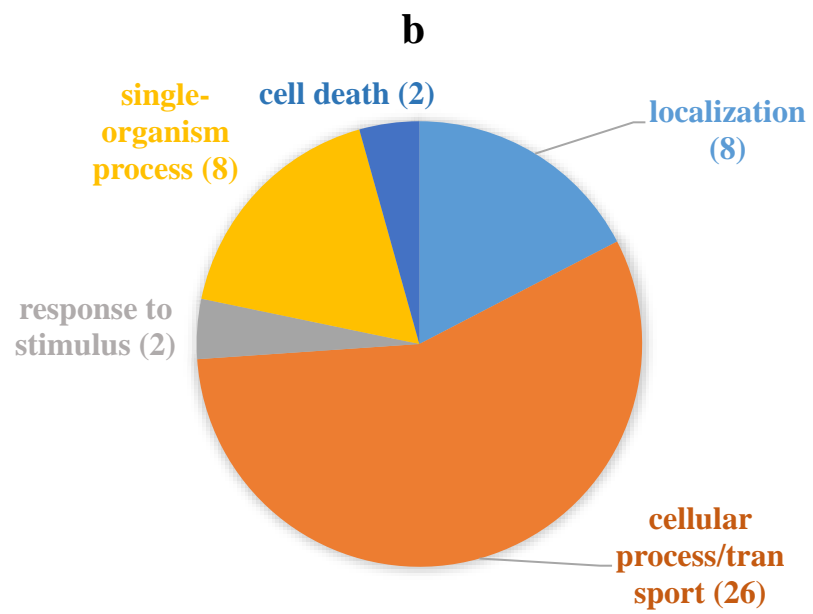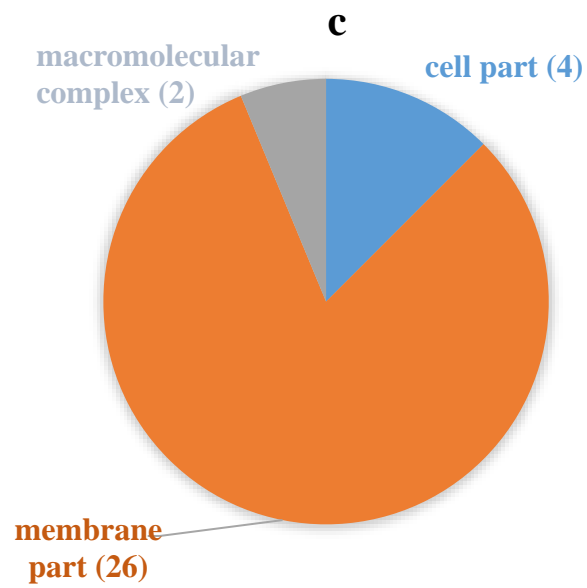

Supplement: Supplementary file 18 — Distribution of BoCNGC genes in major functional terms (GO terms Level 2) for categories Molecular Function (a), Biological Process (b) and Cellular Component (c). The details are given in Additional file 19. (PDF 97 kb) [file 12864_2017_4244_MOESM18_ESM.pdf]

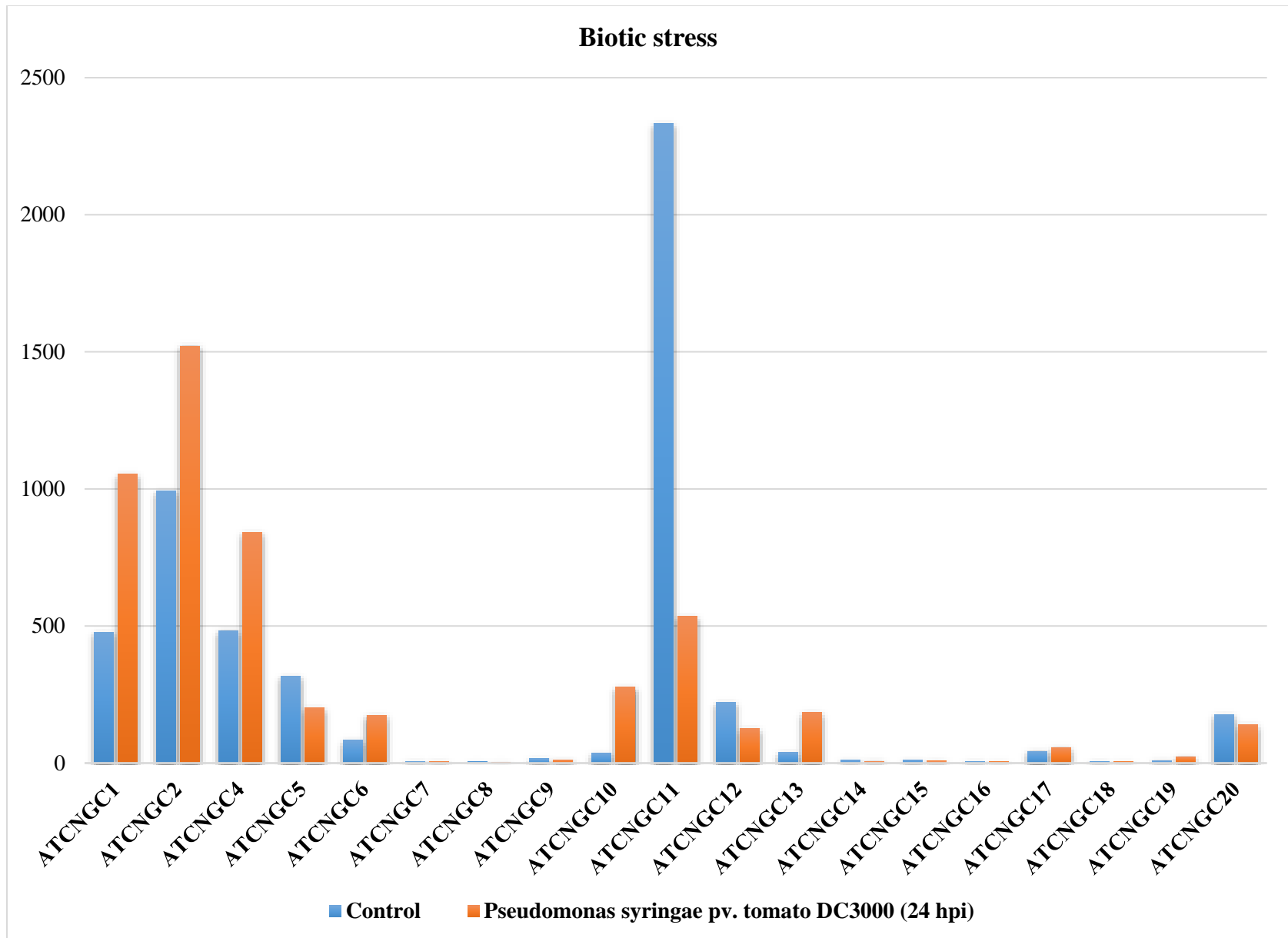

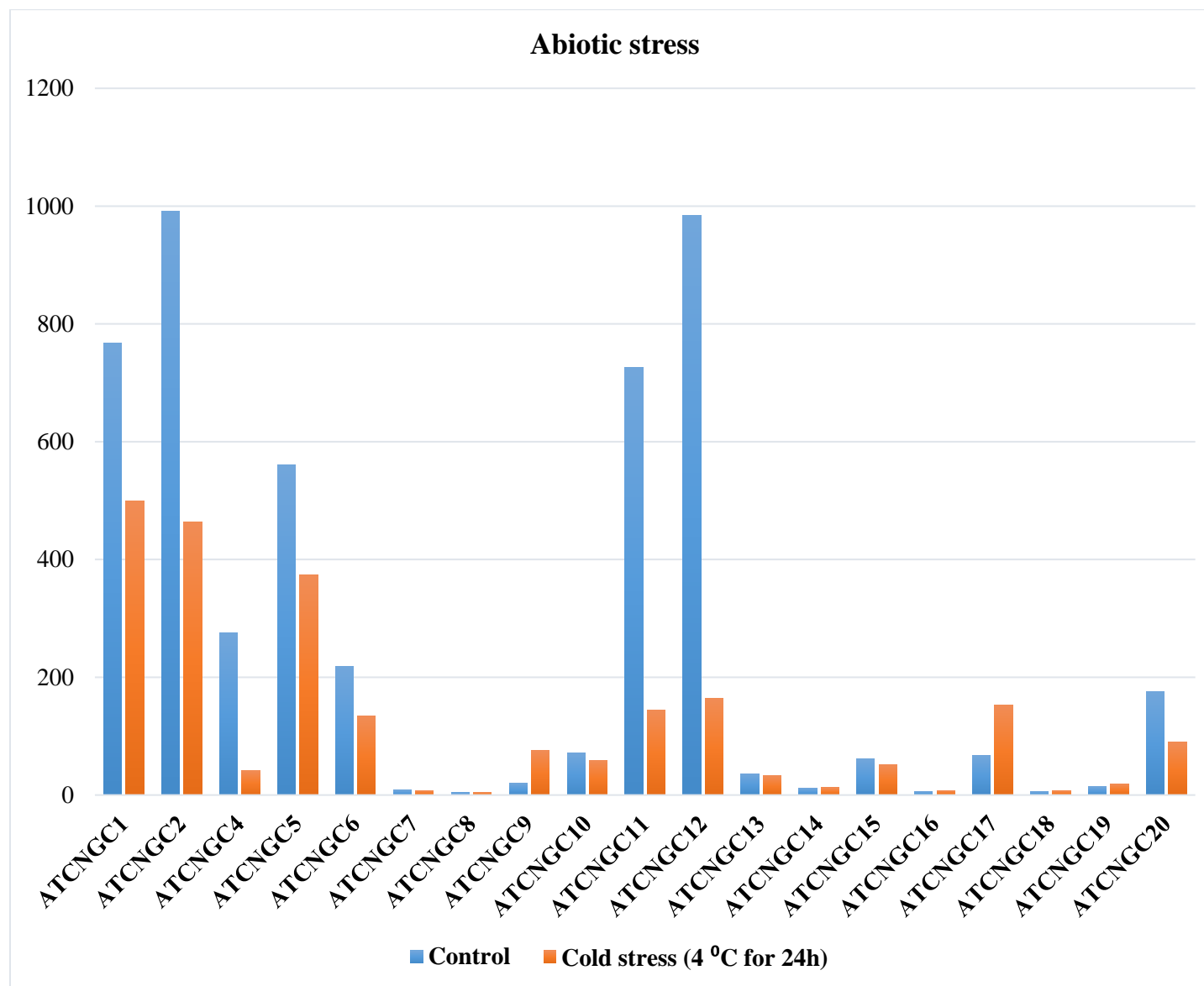

Supplement: Supplementary file 22 — Cumulative values of expression for Arabidopsis CNGC genes in response to pathogen (biotic) and cold (Abiotic) stress. The expression data for 21 days old of wild type and mutant plants was obtained from Schmid et al. [48]. (PDF 294 kb) [file 12864_2017_4244_MOESM22_ESM.pdf]
