# Supplementary material for: Comprehensive genomic analysis of the CNGC gene family in Brassica oleracea: novel insights into synteny, structures, and transcript profiles
Source: BMC Genomics. 2017 Nov 13;18:869. doi: 10.1186/s12864-017-4244-y (PMC5683364; doi:10.1186/s12864-017-4244-y)
Supplement: Supplementary file 8 — Multiple sequence alignment of BoCNGC proteins. Multiple sequence alignment was performed by clustalX2and viewed by GeneDoc software package. (PDF 767 kb) [file 12864_2017_4244_MOESM8_ESM.pdf]

Additional file 8. Multiple sequence alignment of BoCNGC proteins. Multiple sequence alignment was performed by clustalX2and viewed by GeneDoc software package.

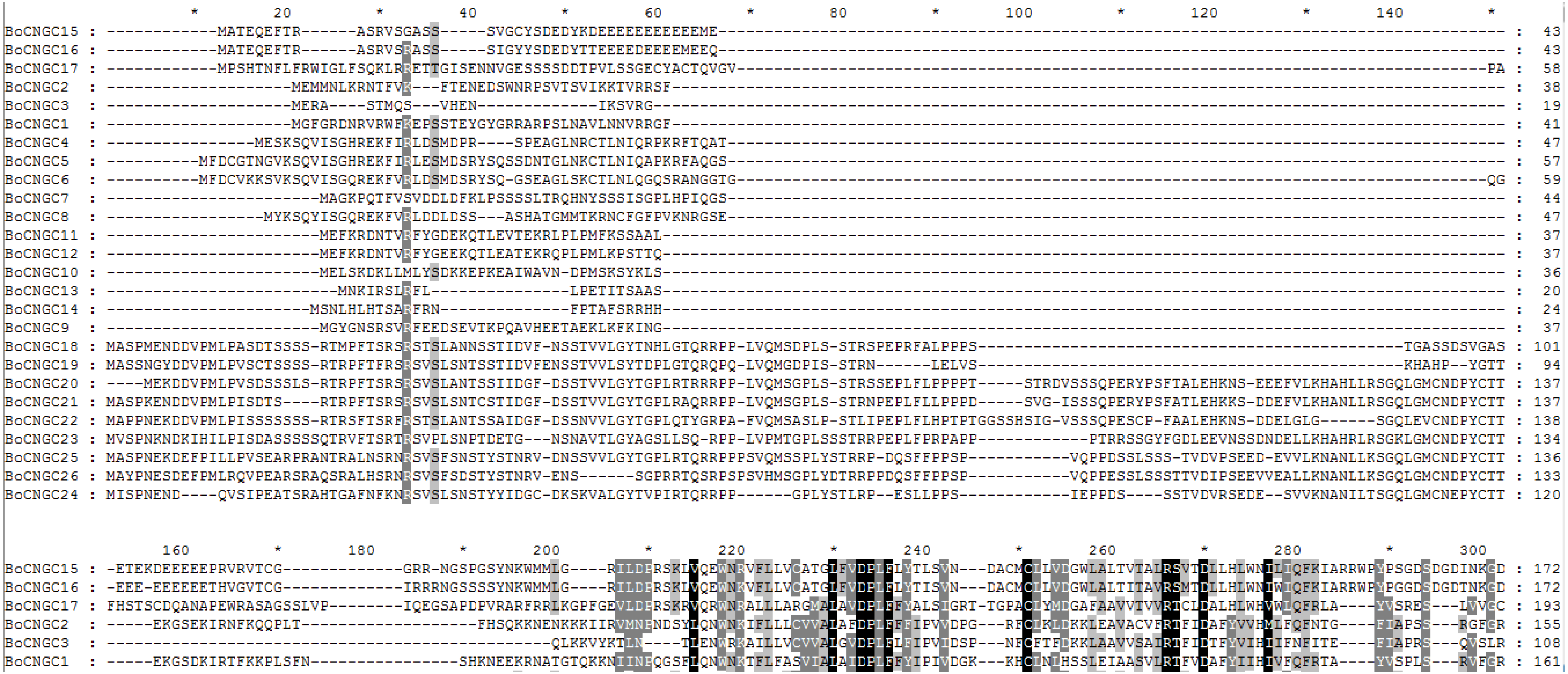

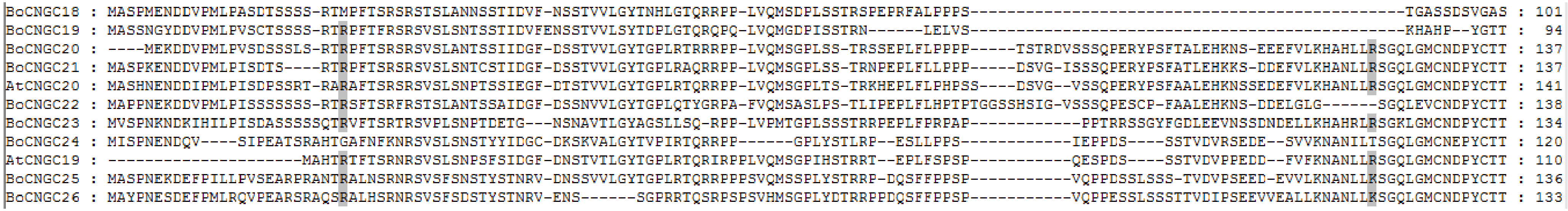

```
BoCNGC4  : ---KASSGSFKKGFRKGSEGLWSI--------GRSIGLGVSRAVFPEDLEVSEKKIFDPQDKFLLLCNKLFVASCILAVSVDPLFLFLLPFINDK---AKCVGIDRKLAIVTTTLRTVIDSFYLFHMALRFRTA----YVAPSS---RVFGR : 177
BoCNGC5  : ---KTSSGSFKNGFRKGSEGLWSI--------GRSIGLGVSRAVFPEDLKVSEKKIFDPQDKFLLLCNKLFVASCILAVSVDPLFLYLPFINDK---AKCIGIDRKLAIVATTLRTVIDSFYLFHMALRFRTA----YVAPSS---RVFGR : 187
BoCNGC6  : NNNNASSGSFKKGFRRGSKGLWSL--------GRSIGL-VSRAVFPEDLKVSEKKIFDPQDKFLLLCNKLFVTSCILAVSVDPLFLYLPFINDS---GKCIGIDRRLATIATTLRTFIDVFYLFRMALQFRTA----FVAPSS---RVFGR : 191
BoCNGC7  : ---HNASGSFKKRFQKGSKGLKSI--------GRSLGFGVYRAVFPEDLKVSEKKIFDPQDKTLLFCNKLFVVSCILSVFVDPFFFYLPVINGE---SKCIGIDRKLAITATTFRTFIDVFYLAHMALQLRTA----YIAPSS---RVFGR : 174
BoCNGC8  : --KKRASKSFREGVKIGSEGLFSI--------GK----SVTRAVFPEDLRISEKKIFDPQDKTLLIWNRMLVISCILAVSVDPLFFYLPIVDNSG--SSCIGIDTKLAVTTTTLRTILDVFYLTRMALQFRTA----YIAPSS---RVFGR : 175
BoCNGC11 : -----FQKQELGTSKKS----I-----------FKIPRFGRFKVFPENFEIERDKILDPGGDVVLQWNRVFLFWCLVALYVDPLFFFLYSVKRT-GRSSCMTTDLNLGIVVTFFRTLADLFYVLHIVIKFRTA----YVSRTS---RVFGR : 160
BoCNGC12 : -----FLKPELVIPKKTNKTRL-----------FKLPRFGGLKVFPENFEIERDKILDPGGDVVLQWNRVFLFWCLVALYVDPLFFFLSSVKNT-GRSSCMTTDLKLGIVVTFFRTLADLFYVLHIVIKFRTA----YVSRTS---RVFGR : 164
BoCNGC10 : -----LPSALKPPPDNLLAG-------------NRISRYTDNNKTKSSKLSWYKTILDPGSEIVLKWNWVFIISCMIALFIDPLYFFVPAIGGN-KDYPCAKTDTNLRILVTFFRTIADLFYLLHIFIKFRTG----FIAPNSS-TRVFGR : 163
BoCNGC13 : -----NRGSVA-----------------------------VRYGSQVLPWR-HQILDPDSSIVTYWNHVFLVTSIIALFLDPFYFYAPYVG----GPACLSVDVGLAATVTFFRSVADLFHLLHIFMKFRTA----FVARSS---RVFGR : 124
BoCNGC14 : -----NNNDLQ-----------------------------NQRGRSVFSELGDTTLDPSGDLITRWNHIFLITCLIALFLDPLYFYLPIVQA---GTACMSIDIGFGIIVTFFRTLADFSFLIHILLKFKTA----FVSKSS---RVFGR : 130
BoCNGC9  : -----AQISPRKNVKKMTRG-------------KSFKAKVLSRVFTEDLGRVKNKILDPRGQTIRRWNKLFLIACLVSLFVDPLFFFLPVMR----KEACITIGIRLEVVLTVIRSLADAFYIAQIVIRFRTA----YIAPSS---RVFGR : 159
BoCNGC18 : SSQPNERNHAYSRTPRVFATSDF-----TLHNALDDDAKGWA--------KYFSGIIYPESNFVQIWTTFFALSCLCSIFVDPLFFYPIEIYK---EERCIKIDWWTTNVFVIVRSITDGLYALNIVLQFR----LAYVDLES---TVVGA : 229
BoCNGC19 : CPSDYNREAAQIPTPRV---SDS-----MVHNARDDDAKGWARRFATSVDKYLPKIMEPNSKQVRGWTIFFAFSCLLSIFVDPLFFFPIEVSQ---SGKCIRIDEDMAIVLVALRSLTDVLYFVNILLQR-------------------- : 213
BoCNGC20 : CPSYYNRKAAQIPTSRVSAIFDS-----TFH-----DAKGWARRFATSINRHLPGIMNPHSKFVQSWTKFFAFSCLLAIFIDPLFFFLILVKQ---NNNCIEIDWPMAHTFVAVRSVTDVLFSVNILIQ----FRLAYVAPES---TVVGA : 268
BoCNGC21 : CPSYYNRKAAQIPSSRVSAFFDS-----KFHNALYDDAKGWARRFATTANRYLPGIMNPHSKFVQSWTKFFALSCLLAIFIDPLFFFLIFVKQ---NDKCIMIDWPMAKAFVAVRSVTDILFSVNILIQ----FRLAYVAPES---TVVGA : 273
BoCNGC22 : CPSYYNPKPAQIPPS-------------KLHHAMCDDARGWATRFVTSINKFLTGIINPHYKFVQRWTKLFAS--WIVIFIDPLFLFLIKVKE---NDKCIMIDWPVAKAFIAVRSVTDVLFFVNIMIQ----FRLAYATLES---TVVGV : 264
BoCNGC23 : CPSNYNPKASRLPNPTVS---AS-----TFHNALYDDARSWARRFASSVNRCLPGIMNPHSKVVQIWTKFFAVSSLIAIFIDPLFFFIILVQK---NNKCVVIDWPIATAFVIFRTLTDVIFFANMLIQAPVPFRLAYVARES---TVVGA : 271
BoCNGC25 : CPSYYNRQAAQFHTNRVVSDSR-------FRTALYDDARGWAKRFASSVRKLIPGIMNPHSKFVQMWTRFLAFSCLVAIFIDPAFLFLLSIRQ---DNKCIEIDWPKTKVFVSLRTMSDLIFFINILIQ----FRLAYVAPES---RIVGA : 270
BoCNGC26 : CPSYYNLQAAQFHTYGVVSDSRTQVNATHNRQALHDYDRGWDKLFASYVRRCVPGIINPHSKFVQMWTRFLAFSCLLSIFIDPAF-----------DNKCTDIDWPKATIFAFLRSMSDFIFFINILIQ----FRLAYVAPES---RIVGA : 266
BoCNGC24 : CPSYYSHQSANFHTS-KVSDSR-------FHTALYDDARGWAKRFASSVRRCVPGIMNPHSKFVQVWTRFLAFSCLMAIFIDPTFVFLLLIRH---DNKCIEIDWTKTTVLVSLRSMFDLIFFINILIQ----FRMAYVAPES---RIVGA : 253
                                                                p    6  w      c   6  DP15                C    d           R3  D      6                 s     g

                 *       320         *       340         *       360         *       380         *       400         *       420         *       440         *
BoCNGC15 : GTRVRT--RVAPPYVKKNG-FFFDLFVILPLPQVVIWVVIPSLLKRGSVTLVVSILLLTFLFQYLPKIYHSIRHLRRNATLSGYIFGTVWWGIALNMVAYFVAAHAAGACWYLLGVQRSAKCLKEQCENTMG------CDLRMLSCKEPVY : 314
BoCNGC16 : ETRLRTSRRVAPPYVKKKGTFFFDLFVILPLPQVVIWVVIPSLLKRGSVTLVVSVLLVTFLFQYLPKIYHSVRHLRQNATLSGYIFGTVWWGIALNMIAYFVAAHAAGACWYLLGVQRSAKCLKEQCESTMG------CDLRMLSCKEPVY : 317
BoCNGC17 : GKLVWDPRAIASHYARSLTGEWFDVIVILPVPQAVFWLVVPKLIREEKVKLIMTILLLIFLFQFLPKIYHCICIMRRMQKVTGYIFGTIWWGFALNLIAYFIASHVAGGCWYVLAIQRVASCIRQQCMRTAN------CNL-SLSCQEEVC : 337
BoCNGC2  : GELVQSYKKIAVRYL--KSYFIIDVLSILPIPQVVVLAVVPSMGRPASLVT-KELLKWVIFCQYVPRIARIYPLFKEVTRTSGLVTETAWAGAALNLFLYMLASHVFGSFWYLISIERKDRCWRETCAKIK------GCIHAYLYCSGG-E : 296
BoCNGC3  : GELIVHSKATRKRLF--FFHFIVDICSVIPIPQVVVLILIH---RSDSLVS-QAILKWIILTQYVPRIIRIYPLLKEVTRASGTIAETKWVGAAFNLFLYMLHSHVFGAFWYVSSVEKKNKCWRLECAKIF------GCNLRYQYCARGRQ : 247
BoCNGC1  : GELVEDPKAIALKYL--SSYFIIDVLSILPLPQLVVLAVIPNVEKPVSLLT-KDYLITVIFAQYIPRILRIYPLYSEVTRTSGIVTETAWAGAAWNLSLYMLASHVFGALWYLISVEREDRCWREACEKRQ------GCELRFLYCDGNNN : 303
BoCNGC4  : GELVIDPAQIAKRYL--QQYFIIDLLSVLPVPQIIVWRFLY-SSRGANVLATKQALRYIVLVQYIPRFLRMYPLSSELKRTAGVFAETAWAGAAYYLLLYMLASHIVGALWYLLALERNNDCWSKACKDN------DNCTRNFLFCGNQNM : 319
BoCNGC5  : GELVIDPKQIAKRYL--QQYFIIDLLSVLPLPQITVWRFLY-TSKGANVLATKQALRYIVLVQYIPRFLRMYPLSSELKRTAGVFAETAWAGAAYYLLLYMLASHIVGALWYLLALERNNDCWSKACVKK------DNCTRNFLFCGNQNM : 329
BoCNGC6  : GELVIDPAQIAKRYL--QQYFIVDFLSVLPLPQIVVWRFLY-TSTGGSVLETKQALRSIILVQYIPRFFRIYPLSKELKRTAGVFAETAWAGAAYYLLLYMLASHIVGALWYLLALERVNGCWKRACLLDG-----QNCTRNYLFCGNENM : 334
BoCNGC7  : GELVIDPAQIAKRYL--QRWFIIDFLSVLPVPQIVVWRFLQ-RSRGSDVLATKQALLFIVLVQYIPRFLRVLPLTSELKRTAGVFAETAWAGAAYYLMLYMLASHIVGAFWYLLALERNDACWQEACSDAG----KKICTTGFLYCGNQNM : 318
BoCNGC8  : GELVIDPAKIAQRYL--TRYFIVDFLAVLPLPQIAVWKFLH-GSKGMDVLPTKTALLNIVITQYIPRFVRFIPLTSELKKTAGAFAEGAWAGAAYYLLWYMLASHITGAFWYMLSVERNDTCWRFACKVQP----DPKLCVQILYCGTKFV : 319
BoCNGC11 : GELVKDPKLIARRYL--RSDFIVDLIACLPLPQIVSWFILP-SIRSSHSDHTTNAIVLIVLVQYIPRLYLIFPLSAEIIKATGVVTTTAWAGAAYNLLQYMLASHILGAAWYLLSIQRQATCWKAECHKEF---APLECVTDFFDCG-TLH : 304
BoCNGC12 : GELVKDPKLIARRYL--RSDLIVDLIACLPLPQIVSWFILP-SIRSSHSDHTTNAIVLIVLVQYIPRLYLIFPLSAEIIKATGVVTTTAWAGAAYNLLQYMLASHILGAAWYLLSIERQATCWKAECHNEL---GPIRCVTDFFDCG-TVN : 308
BoCNGC10 : GELVMDPKAIAWRYL--KSDFIIDIVATLPLPQIVIWFVMP-ATRSYRFDHSNNDIALIVLLQYIPRFYLIIPLSSQIVKATGVVTKTAWAGAAYNLLLYMLASHVLGAAWYILSFDRYTSCWKTRCNKEH---GGVNCYLYYLDCDSPLY : 308
BoCNGC13 : GELVRDPREIAMKYL--KSDFIVDVAAMLPLPQLVIWLVIP-AATNGTANHANSTLALIVLVQYIPRSFIIFPLNQRIIKTTGFIAKTAWAGAAYNLLLYILASHVLGAMWYLSSIGRQFSCWSKVCEKDH-ALRVLDCLPSFLDCK-SLQ : 270
BoCNGC14 : GELVMDRREIAIRYL--KSEFIIDIAATLPLPQIMIWFVIPNAGEFRYAAHQNHTLSLVVLIQYVPRILVMLPLNRRIIKATGVAAKTAWSGAAYNLVLYLLVSHVLGSVWYVLSIQRQHECWRRECIKEMNATHSPSCNLLFLDCG-SLR : 278
BoCNGC9  : GELVIDSRKIAWRYL--NKSFWIHIVAALPLPQVLIWIVIP-NLRGSPMTNTKNTLRFIILFQYVPRMFLIFPLSRQIIKATGVVTETAWAGAAYNLMLYMLASHVLGACWYLLAVERQEACWRHACNIEK-----TICQYRFFECR-RLE : 301
BoCNGC18 : GQLVDDPKKIASHYLR--GKFLTDFFIVLPIPQILILWIIPQLLGTSGANNTKNYLRAAILVQYIPKLRRLFPLLAGQT-PRGFRFDSALAKFFINLLTFMLAGHVIGSCWYILGLQRVNQCLRDAC-GNSS-----FECKQLIDCGRENR : 371
BoCNGC19 : ----------VNRCLR-------------------------------------------------------------------ETCEDHMELKCHGVCKELIDCGLHLK--------------------------EKLIDCERRNT : 256
BoCNGC20 : GQLVDHPRKIASHYFR--GKFLLDLFIVMPLPQILILWIIPAHLAISGANYAKNLLRAAVLFQYIPKLYRLLPLLAGQT-PTGFIFESAWANFFINLLTFMLAGHVVGSCWYIFGLQRVNQCLRDAC-GNTD-----RPCRELLDC-HGET : 409
BoCNGC21 : GQLVAHPRKIARHYFR--GKFLLDLFIVMPLPQILILWIIPAHLGASGANYAKNLLRAAVLFQYIPKLYRLLPLLAGQT-PTGFIFESAWANFVINLLTFMLAGHVVGSCWYIFGLQRVNQCLRDAC-GNSD-----HECRNLIDCGRGDS : 415
BoCNGC22 : GHLFDHPRKIARNYLR--VKFLLDLFIVMPLPQIWIFWILPVQLGASGDNYAKNFLTAAVLFQYIPKLCRLLPLLAGKT-PTVFLFESACS-FVTNLLTFMLVGHVVGSCWYIFGLQRVNHCLRDAC-GNSH-----QECRELIYCGHGNS : 405
BoCNGC23 : GQLVDRPKKIALHYFR--GYFIVDLIIVMPFPQVLVLSVIPAQLAISGTNYAKNLLRAGILVQYIPKLYRLLPLLAGQT-PTGLIFESAWANFVINLLTFMLAGHVVGSCWYIFGLQRVNQCLRNAC-GHSG-----RECRGLIDCGHGNS : 413
BoCNGC25 : GQLVDHPRKIASKYFR--GKFFLDLLIVVPMPQIMILSMLPAHLGTPRSELEKNIIRIVFLFQYIPKLYRLLPLLAGQT-PTGFIFESAWANFIINLLTFILAGHAIGSFWYIAGLQRVKKCLLHAG-NYTM-----DERRNLIDCARGVN : 412
BoCNGC26 : GQLVDHPRKIACNYLR--GKFFLDLLIVYPVPQIMIFSILP------KSEFEENTVIALFLFQYIPKLYRLLPLIAGQT-PTGFIFESAWSNFIINLLTFILAGHAVGSFWYIAGLQRVKKCLLQAG-NFTM-----DERRYIIDCTSGDS : 402
BoCNGC24 : GQLVDHPRKIARNYLR--GKFFLDLFIVLPIPQIMTLSILPAHLGTSTAGFERNITRSIFIVQYIPKLYRLLPLLAGQT-PTGFIFESAWASFVINLLTFMLAGHAVGSCWYFSGIERVKKCLLYAAWNNSV-----DERRNLIDCARGNM : 396
           g l       a  y      f  d      p pq                                           q  p      pl        g      aw       6     6a h  g  wy          c    c                  C

                  460         *       480         *       500         *       520         *       540         *       560         *       580         *       600
BoCNGC15 : YGTTEMVLDRARLAWARNHQ---ARSVCLDSDTNYTYGAYQWTIQLVSNES----RLEKILFPIFWGLMTLSTFGN-LESTTEWSEVVFNIIVLTSGLLLVTMLIGNIKVFLHATTS---------------------KKQAMHLKMRN : 434
BoCNGC16 : YGTTEMVLDRARLAWAQNNQ---ARSICLDINTNYTYGAYKWTIQLVSNES----RLEKILFPIFWGLMTLSTFGN-LESTTEWSEVVFNIIVLTSGLLLVTMLIGNIKVFLHATTS---------------------KKQAMHLKMRN : 437
BoCNGC17 : YQFVSPTSTIGFPCLSGNLTSVVKKPMCLDSNGPFRYGIYGWALPVISSNS----LAVKILYPIFWGLMTLSTFGNDLEPTSNWLEVVFSIVMVLSGLLLFTLLIGNIQVFLHAVMA---------------------KKRKMQIRCRD : 461
BoCNGC2  : DNSQYLIGSCPLMDPEEIKNST----------VFNFGIFAEALQSGVVES--MDFPKKFFYCFWWGLRNLSALGQNLKTSAFEGEIIFAVIICISGLVLFALLIGNMQKYLQSTTV---------------------RVEEMRVKRRD : 411
```

```
BoCNGC3  : NNGRYLNTTCPLIDPDQIIGST-----------VFNFGMYTDALRSGIVESKPRDFPRKFFYCFWWGLRNISALGQNLKTSNSVGDIVFALIICVSGLLLFAVLIGNIQKYLQSTTI-----------------------RLDEMEEKKRD : 364
BoCNGC1  : VINDYLTTSCPFINPDDITNST-----------TFNFGIFTDALKSGIVES--DDFWKKFFYCFWWGLRNLSALGQNLNTSKFVGEIIFAVLIICISGLVLFALLIGNMQKYLESTTV-----------------------REEEMRVRKRD : 418
BoCNGC4  : KG-YDAWDDVKDPFLQLRCPVNVTDGE----EPPFDFGIYLRALSSGIVSS--KKFVSKYFFCLWWGLQNLSTLGQGLETSTYPGEVIFSIVLAIAGLLLFALLIGNMQTYLQSLTI-----------------------RLEEMRVKRRD : 440
BoCNGC5  : EG-YAAWYTAKSSVLQEKCPVNVTEGE----EPPFDFGIYSRALSSGIVSS--KKFVSKYFFCLWWGLQNLSTLGQGLETSTYPGEVIFSIALAIAGLLLFALLIGNMQTYLQSLTI-----------------------RLEEMRVKRRD : 450
BoCNGC6  : DG-YAAWNTIKESVLQKSCPVNVTDGD----NPPFDFGIYLRALSSGIVSS--ESFVAKYFFCLWWGLQNLSTLGQGLETSTYPGEIIFSIALAVAGLLLFALLIGNMQTYLQSLTI-----------------------RLEEMRVKRRD : 455
BoCNGC7  : DG-YDVWNKTKESVLQSKCRAELDD-P----NPPFDFGIYTQALSSGIVSS--QKFITKYCYCLWWGLQNLSTLGQGLETSTYPLEIMFSITLAISGLILFALLIGNMQTYLQSLTI-----------------------RLEEMRVKRRD : 438
BoCNGC8  : SSRETEWIKTVPELLKSNCSAKADD-------AKFNYGIYGQAISSGIVSS--TTFFSKFCYCLWWGLQNLSTLGQGLQTSTFPGEVLFSIAIAIAGLLLFALLIGNMQTYLQSLTV-----------------------RLEEMRIKRRD : 438
BoCNGC11 : RPDRNNWQNITV--VFSNCDPSND--------IKFTFGIFADALTKNVVSS--P-FLEKYLYCLWFGLQNLSSYGQNLDTSTSVLETMFAILVAIFGLVLFALLIGNMQTYLQSITV-----------------------RLEEWRLKRRD : 419
BoCNGC12 : REDRNNWQNVTV--VFSNCDPSNK--------IRFTFGIFADALTKNVVSS--P-FLEKYLYCLWFGLQQLSSYGQNLDTSTSVLETMFAILVAIFGLVLFALLIGNMQTYLQSITV-----------------------RLEEWRLKRRD : 423
BoCNGC10 : DARQQQWANVTN--VFKLCDAR-K--------GEFKYGMFENAITKKVVSS--N-FNERYFYCLWWGLQQLSSYGQNLSTTTFIGETTFAVLIAIFGLVLFAHLIGNMQTYLQSLTV-----------------------RLEEWRLKKRD : 422
BoCNGC13 : QPERQYWQNVTQ--VLSHCDATSS-------TTNFKFGMFAEAFTTQVATT--D-FVSN-------------SYGQNITTSVYLGETLFCITICIFGLILFTLLIGNMQTSLQSMSV-----------------------RVEEWRVKRRD : 373
BoCNGC14 : DPGRQAWMRITR--VLSNCDARNDD------DQHFQFGMFGDAFTNDVTSS--P-FFDKYFYCLWWGLRNLSSYGQSLAASTLSSETLFSCFICVAGLVFFSHLIGNVQNYLQSTTA-----------------------RLDEWRVRRRD : 395
BoCNGC9  : DPQRNSWFEWSN--ITTICKPGTR---------FYEFGIYGDAVTSTVTSS--N-FINKYFYCLWWGLKNLSSLGQNLSTSTYVGEIIFAVVMATLGLVLFALLIGNMQTYLQSTTM-----------------------RLEEWRIRRTD : 415
BoCNGC18 : TE---VLHAWKINVSANACFQEDG----------FDYGIYLKAVNLTSNCTR---WYRRYSYSLFWGFQQISTLAGNQVPSYFFGEVLFTMCIIGLGLFLFALLIGNMQNFLQSLGR-----------------------RDTEMTVRRRD : 483
BoCNGC19 : TV---TQAVLNWNVTAAACFQENG----------FRYGIYWKAVNLTHDTN----LPEKYIYSLFWGFQQISTLGGNQVPSYFIWEVLFTMAIIGLGLFLFALLIGNMQNFLQSLGR-----------------------RNMEMTLRRRD : 367
BoCNGC20 : SA---LSAAWKDNAGASACFQEDG----------FPYGIYLKAVNLTNHNS----LFTRYSYSLFWGFQQISTLAGNLIPSYFLGEVFFTMGIIGLGLLLFALLIGNMQNFLQSLGR-----------------------RNLEMTLRRRD : 520
BoCNGC21 : SE---AFAAWKGNASASACFQDGG----------FPYGIYMKAVNLTSHTS----LFTRYSYSLFWGFQQISTLAGNQVPSYFLGEVFFTMGIIGLGLLLFALLIGNMQNFLQALGR-----------------------RNLEMTLRRRD : 526
BoCNGC22 : -----HVAAWKDNASAIACFQEDG----------FPYGIYLKAVNLTNHSS----LFTRYIYSLLWGFQQISTLAGNQVPSYFLGEASFTMVIIALGLLLFPLLIGNMQNFLQALGG-----------------------RSLEMS----- : 509
BoCNGC23 : NISASLRAIWRNSASANACFQEDG----------FSYGIYLKMVNLTTHTS----LLTRYSYSLLWGFQQISTLAGNQVPSYFFPEVVFTMGIIGLGLLLFALLIGNMQNFLQSLGKRMIIFGGWKHKLHWLPSKTGSDRRSLEMTLRRRD : 550
BoCNGC25 : YARESQIALWRESESVNACFQEGG----------FSFGIYLKAVNLTTQPN----IFTRFSYSLFWGFQQISTLAGNQTPTYSVGEVYFTMGIIGLGLLLFARLIGNMHNFLQALDR-----------------------RRMEMMLRRRD : 526
BoCNGC26 : NLR----ALWRDSESVNACFQESG----------FSFGIYLNAANLTTEPN----IFTRFSFSLFWGFQQISTLAGNQTPSYSLGEVYFTMGIIGLGLLLFARLIGNMHNFLQALDR-----------------------RRTEMMLRQRD : 512
BoCNGC24 : YASASLRALWRDSDSVNACFQESG----------FSYGIYAKAVNLTSHSS----IFTRFSYSLFWGFQQISTLAGNLSPSYSVGEVFFTMGITGLGLLLFARLIGNMHNFLQALDR-----------------------RRMEMMLRRRD : 510
                                     5 5G 5  a                   g    s        3    e F   6   GL 1f  LIGN6q L                              4 e     r

                *        620         *        640         *        660         *        680         *        700         *        720         *        740         *
BoCNGC15 : IEWWMKKRHLFFGLRQRVRNYERQRWAAMRGVDECEMVQNLLEGLRRDIKYHLCLDLVRQVPLFQHMDD-LVLENICDRVKSLIFTKGETIQKEGDAVQRMLFVVRGHLQSSQL--LRDGVKSCCMLGPGNFSGDELLSWCLRRPFVERLP : 582
BoCNGC16 : IEWWMKKRQLFLGYRQRVRNYERQRWAAMRGVDECEMVQNLFEGLRRDIKYHLCLDLVRQVPLFQHMDD-LVLENICDRVKSLIFTKGETIQKEGDAVQRMLFVVRGHLQSSQL--LRDGVRSCCMLGPGNFSGDELLSWCLRRPFVERLP : 585
BoCNGC17 : MEWWMKRRQLFSRLRQRVRRFERQRWTALGGEDELELIHDLPPGLRRDIKRYLCVDLINKVPLFRGMDD-LIIDNICDRAKPRVYSKDEKIIREGDPVQRMIFIMRGRVKRNQS--LSKGVVATSTLEPGGYLGDELLSWCLRRPFIDRLP : 609
BoCNGC2  : AEQWMSHRMLFDDLRKRVRKYEQYKWQETRGVEEEVLLSSLFKDLRKDIKRHLCLNMLKTVPWFKAMDD-RLLDALCARLKPALYTENSYIVREGEPLEDMVFIMRGKLTSTTTYGGKSGFFNSVSLGVGQFCG-DLLTWALDPN-TSHFP : 559
BoCNGC3  : TEKWMSNRMLFEYLKERIRRYENYKWRKTRGIEEEALLHSLFKDLRLETKRHLYLTLINSVPWLNMMDDSWLLEALCDRVKSVFYSANSYIVKEGDPVAEMLIITKGSLKSMIGFSDITGYYDSSYLQAGDICG-DLLFWVLDPHSSSSLP : 514
BoCNGC1  : AEQWMAHRMLFEDLRKRIRRYEQYKWQETRGVEEENLLRNLFNDLRRDIKRHFCLDLLKKVPLFEIMDE-QLLDAVCDKLRPVLYTENSYAIREGDPVEEMLFVMRGKLMSATTNGGRTGFFNAVYLNASDFCGEDLLTWALDPQSSSHFP : 568
BoCNGC4  : SEQWMHHRMLFPELRERVRRYDQYKWLETRGVDEENLVSNLFKDLRRDIKRHLCLALVRRVPLFENMDE-RLLDAICERLKPCLYTEKSFLVREGDPVNEMLFIIRGRLESVTTDGGRSGFYNRSLLKEGDFCGDELLTWALDPKSGSNLP : 590
BoCNGC5  : SEQWMHHRMLFPELRERVRRYDQYKWLETRGVDEENIVSNLFKDLRRDIKRHLCLALVRRVPLFENMDE-RLLDAICMRLKPCLYTEKSFLVREGDPVNEMLFIIRGRLESVTTDGGRSGFYNRSLLKEGDFCGDELLTWALDPKSGSNLP : 600
BoCNGC6  : SEQWMHHRMLFPDLRERVRRYDQYKWLETKGVDEENLVQNLFKDLRRDIKRHLCLALVRRVPLFENMEE-RLLDAICERLKPCLYTEKSYLVREGDPVNEMLFIIRGRLESVTTDGGRSGFFNRSLLKEGDFCGEELLTWALDPKSGSNLP : 605
BoCNGC7  : SEQWMHHRMLFQDLRQRVRRYDQYKWLETRGVDEEYLVQNLFKDLRRDIKRHLCLALVRRVPLFESMDE-KLLDAICMRLKPCLFTESTYLVREGDPVDEMLFIIRGRLESVTTDGGRSGFFNYSLLKEGEFCGEELLTWALDPKSGVNLP : 588
BoCNGC8  : SEQWMHHRSLFQNLRERVRRYDQYKWLETRGVDEENIVQSLFKDLRRDIKRHLCLNLVRRVPLFANMDE-RLLDAICERLKPSLYTESTYIVREGDPVNEMLFIIRGRLESVTTDGGRSGFFNRGLLKEGDFCGEELLTWALDPKAGSNLP : 588
BoCNGC11 : TEEWMGHRQLFQDLRERVRRFVQYKWLATRGVDEETILHSLFADLRRDIQRHLCLDLVRRVPLFAQMDD-QLLDAICERLVSSLSTQGNYIVREGDPVTEMLFIIRGKLDSSTTNGGRTGFFNSITLKPGEFCGEELLAWALLPKSKVNLP : 569
BoCNGC12 : TEEWMGHRQLFQNLRERVRRFVQYKWLATRGVDEETILHSLFADLRRDIQRHLCLDLVRRVPLFAQMDD-QLLDAICERLVSSLSTQGNYIVREGDPVTEMLFIIRGKLESSTTNGGRTGFFNSITLKPGEFCGEELLVWALLPKSKVNLP : 573
BoCNGC10 : TEEWMRHRQLFEDLRDRVRRYEQYKWLAARGVDEEVLLQSLFTDLRRDIQRHLCLDLVRRVPFFSQMDD-QLLDAICERLVSSLCTEGTYLVREGDLITEMLFIIRGRLESSTTNGGRTGFFNSIILRPGDFCGEELLSWALLPKSTLNLP : 572
BoCNGC13 : TEEWMRHRQLFPELQERVRRFVQYKWLATRGVDEESILQSLFTDLRREIQRHLCLALVRRVPFFSQMDD-QLLDAICGCLVSSLSTAGTYIFREGDPVDEMLFVIRGQIESSTTNGGRSGFFNSTTLRPGDFCGEELLTWALMPNSTLNFP : 523
BoCNGC14 : TEEWMRHRQLFQELQERVRRFVQYKWLTTRGVDEEAILRALFLDLRRQIQRHLCLALVRRVPFFAQMDD-QLIDAICERLVPSLNTKDTYVTREGDPVNEMLFIIRGQMESSTTDGGRSGFFNSITLRPGDFCGEELLTWALMPNINQNLP : 545
BoCNGC9  : TEQWMHHRQLFPELRQAVRKYDQYKWLATRGVDEEALLISLFLDLRRDIKRHLCFDLVRRVPLFDQMDE-RMLDAISERLKPALCTEGTFLVREGDPVNEMLFIIRGHLDSYTTNGGRTGFFNSCLLIGPGDFCGEELLTWALDPRPVVILP : 565
BoCNGC18 : VEQWMSHRRFFKDIRKRVREVERLNWNATRGVNEELLFENMFDDLQRDIRRHLFA-FLKKVRIFSEIDE-SILDAMRARLKQRTYLKNNKVLHRGGVVKKMVFILRGKMESIGEDGYRIL------LSEGDVCGEELLTWCQERSSVNPDG : 626
BoCNGC19 : VNKWMSHRQFFEVIRERVRDAERLNWSATRGVHEELLFENMFDDLQRDIRRHLFA-FLKKVRIFSKMDD-SILDAIRERLKHKTYVNGSMVLHRGDLVKKMVIIVRGTMESIGEDGYRTV------LSEEFVCGEELLTWCQERSSVNPDG : 510
BoCNGC20 : VEQWMRHRRLFGDIRKRVREAERFNWAATRGVNEELLFKNMFDDLQREIRRHLFI-FLKKVRIFSVMDE-SILDAIRERLKQRTYIRSSMVLHRGGLVEKMVFIVRGEIESVGEDGYVLP------LSEGDVCGEELLTWCLERSSVNPDG : 663
BoCNGC21 : VEQWMSHRRLFEGIRKRVREAERFNWAATRGVNEELLFENMFDDLQRDIRRHLFI-FLKKVRIFSLMDE-SILDAIRERLKQRTYISSSTVLHRGGLVEKMVFIVRGEMESIGEDGSVLP------LSEGDVCGEELLTWCLERSSVNPDG : 669
BoCNGC22 : ------HKRFLEGIRGRVRLAEWVNWAATRGFNEKIVFENMFNDLQRDIRRHLFI-FLKKVRVFSKMDE-LILDAIRERLTYRRYISSSTVLHRGGVVEKMVFIVRGEMESFGEDGYVLP------LSEGDVCGEELLAWCLGRSSVNPDG : 646
BoCNGC23 : VEQWMSHRRLFEGIRKRVREAERFNWAATRGVNEELLFENMFDDLQGDIRRHLFI-FLKKVRIFSLMDE-SILDSIRERLRQRTYISGSTVLHRWGLVEKIVFIVRGEMESIGEDGSVLP------LSEGDVCGEELLTWCLERSAVNLDG : 693
BoCNGC25 : VEQWMSHRLLFEDIRKRVREAERFNWAATRGVNEELLFENMFDDLQRDIKRHLFK-FLKKVRIFSLMDE-SILDAIRERLKQRTYIRSSTVLHRKGLVEKMVFIVRGEMESIGEDDSVLP------LSEGDVCGEELLTWCLERSSVNPDG : 669
BoCNGC26 : VEQWMSHRLLFDDIRKRVREAERFNWAATRGVKEELIFENMFDDIQRDIKRHLFK-FLKKVWIFSLMDE-LILDAIRERLKQRRYIRSSTVLRRKGLVEKMVFIVRGEMESIGEDGSVLP------LSQGDVCGEELLTWCLERSSVDPDG : 655
BoCNGC24 : VEQWMSHRLLFEDIRKRVREAERFNWAATRGVNEELLFENMFEDLRRDIKRHLFK-FLKKVRIFSLMDE-SILDSIRERLKQRTYISSSTVLHRRGLVEKMVFIVRGGMESIGQDGSVLL------LSEGDVFGEELLTWCLERSSVNPDG : 653
            e wm h4 lp     r6R     W  t G  Ee 6    6p d6  di rhl    6  V  f  6d   66da6                      g  6  66f6 4G 6 s    g              6  g  cG eLL W l
```

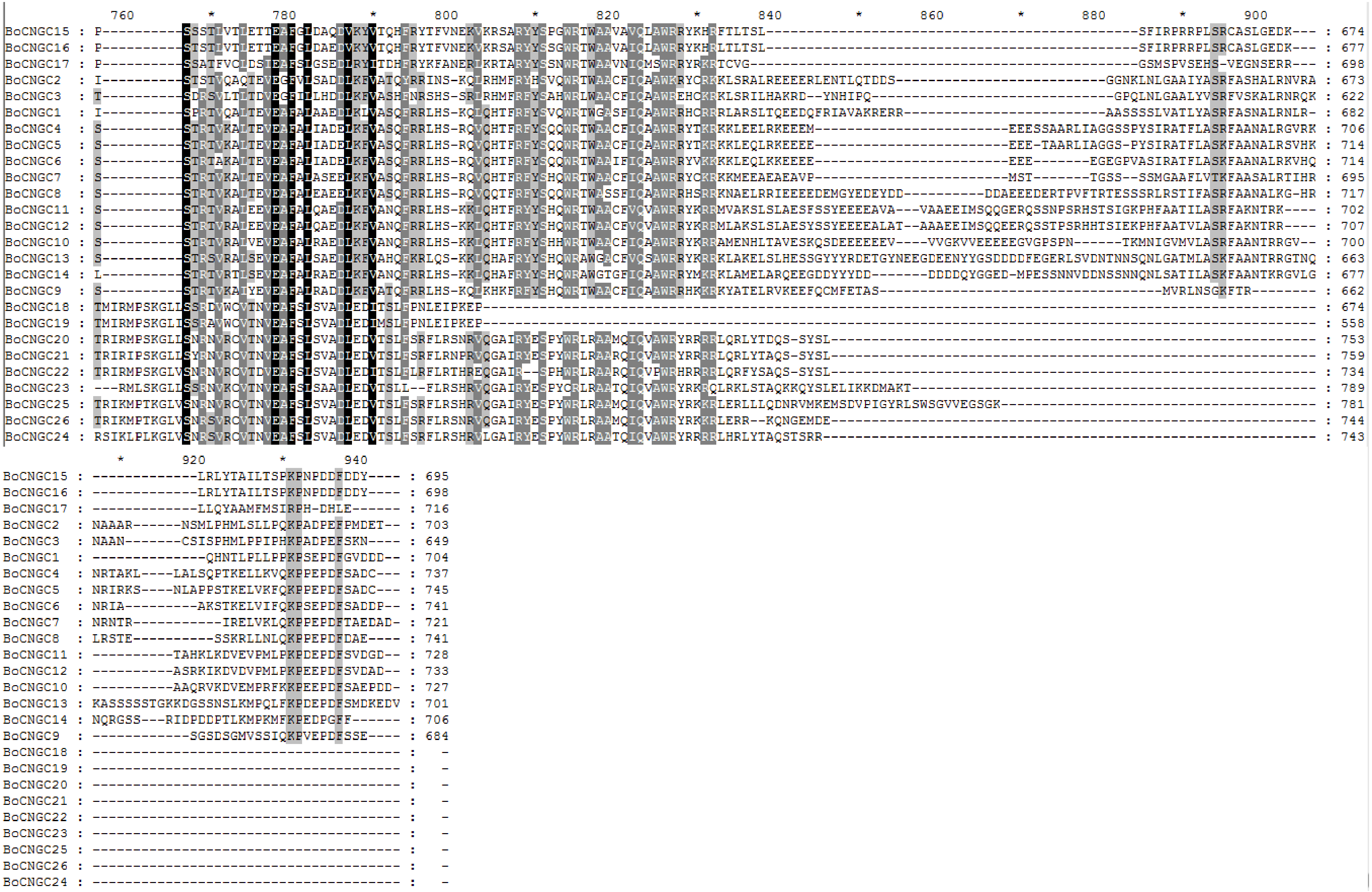

760 * 780 * 800 * 820 * 840 * 860 * 880 * 900
BoCNGC15 : P----------SSSTLVTLETTEAFGLDAQDVKYVTQHFRYTFVNEKVKRSARYYSPGWRTWAAVAVQLAWRRYKHRFTLTSL--SFIRPRRPLSRCASLGEDK--- : 674
BoCNGC16 : P----------STSTLVTLETTEAFGLDAEDVKYVTQHFRYTFVNEKVKRSARYYSGWRTWAAVAIQLAWRRYKHRLTLTSL--SFIRPRRPLSRCASLGEDK--- : 677
BoCNGC17 : P----------SSATFVCLDSIEAFSLGSEDLRYITDHFRYKFANERLKRTARYYSSNWRTWAAVNIQMSWRRYRKRTCVG---GSMSPVSEHS-VEGNSERR--- : 698
BoCNGC2 : I----------STSTVQAQTEVEGFVLSADDLKFVATQYRRINS-KQLRHMFRYHSVQWRTWAACFIQAAWKRYCRRKLSRALREEEERLENTLQTDDS---------------------------GGNKLNLGAAIYASRFASHALRNVRA : 673
BoCNGC3 : T----------SDRSVLTLTDVEGFILLHDDLKFVASHFNRSHS-SRIRHMFRYSAHWRLWAACFIQAAWREHCKRKLSRILHAKRD--YNHIPQ-----------------------------GPQLNLGAALYVSRFVSKALRNRQK : 622
BoCNGC1 : I----------SPRTVQALTEVEAFALAAEDLKLVASQFRRLHS-KQLQHTFRFYSVQWRTWGASFIQAAWRRHCRRRLARSLTQEEDQFRIAVAKRERR-------------------------AASSSSLVATLYASRFASNALRNLR- : 682
BoCNGC4 : S----------STRTVKALTEVEAFALIADELKFVASQFRRLHS-RQVQHTFRFYSQQWRTWAACFIQAAWRRYTKRKKLEELRKEEEM------------------------EEESSAARLIAGGSSPYSIRATFLASRFAANALRGVRK : 706
BoCNGC5 : S----------STRTVKALTEVEAFALIADELKFVASQFRRLHS-RQVQHTFRFYSQQWRTWAACFIQAAWRRYTKRKKLEQLRKEEEE------------------------EEE-TAARLIAGGS-PYSIRATFLASKFAANALRSVHK : 714
BoCNGC6 : S----------STRTAKALTEVEAFALIADELKFVASQFRRLHS-RQVQHTFRFYSQQWRTWAAIFIQAAWRRYVKKKKLEQLKKEEEE------------------------EEE-------EGEGPVASIRATFLASKFAANALRKVHQ : 714
BoCNGC7 : S----------STRTVKALTEVEAFALASEELKFVASQFRRLHS-RQVQHTFRFYSHQWRTWAACFIQAAWRRYCKRKKMEEAEAEAVP------------------------MST-------TGSS--SSMGAAFLVTKFAASALRTIHR : 695
BoCNGC8 : S----------STRTVKALTEVEAFALEAEELKFVASQFRRLHS-RQVQQTFRFYSQQWRTWASSFIQAAWRRHSRRKNAELRRIEEEEDEMGYEDEYDD----------DDAEEEDERTPVFTRTESSSRLRSTIFASRFAANALKG-HR : 717
BoCNGC11 : S----------STRTVRALEEVEAFALQAEDLKFVANQFRRLHS-KKLQHTFRYYSHQWRTWAACFVQVAWRRYKRRMVAKSLSLAESFSSYEEEEAVA---VAAEEIMSQQGERQSSNPSRHSTSIGKPHFAATILASRFAKNTRK---- : 702
BoCNGC12 : S----------STRTVRALEEVEAFALQAEDLKFVANQFRRLHS-KKLQHTFRYYSHQWRTWAACFVQVAWRRYKRRMLAKSLSLAESYSSYEEEEALAT--AAAEEIMSQQEERQSSTPSRHHTSIEKPHFAATVLASRFAKNTRR---- : 707
BoCNGC10 : S----------STRTVRALVEVEAFALRAEDLKFVANQFRRLHS-KKLQHTFRFYSHHWRTWAACFIQAAWRRYKRRAMENHLTAVESKQSDEEEEEEV----VVGKVVEEEEEGVGPSPN------TKMNIGVMVLASRFAANTRRGV-- : 700
BoCNGC13 : S----------STRSVRALSEVEAFALSAEDLKFVAHQFKRLQS-KKLQHAFRYYSHQWRAWGACFVQSAWRRYKRRKLAKELSLHESSGYYYRDETGYNEEGDEENYYGSDDDDFEGERLSVDNTNNSQNLGATMLASKFAANTRRGTNQ : 663
BoCNGC14 : L----------STRTVRTLSEVEAFALRAEDLKFVANQFRRLHS-KKLQHAFRYYSHQWRAWGTGFIQAAWRRYMKRKLAMELARQEEGDDYYYDD-------DDDDQYGGED-MPESSNNVDDNSSNNQNLSATILASKFAANTKRGVLG : 677
BoCNGC9 : S----------STRTVKALYEVEAFALRADDLKFVATQFRRLHS-KQLKHKFRFYSHQWRTWAACFIQAAWRRHKKRKYATELRVKEEFQCMFETAS-------------------------------MVRLNSGKFTR-------- : 662
BoCNGC18 : TMIRMPSKGLLSSRDVWCVTNVEAFSLSVADLEDITSLFPNLEIPKEP--- : 674
BoCNGC19 : TMIRMPSKGLISSRAVWCVTNVEAFSLSVADLEDIMSLFPNLEIPKEP--- : 558
BoCNGC20 : TRIRMPSKGLLSNRNVRCVTNVEAFSLSVADLEDVTSLFSRFLRSNRVQGAIRYESPYWRLRAAMQIQVAWRYRRRRLQRLYTDQS-SYSL-- : 753
BoCNGC21 : TRIRIPSKGLLSYRNVRCVTNVEAFSLSVADLEDVTSLFSRFLRNPRVQGAIRYESPYWRLRAARQIQVAWRYRRRRLQRLYTAQS-SYSL-- : 759
BoCNGC22 : TRIRMPSKGLVSNRNVRCVTDVEAFSLSVADLEDITSLFLRFLRTHREQGAIR--SPHWRLRAARQIQVEWRHRRRRLQRFYSAQS-SYSL-- : 734
BoCNGC23 : ---RMLSKGLLSSRNVKCVTNVEAFSLSAADLEDVTSLL--FLRSHRVQGAIRYESPYCRLRAATQIQVAWRYRKRQLRKLSTAQKKQYSLELIKKDMAKT--- : 789
BoCNGC25 : TRIKMPTKGLVSNRNVRCVTNVEAFSLSVADLEDVTSLFSRFLRSHRVQGAIRYESPYWRLRAAMQIQVAWRYRKRRLERLLLQDNRVMKEMSDVPIGYRLSWSGVVEGSGK------------------------------ : 781
BoCNGC26 : TRIKMPTKGLVSNRSVRCVTNVEAFSLSVADLEDVTSLFSRFLRSNRVQGAIRYESPYWRLRAAMQIQVAWRYRKRRLERR--KQNGEMDE-- : 744
BoCNGC24 : RSIKLPLKGLVSNRSVRCVTNVEAFSLSVADLEDVTSLFSRFLRSHRVLGAIRYESPYWRLRAATQIQVAWRYRRRRLHRLYTAQSTSRR--- : 743
* 920 * 940
BoCNGC15 : -------------LRLYTAILTSPKPNPDDFDDY---- : 695
BoCNGC16 : -------------LRLYTAILTSPKPNPDDFDDY---- : 698
BoCNGC17 : -------------LLQYAAMFMSIRPH-DHLE------ : 716
BoCNGC2 : NAAAR------NSMLPHMLSLLPQKPADPEFPMDET-- : 703
BoCNGC3 : NAAN-------CSISPHMLPPIPHKPADPEFSKN---- : 649
BoCNGC1 : --------------QHNTLPLLPPKPSEPDFGVDDD-- : 704
BoCNGC4 : NRTAKL----LALSQPTKELLKVQKPPEPDFSADC--- : 737
BoCNGC5 : NRIRKS----NLAPPSTKELVKFQKPPEPDFSADC--- : 745
BoCNGC6 : NRIA---------AKSTKELVIFQKPSEPDFSADDP-- : 741
BoCNGC7 : NRNTR-----------IRELVKLQKPPEPDFTAEDAD- : 721
BoCNGC8 : LRSTE----------SSKRLLNLQKPPEPDFDAE---- : 741
BoCNGC11 : ----------TAHKLKDVEVPMLPKPDEPDFSVDGD-- : 728
BoCNGC12 : ----------ASRKIKDVDVPMLPKPEEPDFSVDAD-- : 733
BoCNGC10 : ----------AAQRVKDVEMPRFKKPEEPDFSAEPDD- : 727
BoCNGC13 : KASSSSSTGKKDGSSNSLKMPQLFKPDEPDFSMDKEDV : 701
BoCNGC14 : NQRGSS---RIDPDDPTLKMPKMFKPEDPGFF------ : 706
BoCNGC9 : ------------SGSDSGMVSSIQKPVEPDFSSE---- : 684
BoCNGC18 : -------------------------------------- : -
BoCNGC19 : -------------------------------------- : -
BoCNGC20 : -------------------------------------- : -
BoCNGC21 : -------------------------------------- : -
BoCNGC22 : -------------------------------------- : -
BoCNGC23 : -------------------------------------- : -
BoCNGC25 : -------------------------------------- : -
BoCNGC26 : -------------------------------------- : -
BoCNGC24 : -------------------------------------- : -
