# Supplementary material for: Comprehensive genomic analysis of the CNGC gene family in Brassica oleracea: novel insights into synteny, structures, and transcript profiles
Source: BMC Genomics. 2017 Nov 13;18:869. doi: 10.1186/s12864-017-4244-y (PMC5683364; doi:10.1186/s12864-017-4244-y)
Supplement: Supplementary file 9 — Multiple sequence alignment of CNGC-encoded proteins of Arabidopsis and B. oleracea. Multiple sequence alignment was performed by clustal X2 and viewed by GeneDoc software package. (PDF 1208 kb) [file 12864_2017_4244_MOESM9_ESM.pdf]

Additional file 9. Multiple sequence alignment of *CNGC*-encoded proteins of *Arabidopsis* and *B. oleracea*. Multiple sequence alignment was performed by clustal X2 and viewed by GeneDoc software package.

```
                    *        20         *        40         *        60         *        80         *       100         *       120         *       140         *
BoCNGC15 : --------MATEQEFTR------ASRVSGASS----------SVGCYSDEDYKDEEEEEEEEEEEMEE----------------------------------------------------------T-------EKDEEEEEPRVRVTCG :  61
AtCNGC4  : --------MATEQEFTR------ASRFSRDSS----------SVGYYSEEDNTEEEDEEEEEMEEIEE----------------------------------------------------------E-------EEEEEEEDPRIGLTCG :  61
BoCNGC16 : --------MATEQEFTR------ASRVSRASS----------SIGYYSDEDYTTEEEEDEEE-EMEEQ----------------------------------------------------------E-------EEEEEEEETHVGVTCG :  60
BoCNGC17 : --------MPSHTNFLFRWIGLFSQKLRRETTGISEN-----NVGESSSS--DDTPVLSSGECYACTQ----------------------------------------------------------VGVPAFHSTSCDQANAPEWRASAG :  77
AtCNGC2  : --------MPSHPNFIFRWIGLFSDKFRRQTTGIDENSNLQINGGDSSSSGSDETPVLSSVECYACTQ----------------------------------------------------------VGVPAFHSTSCDQAHAPEWRASAG :  84
BoCNGC1  : -------------------MGFGRDNRVRWFKE-PSS-TEYGYGR---------RARPSLNAVLN--------------------------------------------------------------------NVRRGFE-KGSDKIRTF :  51
AtCNGC13 : -------------------MAFGRNNRVRFRDW-ISEGTEYGYGRN--------KARPSLNTVLK--------------------------------------------------------------------NVRRG-----------L :  43
AtCNGC10 : ------------------------MILFRFKDEGKPLSSEYGYGR---------KARPSLDRVFK--------------------------------------------------------------------NVKWG-----------F :  38
BoCNGC2  : ----------------MEMMNLKRNTFVKFTEN------EDSWN------------RPSVTSVIKK-------------------------------------------------------------------TVRRSFE-KGSEKIRNF :  48
AtCNGC3  : ------------------MMNPQRNKFVRFNGN------DDEFSTK--------TTRPSVSSVMK--------------------------------------------------------------------TVRRSFE-KGSEKIRTF :  49
AtCNGC11 : -------------------MNLQRRKFVRLDSTG-------------------------VDGKLK--------------------------------------------------------------------SVRGR-----------L :  27
AtCNGC12 : -------------------MNHRRSKFARIDSMG-------------------------VDGKLK--------------------------------------------------------------------SVRGR-----------L :  27
BoCNGC3  : --------------------------MERASTMQS------------------------VHENIK--------------------------------------------------------------------SVRGQ-----------L :  21
AtCNGC1  : -------------------MNFRQEKFVRFQDWKSDKTSSDVEYSGKNE--IQTGIFQRTISSISD-------------------------------------------------------------------KFYRSFE-SSSARIKLF :  61
BoCNGC5  : -----MFD-CGTNGVKSQVISGHREKFIRLESMDSRYSQSSDNTGLNKCTLNIQ-APKRFAQGSK----------------------------------------------------------------TSSGSFKNGFR-KGSEGLWSI :  78
AtCNGC6  : -----MFDTCGPKGVKSQVISGQRENFVRLDSMDSRYSQSSE-TGLNKCTLNIQGGPKRFAQGSK----------------------------------------------------------------ASSGSFKKGFR-KGSEGLWSI :  79
BoCNGC4  : ------------MESKSQVISGHREKFIRLDSMDPR----SPEAGLNRCTLNIQ-RPKRFTQATK----------------------------------------------------------------ASSGSFKKGFR-KGSEGLWSI :  68
BoCNGC6  : -----MFD-CVKKSVKSQVISGQREKFVRLDSMDSRYSQGSE-AGLSKCTLNLQ-GQSRANGGTGQGN----------------------------------------------------------NNNASSGSFKKGFR-RGSKGLWSL :  83
AtCNGC9  : -----MLD-CGKKAVKSQVISGRLEKFVRLDSMDSRYSQTSD-TGLNRCTLNLQ-GPTRGGGAQG--------------------------------------------------------------NNVSSGSFKKGFR-KGSKGLWSI :  79
BoCNGC7  : -------------------MAGKPQTFVSVDDLDFKLPSSSSLTRQHNYSSSIS-GPLHPIQGSH----------------------------------------------------------------NASGSFKKRFQ-KGSKGLKSI :  65
AtCNGC5  : -------------------MAGKRENFVRVDDLDSRLPSSS-VAFQQNYASNFS-GQLHPIHASN----------------------------------------------------------------ETSRSFKKGIQ-KGSKGLKSI :  64
BoCNGC8  : -------------MYKSQYISGQREKFVRLDDLDS--SASHATGMMTKRNCFG-FPVKNRGSEKKR----------------------------------------------------------------ASKSFREGVK-IGSEGLFSI :  69
AtCNGC7  : --------------------------------------------MMMQRNCFG-FNLKNRGGEKKK----------------------------------------------------------------ASKSFREGVKKIRSEGLITI :  41
AtCNGC8  : -------------MYKSQYISGHREKFVRLDDTDSRVSMSSNATGMKKRSCFGLFNVTSRGGGKTKN---------------------------------------------------------------TSKSFREGVK-IGSEGLKTI :  73
BoCNGC9  : -------------------MGYGNSRSVRFEEDSEVTKPQAVHEET---------AEKLKFKING---------------------------------------------------------------------AQISPRKNVKKMTR-- :  51
AtCNGC15 : -------------------MGYGNSRSVRFQEDQEVVH----GGES---------GVKLKFKING---------------------------------------------------------------------TQIN---NVKMMSK-- :  44
BoCNGC10 : -------------------MELSKDKLLMLYSDKKEPKEAIWAVN----------DPMSKSYKLS---------------------------------------------------------------------LPSALKPPPDNLLA-- :  50
AtCNGC17 : -------------------MELRKDKLLMFYSEGKESKEAKWAVN----------DPMSKSYKLS---------------------------------------------------------------------LPSALRP--DNLLP-- :  48
BoCNGC11 : -------------------MEFKRDNTVRFYGDEKQTLEVTEKRLP---------LPMFKSSAAL---------------------------------------------------------------------FQKQELGTSKKS---- :  49
BoCNGC12 : -------------------MEFKRDNTVRFYGEEKQTLEATEKRQP---------LPMLKPSTTQ---------------------------------------------------------------------FLKPELVIPKKTNKTR :  53
AtCNGC14 : -------------------MEFKRDNTVRFYGDEKQTIEVGEKR-----------VPLFKSTTAP---------------------------------------------------------------------FMKQEVLPKKSK---T :  48
BoCNGC13 : ---------------------------------------------------------MNKIRSLR---------------------------------------------------------------------FLLPETITSA------ :  18
AtCNGC18 : ---------------------------------------------------------MNKIRSLR---------------------------------------------------------------------CLLPETITSAST---- :  20
BoCNGC14 : ---------------------------------------------M---------SNLHLHTSAR---------------------------------------------------------------------FRNFPTAFSRR----- :  22
AtCNGC16 : ---------------------------------------------M---------SNLHLYTSAR---------------------------------------------------------------------FRNFPTTFSLR----- :  22
BoCNGC18 : MASPMENDDVPMLPASDTSSSS-RTMPFTSRSRSTSLANNSSTIDVF-NSSTVVLGYTNHLGTQRRPP-LVQMSDPLSSTRSPEPRFALPPPS----------------------------------------------TGASSDSVGAS : 101
BoCNGC19 : MASSNGYDDVPMLPVSCTSSSS-RTRPFTFRSRSVSLSNTSSTIDVFENSSTVVLSYTDPLGTQRQPQ-LVQMGDPISSTRN------LELVS----------------------------------------------KHAHP--YGTT :  94
BoCNGC20 : ----MEKDDVPMLPVSDSSSLS-RTRPFTSRSRSVSLANTSSIIDGF-DSSTVVLGYTGPLRTRRRPP-LVQMSGPLSS-TRSSEPLFLPPPP----TSTRDVSSSQPERYPSFTALEHKNS-EEEFVLKHAHLLRSGQLGMCNDPYCTT : 137
BoCNGC21 : MASPKENDDVPMLPISDTS----RTRPFTSRSRSVSLSNTCSTIDGF-DSSTVVLGYTGPLRAQRRPP-LVQMSGPLSS-TRNPEPLFLLPPP----DSVG-ISSSQPERYPSFATLEHKKS-DDEFVLKHANLLRSGQLGMCNDPYCTT : 137
AtCNGC20 : MASHNENDDIPMLPISDPSSRT-RARAFTSRSRSVSLSNPTSSIEGF-DTSTVVLGYTGPLRTQRRPP-LVQMSGPLTS-TRKHEPLFLPHPSS---DSVG--VSSQPERYPSFAALEHKNSSEDEFVLKHANLLRSGQLGMCNDPYCTT : 141
BoCNGC22 : MAPPNEKDDVPMLPISSSSSSS-RTRSFTSRFRSTSLANTSSAIDGF-DSSNVVLGYTGPLQTYGRPA-FVQMSASLPS-TLIPEPLFLHPTPTGGSSHSIG-VSSSQPESCP-FAALEHKNS-DDELGLG-----SGQLEVCNDPYCTT : 138
BoCNGC23 : MVSPNKNDKIHILPISDASSSSSQTRVFTSRTRSVPLSNPTDETG---NSNAVTLGYAGSLLSQ-RPP-LVPMTGPLSSSTRRPEPLFPRPAP-----------PPTRRSSGYFGDLEEVNSSDNDELLKHAHRLRSGKLGMCNDPYCTT : 134
BoCNGC24 : MISPNENDQV----SIPEATSRAHTGAFNFKNRSVSLSNSTYYIDGC-DKSKVALGYTVPIRTQRRPP------GPLYSTLRP--ESLLPPS-----------IEPPDS----SSTVDVRSEDE--SVVKNANILTSGQLGMCNEPYCTT : 120
AtCNGC19 : ---------------------MAHTRTFTSRNRSVSLSNPSFSIDGF-DNSTVTLGYTGPLRTQRIRPPLVQMSGPIHSTRRT--EPLFSPSP----------QESPDS----SSTVDVPPEDD--FVFKNANLLRSGQLGMCNDPYCTT : 110
BoCNGC25 : MASPNEKDEFPILLPVSEARPRANTRALNSRNRSVSFSNSTYSTNRV-DNSSVVLGYTGPLRTQRRPPPSVQMSSPLYSTRRP-DQSFFPPSP----------VQPPDSSLSSS-TVDVPSEED-EVVLKNANLLKSGQLGMCNDPYCTT : 136
BoCNGC26 : MAYPNESDEFPMLRQVPEARSRAQSRALHSRNRSVSFSDSTYSTNRV-ENS------SGPRRTQSRPSPSVHMSGPLYDTRRPPDQSFFPPSP----------VQPPESSLSSSTTVDIPSEEVVEALLKNANLLKSGQLGMCNDPYCTT : 133
```

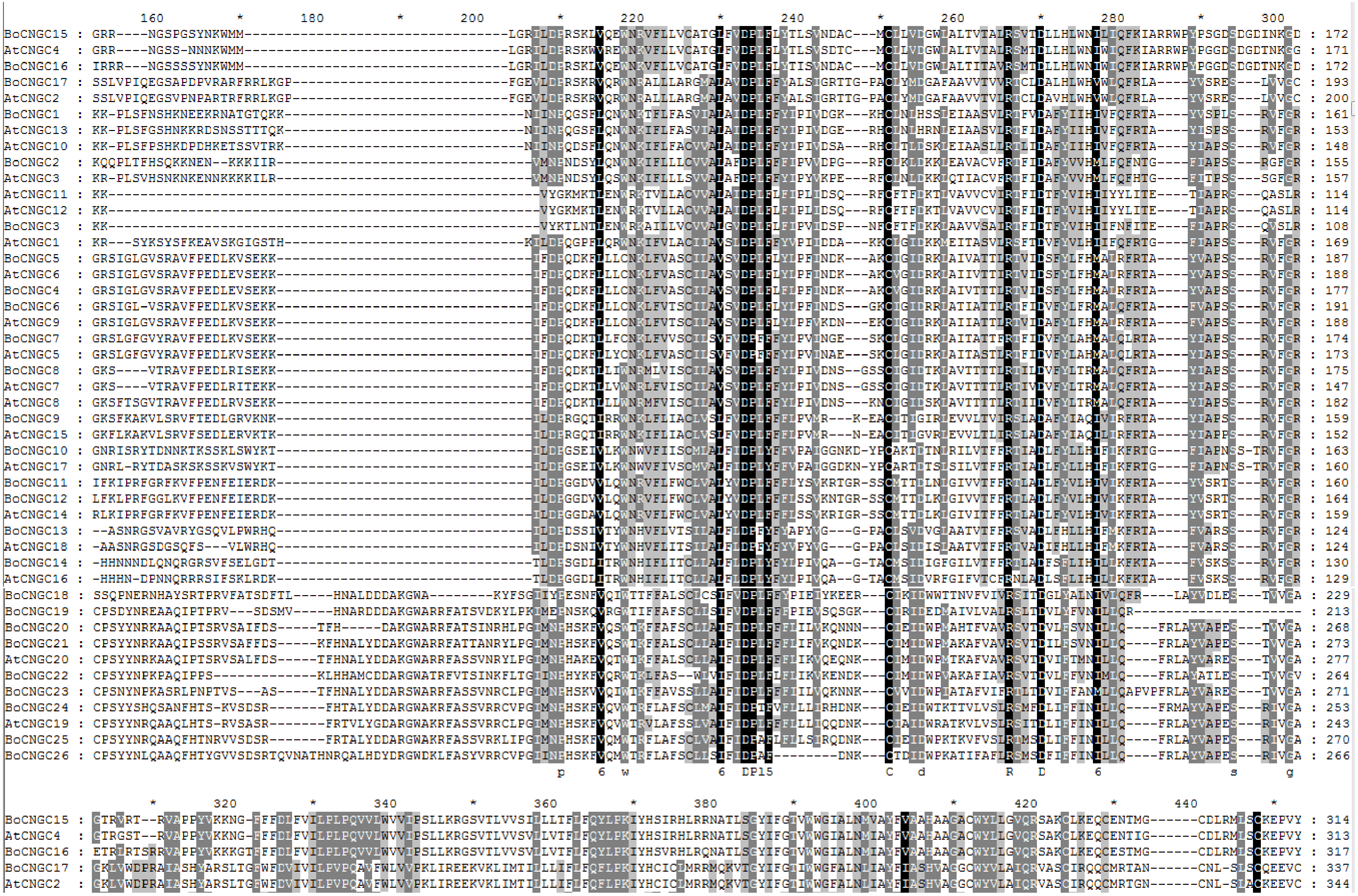

160 * 180 * 200 * 220 * 240 * 260 * 280 * 300
BoCNGC15 : 172
AtCNGC4 : 171
BoCNGC16 : 172
BoCNGC17 : 193
AtCNGC2 : 200
BoCNGC1 : 161
AtCNGC13 : 153
AtCNGC10 : 148
BoCNGC2 : 155
AtCNGC3 : 157
AtCNGC11 : 114
AtCNGC12 : 114
BoCNGC3 : 108
AtCNGC1 : 169
BoCNGC5 : 187
AtCNGC6 : 188
BoCNGC4 : 177
BoCNGC6 : 191
AtCNGC9 : 188
BoCNGC7 : 174
AtCNGC5 : 173
BoCNGC8 : 175
AtCNGC7 : 147
AtCNGC8 : 182
BoCNGC9 : 159
AtCNGC15 : 152
BoCNGC10 : 163
AtCNGC17 : 160
BoCNGC11 : 160
BoCNGC12 : 164
AtCNGC14 : 159
BoCNGC13 : 124
AtCNGC18 : 124
BoCNGC14 : 130
AtCNGC16 : 129
BoCNGC18 : 229
BoCNGC19 : 213
BoCNGC20 : 268
BoCNGC21 : 273
AtCNGC20 : 277
BoCNGC22 : 264
BoCNGC23 : 271
BoCNGC24 : 253
AtCNGC19 : 243
BoCNGC25 : 270
BoCNGC26 : 266
p 6 w 6 DP15 C d R D 6 s g
* 320 * 340 * 360 * 380 * 400 * 420 * 440 *
BoCNGC15 : 314
AtCNGC4 : 313
BoCNGC16 : 317
BoCNGC17 : 337
AtCNGC2 : 344

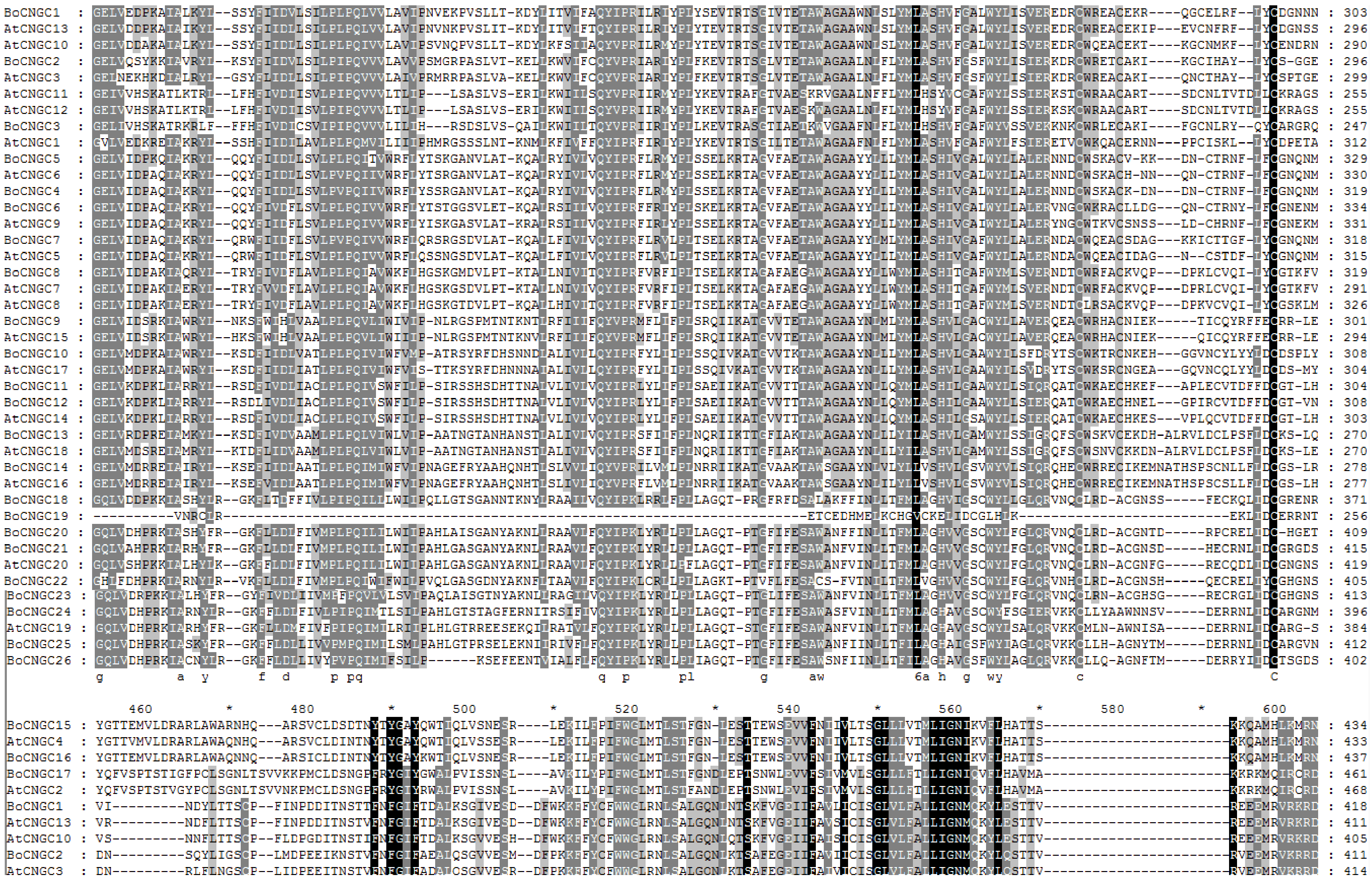
BoCNGC1 : GELVEDPKAIALKYI--SSYFIIDVLSILPLPQLVVLAVIPNVEKPVSLLT-KDYLITVIFAQYIPRILRIYPLYSEVTRTSGIVTETAWAGAAWNLSLYMLASHVFGALWYLISVEREDRCWREACEKR----QGCELRF--LYCDGNNN : 303
AtCNGC13 : GELVDDPKAIAIKYI--SSYFIIDLLSILPLPQLVVLAVIPNVNKPVSLIT-KDYLITVIFTQYIPRILRIYPLYTEVTRTSGIVTETAWAGAAWNLSLYMLASHVFGALWYLISVEREDRCWREACEKIP---EVCNFRF--LYCDGNSS : 296
AtCNGC10 : GELVDDAKAIALKYI--SSYFIIDLLSILPLPQIVVLAVIPSVNQPVSLLT-KDYLKFSIIAQYVPRILRMYPLYTEVTRTSGIVTETAWAGAAWNLSLYMLASHVFGALWYLISVEREDRCWQEACEKT----KGCNMKF--LYCENDRN : 290
BoCNGC2 : GELVQSYKKIAVRYL--KSYFIIDVLSILPIPQVVVLAVVPSMGRPASLVT-KELLKWVIFCQYVPRIARIYPLFKEVTRTSGLVTETAWAGAALNLFLYMLASHVFGSFWYLISIERKDRCWRETCAKI----KGCIHAY--LYCS-GGE : 296
AtCNGC3 : GELNEKHKDIALRYL--GSYFLIDLLSILPIPQVVVLAIVPRMRRPASLVA-KELLKWVIFCQYVPRIARIYPLFKEVTRTSGLVTETAWAGAALNLFLYMLASHVFGSFWYLISIERKDRCWREACAKI----QNCTHAY--LYCSPTGE : 299
AtCNGC11 : GEIVVHSKATLKTRL--LFHFIVDIISVLPIPQVVVLTLIP---LSASLVS-ERILKWIIISQYVPRIIRMYPLYKEVTRAFGTVAESKRVGAALNFFLYMIHSYVCGAFWYLSSIERKSTCWRAACART----SDCNLTVTDLLCKRAGS : 255
AtCNGC12 : GEIVVHSKATLKTRL--LFHFIVDIISVLPIPQVVVLTLIP---LSASLVS-ERILKWIIISQYVPRIIRMYPLYKEVTRAFGTVAESKWAGAALNLFLYMIHSYVFGAFWYLSSIERKSKCWRAACART----SDCNLTVTDLLCKRAGS : 255
BoCNGC3 : GELIVHSKATRKRLF--FFHFIVDICSVIPIPQVVVLILLH---RSDSLVS-QAILKWIITQYVPRIIRIYPLLKEVTRASGTIAETKWVGAAFNLFLYMIHSHVFGAFWYVSSVEKKNKCWRLECAKI----FGCNLRY--QYCARGRQ : 247
AtCNGC1 : GVLVEDKREIAKRYL--SSHFIIDILAVLPLPQMVILIIIPHMRGSSSLNT-KNMLKFIVFFQYIPRFIRIYPLYKEVTRTSGILTETAWAGAAFNLFLYMLASHVFGAFWYLFSIERETVCWKQACERNN---PPCISKL--LYCDPETA : 312
BoCNGC5 : GELVIDPKQIAKRYL--QQYFIIDLLSVLPLPQITVWRFLYTSKGANVLAT-KQALRYIVIVQYIPRFLRMYPLSSELKRTAGVFAETAWAGAAYYLLLYMLASHIVGALWYLLALERNNDCWSKACV-KK---DN-CTRNF-LFCGNQNM : 329
AtCNGC6 : GELVIDPAQIAKRYL--QQYFIIDLLSVLPVPQIIVWRFLYTSRGANVLAT-KQALRYIVLVQYIPRFLRMYPLSSELKRTAGVFAETAWAGAAYYLLLYMLASHIVGALWYLLALERNNDCWSKACH-NN---QN-CTRNF-LFCGNQNM : 330
BoCNGC4 : GELVIDPAQIAKRYL--QQYFIIDLLSVLPVPQIIVWRFLYSSRGANVLAT-KQALRYIVLVQYIPRFLRMYPLSSELKRTAGVFAETAWAGAAYYLLLYMLASHIVGALWYLLALERNNDCWSKACK-DN---DN-CTRNF-LFCGNQNM : 319
BoCNGC6 : GELVIDPAQIAKRYL--QQYFIVDFLSVLPLPQIVVWRFLYTSTGGSVLET-KQALRSIILVQYIPRFFRIYPLSKELKRTAGVFAETAWAGAAYYLLLYMLASHIVGALWYLLALERVNGCWKRACLLDG---QN-CTRNY-LFCGNENM : 334
AtCNGC9 : GELVIDPAQIAKRYL--QQYFIIDFLSVLPLPQIVVWRFLYISKGASVLAT-KRALRSIILVQYIPRFIRLYPLSSELKRTAGVFAETAWAGAAYYLLLYMLASHIVGAIWYLLALERYNGCWTKVCSNSS---LD-CHRNF-LFCGNEKM : 331
BoCNGC7 : GELVIDPAQIAKRYL--QRWFIIDFLSVLPVPQIVVWRFLQRSRGSDVLAT-KQALLFIVLVQYIPRFLRVLPLTSELKRTAGVFAETAWAGAAYYMLLYMLASHIVGAFWYLLALERNDACWQEACSDAG---KKICTTGF-LYCGNQNM : 318
AtCNGC5 : GELVIDPAQIAKRYL--QRWFIIDFLSVLPLPQIVVWRFLQSSNGSDVLAT-KQALLFIVLVQYIPRFLRVLPLTSELKRTAGVFAETAWAGAAYYLLLYMLASHIVGAFWYLLALERNDACWQEACIDAG---N--CSTDF-LYCGNQNM : 315
BoCNGC8 : GELVIDPAKIAQRYL--TRYFIVDFLAVLPLPQIAVWKFLHGSKGMDVLPT-KTALLNIVITQYIPRFVRFIPLTSELKKTAGAFAEGAWAGAAYYLLWYMLASHITGAFWYMLSVERNDTCWRFACKVQP---DPKLCVQI-LYCGTKFV : 319
AtCNGC7 : GELVIDPAKIAERYL--TRYFVVDFLAVLPLPQIAVWKFLHGSKGSDVLPT-KTALLNIVIVQYIPRFVRFIPLTSELKKTAGAFAEGAWAGAAYYLLWYMLASHITGAFWYMLSVERNDTCWRFACKVQP---DPRLCVQI-LYCGTKFV : 291
AtCNGC8 : GELVIDPAKIAERYL--TRYFIVDFLAVLPLPQIAVWKFLHGSKGTDVLPT-KQALLHIVITQYIPRFVRFIPLTSELKKTAGAFAEGAWAGAAYYLLWYMLASHITGAFWYMLSVERNDTCLRSACKVQP---DPKVCVQI-LYCGSKLM : 326
BoCNGC9 : GELVIDSRKIAWRYL--NKSFWIHLVAALPLPQVLIWIVIP-NLRGSPMTNTKNTLRFIIIFQYVPRMFLIFPLSRQIIKATGVVTETAWAGAAYNLMLYMLASHVLGACWYLLAVERQEACWRHACNIEK-----TICQYRFFECRR-LE : 301
AtCNGC15 : GELVIDSRKIAWRYL--HKSFWIHLVAALPLPQVLIWIIIP-NLRGSPMTNTKNVLRFIIIFQYVPRMFLIFPLSRQIIKATGVVTETAWAGAAYNLMLYMLASHVLGACWYLLAVERQEACWRHACNIEK-----QICQYRFFECRR-LE : 294
BoCNGC10 : GELVMDPKAIAWRYL--KSDFIIDLVATLPLPQIVIWFVMP-ATRSYRFDHSNNDLALIVLLQYIPRFYLIIPLSSQIVKATGVVTKTAWAGAAYNLLLYMLASHVLGAAWYILSFDRYTSCWKTRCNKEH---GGVNCYLYYLDCDSPLY : 308
AtCNGC17 : GELVMDPKAIAWRYL--KSDFIIDLIATLPLPQIVIWFVIS-TTKSYRFDHNNNALALIVLLQYIPRFYLIIPLSSQIVKATGVVTKTAWAGAAYNLLLYMLASHVLGAAWYILSVDRYTSCWKSRCNGEA---GQVNCQLYYLDCDS-MY : 304
BoCNGC11 : GELVKDPKLIARRYL--RSDFIVDLIACLPLPQIVSWFILP-SIRSSHSDHTTNALVLIVLVQYIPRLYLIFPLSAEIIKATGVVTTTAWAGAAYNLLQYMLASHILGAAWYLLSIQRQATCWKAECHKEF---APLECVTDFFDCGT-LH : 304
BoCNGC12 : GELVKDPKLIARRYL--RSDLIVDLIACLPLPQIVSWFILP-SIRSSHSDHTTNALVLIVLVQYIPRLYLIFPLSAEIIKATGVVTTTAWAGAAYNLLQYMLASHILGAAWYLLSIERQATCWKAECHNEL---GPIRCVTDFFDCGT-VN : 308
AtCNGC14 : GELVKDPKLIARRYL--RSDFIVDLIACLPLPQIVSWFILP-SIRSSHSDHTTNALVLIVLVQYIPRLYLIFPLSAEIIKATGVVTTTAWAGAAYNLLQYMLASHILGSAWYLLSIERQATCWKAECHKES---VPLQCVTDFFDCGT-LH : 303
BoCNGC13 : GELVRDPREIAMKYL--KSDFIVDVAAMLPLPQLVIWLVIP-AATNGTANHANSTLALIVLVQYIPRSFLIFPLNQRIIKTTGFIAKTAWAGAAYNLLLYILASHVLGAMWYLLSSIGRQFSCWSKVCEKDH-ALRVLDCLPSFLDCKS-LQ : 270
AtCNGC18 : GELVMDSREIAMRYL--KTDFLIDVAAMLPLPQLVIWLVIP-AATNGTANHANSTLALIVLVQYIPRSFLIIFPLNQRIIKTTGFIAKTAWAGAAYNLLLYILASHVLGAMWYLLSSIGRQFSCWSNVCKKDN-ALRVLDCLPSFLDCKS-LE : 270
BoCNGC14 : GELVMDRREIAIRYL--KSEFIIDLAATLPLPQIMIWFVIPNAGEFRYAAHQNHTLSLVVLIQYVPRILVMLPLNRRIIKATGVAAKTAWSGAAYNLVLYLIVSHVLGSVWYVLSIQRQHECWRRECIKEMNATHSPSCNLLFLDCGS-LR : 278
AtCNGC16 : GELVMDRREIAIRYL--KSEFVIDLAATLPLPQIMIWFVIPNAGEFRYAAHQNHTLSLIVLIQYVPRFLVMLPLNRRIIKATGVAAKTAWSGAAYNLILYLIVSHVLGSVWYVLSIQRQHECWRRECIKEMNATHSPSCSLLFLDCGS-LH : 277
BoCNGC18 : GQLVDDPKKIASHYLR--GKFLTLFFIVLPIPQILLLWIIPQLLGTSGANNTKNYLRAAIIVQYIPKLRRLFPLLAGQT-PRGFRFDSALAKFFINLLTFMLAGHVIGSCWYLLGLQRVNQCLRD-ACGNSS-----FECKQLIDCGRENR : 371
BoCNGC19 : ----------VNRCLR--ETCEDHMELKCHGVCKELIDCGLHLK----------------------------EKLIDCERRNT : 256
BoCNGC20 : GQLVDHPRKIASHYFR--GKFLLDLFIVMPLPQILILWIIPAHLAISGANYAKNLLRAAVLFQYIPKLYRLLPLLAGQT-PTGFIFESAWANFFINLLTFMLAGHVVGSCWYLFGLQRVNQCLRD-ACGNTD-----RPCRELLDC-HGET : 409
BoCNGC21 : GQLVAHPRKIARHYFR--GKFLLDLFIVMPLPQILILWIIPAHLGASGANYAKNLLRAAVLFQYIPKLYRLLPLLAGQT-PTGFIFESAWANFVINLLTFMLAGHVVGSCWYLFGLQRVNQCLRD-ACGNSD-----HECRNLLDCGRGDS : 415
AtCNGC20 : GQLVSHPRKIALHYLK--GKFFLDLFIVMPLPQILILWIIPAHLGASGANYAKNLLRAAVLFQYIPKLYRLLPFLAGQT-PTGFIFESAWANFVINLLTFMLAGHVVGSCWYLFGLQRVNQCLRN-ACGNFG-----RECQDLIDCGNGNS : 419
BoCNGC22 : GHLFDHPRKIARNYLR--VKFLLDLFIVMPLPQIWIFWILPVQLGASGDNYAKNFLTAAVLFQYIPKLCRLLPLLAGKT-PTVFLFESACS-FVTNILTFMIVGHVVGSCWYLFGLQRVNHCLRD-ACGNSH-----QECRELLYCGHGNS : 405
BoCNGC23 : GQLVDRPKKIALHYFR--GYFIVDLIIVMPFPQVLVLSVIPAQLAISGTNYAKNLLRAGILVQYIPKLYRLLPLLAGQT-PTGLIFESAWANFVINLLTFMLAGHVVGSCWYLFGLQRVNQCLRN-ACGHSG-----RECRGLLDCGHGNS : 413
BoCNGC24 : GQLVDHPRKIARNYLR--GKFFLDLFIVLPIPQIMTLSILPAHLGTSTAGFERNITRSIFIVQYIPKLYRLLPLLAGQT-PTGFIFESAWASFVINLLTFMLAGHAVGSCWYFSGIERVKKCLLYAAWNNSV-----DERRNLLDCARGNM : 396
AtCNGC19 : GQLVDHPRKIARHYFR--GKFLLDMFIVFPIPQIMILRIIPLHLGTRREESEKQILRATVLFQYIPKLYRLLPLLAGQT-STGFIFESAWANFVINLLTFMLAGHAVGSCWYLSALQRVKKCMLN-AWNISA-----DERRNLLDCARG-S : 384
BoCNGC25 : GQLVDHPRKIASKYFR--GKFFLDLLIVVPMPQIMILSMLPAHLGTPRSELEKNIIRIVFLFQYIPKLYRLLPLLAGQT-PTGFIFESAWANFIINLLTFILAGHAIGSFWYIAGLQRVKKCLLH-AGNYTM-----DERRNLLDCARGVN : 412
BoCNGC26 : GQLVDHPRKIACNYLR--GKFFLDLLIVYPVPQIMIFSILP------KSEFEENTVIALFLFQYIPKLYRLLPLIAGQT-PTGFIFESAWSNFIINLLTFILAGHAVGSFWYLAGLQRVKKCLLQ-AGNFTM-----DERRYILDCTSGDS : 402
g a y f d p pq q p pl g aw 6a h g wy c C
460 * 480 * 500 * 520 * 540 * 560 * 580 * 600
BoCNGC15 : YGTTEMVLDRARLAWARNHQ---ARSVCLDSDTNYTYGAYQWTIQLVSNESR----LEKILFPIFWGLMTLSTFGN-LESTTEWSEVVFNIIVLTSGLLLVTMLIGNIKVFLHATTS----------------------KKQAMHLKMRN : 434
AtCNGC4 : YGTTVMVLDRARLAWAQNHQ---ARSVCLDINTNYTYGAYQWTIQLVSSESR----LEKILFPIFWGLMTLSTFGN-LESTTEWSEVVFNIIVLTSGLLLVTMLIGNIKVFLHATTS----------------------KKQAMHLKMRN : 433
BoCNGC16 : YGTTEMVLDRARLAWAQNNQ---ARSICLDINTNYTYGAYKWTIQLVSNESR----LEKILFPIFWGLMTLSTFGN-LESTTEWSEVVFNIIVLTSGLLLVTMLIGNIKVFLHATTS----------------------KKQAMHLKMRN : 437
BoCNGC17 : YQFVSPTSTIGFPCLSGNLTSVVKKPMCLDSNGPFRYGIYGWALPVISSNSL----AVKILYPIFWGLMTLSTFGNDLEPTSNWLEVVFSIVMVLSGLLLFTLLIGNIQVFLHAVMA----------------------KKRKMQIRCRD : 461
AtCNGC2 : YQFVSPTSTVGYPCLSGNLTSVVNKPMCLDSNGPFRYGIYRWALPVISSNSL----AVKILYPIFWGLMTLSTFANDLEPTSNWLEVIFSIVMVLSGLLLFTLLIGNIQVFLHAVMA----------------------KKRKMQIRCRD : 468
BoCNGC1 : VI---------NDYLTTSCP--FINPDDITNSTTFNFGIFTDALKSGIVESD--DFWKKFFYCFWWGLRNLSALGQNLNTSKFVGEIIFAVLICISGLVLFALLIGNMQKYLESTTV----------------------REEPMRVRKRD : 418
AtCNGC13 : VR---------NDFLTTSCP--FINPDDITNSTVFNFGIFTDALKSGIVESD--DFWKKFFYCFWWGLRNLSALGQNLNTSKFVGEIIFAVSICISGLVLFALLIGNMQKYLESTTV----------------------REEPMRVRKRD : 411
AtCNGC10 : VS---------NNFLTTSCP--FLDPGDITNSTIFNFGIFTDALKSGVVESH--DFWKKFFYCFWWGLRNLSALGQNLQTSKFVGEIIFAISICISGLVLFALLIGNMQKYLESTTV----------------------REEPMRVRKRD : 405
BoCNGC2 : DN---------SQYLIGSCP--LMDPEEIKNSTVFNFGIFAEALQSGVVESM--DFPKKFFYCFWWGLRNLSALGQNLKTSAFEGEIIFAVLICISGLVLFALLIGNMQKYLQSTTV----------------------RVEPMRVKRRD : 411
AtCNGC3 : DN---------RLFLNGSCP--LIDPEEITNSTVFNFGIFADALQSGVVESR--DFPKKFFYCFWWGLRNLSALGQNLKTSAFEGEIIFAIVICISGLVLFALLIGNMQKYLQSTTV----------------------RVEPMRVKRRD : 414

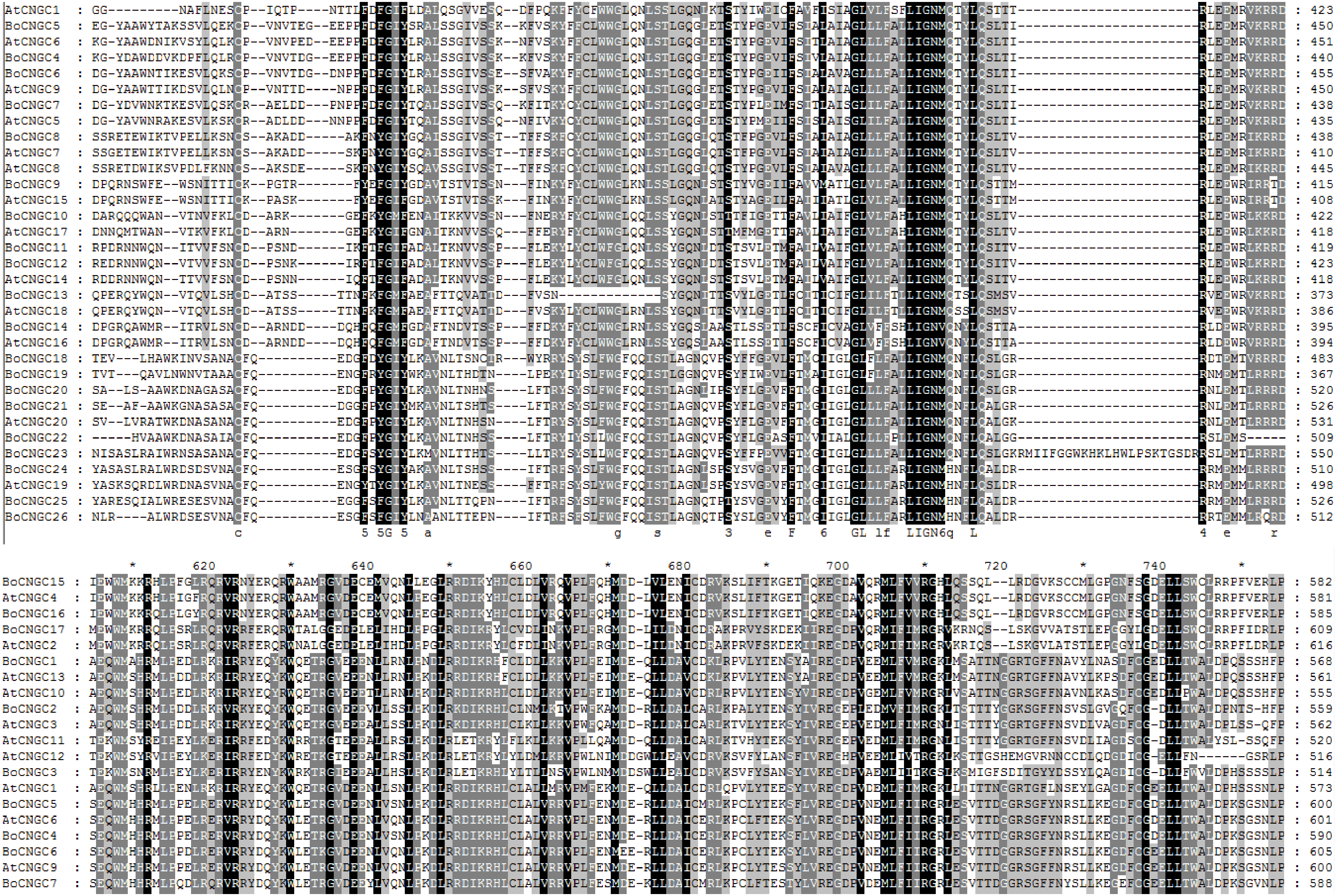

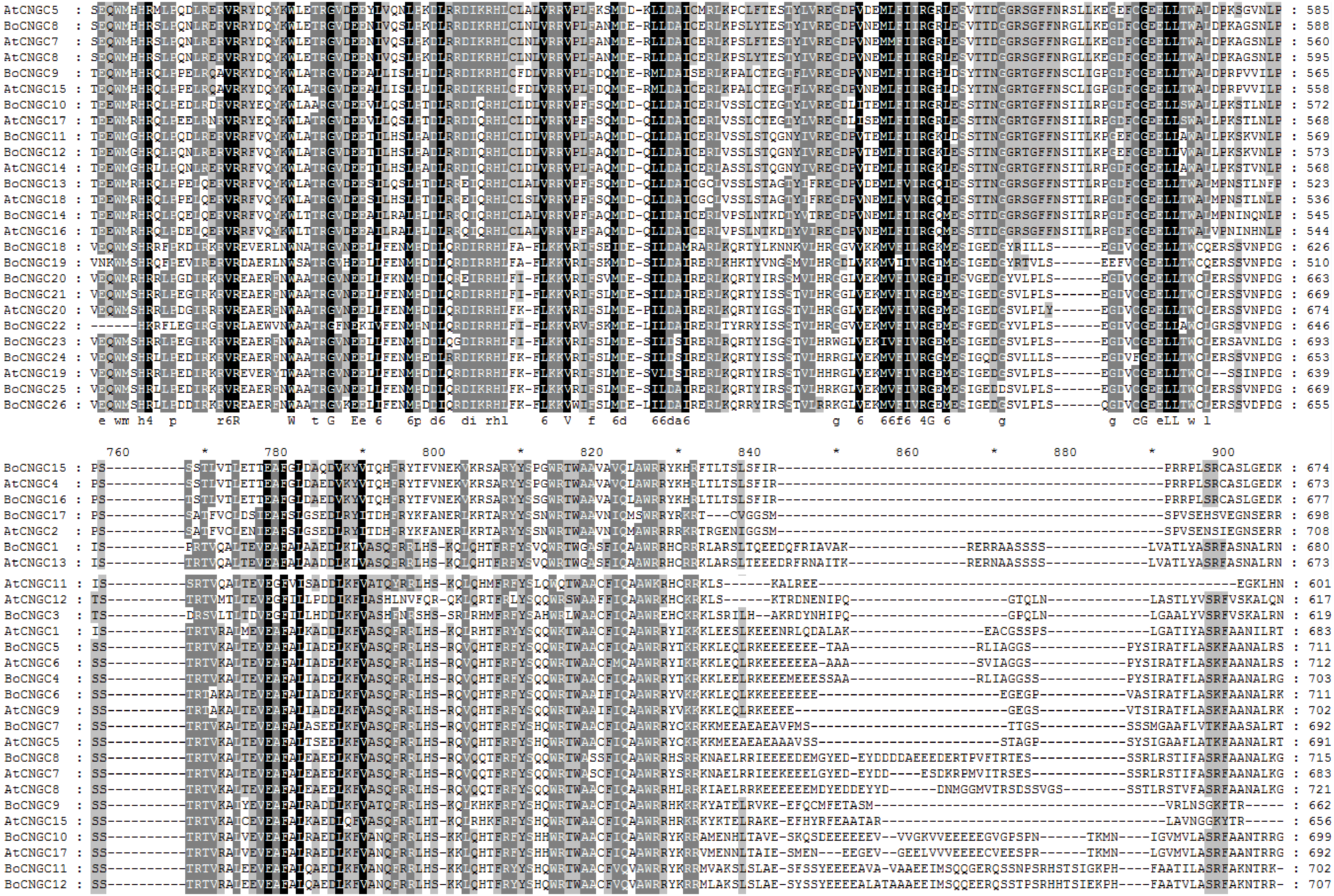

AtCNGC5 : 585
BoCNGC8 : 588
AtCNGC7 : 560
AtCNGC8 : 595
BoCNGC9 : 565
AtCNGC15 : 558
BoCNGC10 : 572
AtCNGC17 : 568
BoCNGC11 : 569
BoCNGC12 : 573
AtCNGC14 : 568
BoCNGC13 : 523
AtCNGC18 : 536
BoCNGC14 : 545
AtCNGC16 : 544
BoCNGC18 : 626
BoCNGC19 : 510
BoCNGC20 : 663
BoCNGC21 : 669
AtCNGC20 : 674
BoCNGC22 : 646
BoCNGC23 : 693
BoCNGC24 : 653
AtCNGC19 : 639
BoCNGC25 : 669
BoCNGC26 : 655
760 780 800 820 840 860 880 900
BoCNGC15 : 674
AtCNGC4 : 673
BoCNGC16 : 677
BoCNGC17 : 698
AtCNGC2 : 708
BoCNGC1 : 680
AtCNGC13 : 673
AtCNGC11 : 601
AtCNGC12 : 617
BoCNGC3 : 619
AtCNGC1 : 683
BoCNGC5 : 711
AtCNGC6 : 712
BoCNGC4 : 703
BoCNGC6 : 711
AtCNGC9 : 702
BoCNGC7 : 692
AtCNGC5 : 691
BoCNGC8 : 715
AtCNGC7 : 683
AtCNGC8 : 721
BoCNGC9 : 662
AtCNGC15 : 656
BoCNGC10 : 699
AtCNGC17 : 692
BoCNGC11 : 702
BoCNGC12 : 707

```
AtCNGC14 : SS----------TRTVRALEEVEAFALQAGDLKFVANQFRRLHS-KKLQHTFRYYSHQWRTWAACFVQVAWRRYKRRKLAKSLSLAE-SFSSYDEEEAVA-VAATEEMSHEGEAQSGAKARHHTSNVKPH----FAATILASRFAKNTRR- : 701
BoCNGC13 : SS----------TRSVRALSEVEAFALSAEDLKFVAHQFKRLQS-KKLQHAFRYYSHQWRAWGACFVQSAWRRYKRRKLAKELSLHE-SSGYYYRDETGYNEEGDE--ENYYGSDDDDFEGERLSVDNTNNSQNLGATMLASKFAANTRRG : 660
AtCNGC18 : SS----------TRSVRALSEVEAFALSAEDLKFVAHQFKRLQS-KKLQHAFRYYSHQWRAWGACFVQSAWRRYKRRKLAKELSLHE-SSGYYYPDETGYNEEDEETREYYYGSDE---EGG--SMDNTN----LGATILASKFAANTRRG : 666
BoCNGC14 : LS----------TRTVRTLSEVEAFALRAEDLKFVANQFRRLHS-KKLQHAFRYYSHQWRAWGTGFIQAAWRRYMKRKLAMELARQEEGDDYYYDDDDDDQYGGEDMPESSNN-VDDN------SSNNQN----LSATILASKFAANTKRG : 674
AtCNGC16 : LS----------TRTVRTLSEVEAFALRAEDLKFVANQFRRLHS-KKLQHAFRYYSHQWRAWGTCFIQAAWRRYMKRKLAMELARQEEEDDYFYDDDGDYQF-EEDMPESNNNNGDEN------SSNNQN----LSATILASKFAANTKRG : 673
BoCNGC18 : TMIRMPSKGLLSSRDVWCVTNVEAFSLSVADLEDITSLFPNLEIPKEP------------------------------------------------------------------------------------------------------- : 674
BoCNGC19 : TMIRMPSKGLISSRAVWCVTNVEAFSLSVADLEDIMSLFPNLEIPKEP------------------------------------------------------------------------------------------------------- : 558
BoCNGC20 : TRIRMPSKGLLSNRNVRCVTNVEAFSLSVADLEDVTSLFSRFLRSNRVQGAIRYESPYWRLRAAMQIQVAWRYRRRRLQRLYTDQS-SYSL------------------------------------------------------------ : 753
BoCNGC21 : TRIRIPSKGLLSYRNVRCVTNVEAFSLSVADLEDVTSLFSRFLRNPRVQGAIRYESPYWRLRAARQIQVAWRYRRRRLQRLYTAQS-SYSL------------------------------------------------------------ : 759
AtCNGC20 : TRIRMPSKGLLSSRNVRCVTNVEAFSLSVADLEDVTSLFSRFLRSHRVQGAIRYDSPYWRLRAARQIQVAWRYRRRRLHRLCTPQS-SYSL------------------------------------------------------------ : 764
BoCNGC22 : TRIRMPSKGLVSNRNVRCVTDVEAFSLSVADLEDITSLFLRFLRTHREQGAIR--SPHWRLRAARQIQVPWRHRRRRLQRFYSAQS-SYSL------------------------------------------------------------ : 734
BoCNGC23 : ---RMLSKGLLSSRNVKCVTNVEAFSLSAADLEDVTSLL--FLRSHRVQGAIRYESPYCRLRAATQIQVAWRYRKRQLRKLSTAQKKQYSLELIKKDMAKT-------------------------------------------------- : 789
BoCNGC24 : RSIKLPLKGLVSNRSVRCVTNVEAFSLSVADLEDVTSLFSRFLRSHRVLGAIRYESPYWRLRAATQIQVAWRYRRRRLHRLYTAQSTSRR------------------------------------------------------------- : 743
AtCNGC19 : TRIKMPPKGLVSNRNVRCVTNVEAFSLSVADLEDVTSLFSRFLRSHRVQGAIRYESPYWRLRAAMQIQVAWRYRKRQLQRLNTAHSNSNR------------------------------------------------------------- : 729
BoCNGC25 : TRIKMPTKGLVSNRNVRCVTNVEAFSLSVADLEDVTSLFSRFLRSHRVQGAIRYESPYWRLRAAMQIQVAWRYRKKRLERLLLQDNRVMKEMSDVPIGYRLSWSGVVEGSGK--------------------------------------- : 781
BoCNGC26 : TRIKMPTKGLVSNRSVRCVTNVEAFSLSVADLEDVTSLFSRFLRSNRVQGAIRYESPYWRLRAAMQIQVAWRYRKKRLERR--KQNGEMDE------------------------------------------------------------ : 744
                        r        EaF 6    6  6     r           r  s  w   aa   q aw

              *       920         *       940         *       960         *       980         *
BoCNGC15 : LRLYTAILTS----------------PKPNPDDFDDY------------------------------------------------ : 695
AtCNGC4  : LRLYAAILTS----------------PKPNPDDFDDY------------------------------------------------ : 694
BoCNGC16 : LRLYTAILTS----------------PKPNPDDFDDY------------------------------------------------ : 698
BoCNGC17 : LLQYAAMFMS----------------IRPH-DHLE-------------------------------------------------- : 716
AtCNGC2  : LLQYAAMFMS----------------IRPH-DHLE-------------------------------------------------- : 726
BoCNGC1  : LRQHNT---------------LPLLPPKPSEPDFGVDDD---------------------------------------------- : 704
AtCNGC13 : LRTNN----------------LPLLPPKPSEPDFSLRNPIYASRFASHALRNLRANAAARNSRFPHMLTLLPQKPADPEFPMDET : 742
AtCNGC10 : LRHNISNL--------PPRYTLPLLPQKPTEPDFTANHTTDP------------------------------------------- : 706
BoCNGC2  : VRANAAARN------SMLPHMLSLLPQKPADPEFPMDET---------------------------------------------- : 703
AtCNGC3  : ------------------------------------------------------------------------------------- :   -
AtCNGC11 : TLQNDDSG-------GNKLNLGAAIYA---------------------------------------------------------- : 621
AtCNGC12 : RRKDTADC-------SSSPDMSPPVPHKPADLEFAKAEA---------------------------------------------- : 649
BoCNGC3  : RQKNAANC-------SISPHMLPPIPHKPADPEFSKN------------------------------------------------ : 649
AtCNGC1  : IRRSGSVRKP-----RMPERMPPMLLQKPAEPDFNSDD----------------------------------------------- : 716
BoCNGC5  : VHKNRIRKSNL----APPSTKELVKFQKPPEPDFSADC----------------------------------------------- : 745
AtCNGC6  : VHKNRTAKSTL----LLSSTKELVKFQKPPEPDFSAEDH---------------------------------------------- : 747
AtCNGC9  : VHKNRIEAK---------STIELVKYQKPSEPDFSADDTS--------------------------------------------- : 733
BoCNGC7  : IHRNRNTRIR-----------ELVKLQKPPEPDFTAEDAD--------------------------------------------- : 721
AtCNGC5  : IHRNRNTKIR-----------DLVKLQKPPEPDFTAD------------------------------------------------ : 717
BoCNGC8  : -HRLRSTESS----------KRLLNLQKPPEPDFDAE------------------------------------------------ : 741
AtCNGC7  : -HRLRSSESS----------KTLINLQKPPEPDFDAE------------------------------------------------ : 709
AtCNGC8  : -HKLRVTESS----------KSLMNLTKPSEPDFEALDTDDLN------------------------------------------ : 753
BoCNGC9  : -SGSDSG--------------MVSSIQKPVEPDFSSE------------------------------------------------ : 684
AtCNGC15 : -SGSDSG--------------MMSSIQKPVEPDFSSE------------------------------------------------ : 678
BoCNGC10 : VAAQRVKDV------------EMPRFKKPEEPDFSAEPDD--------------------------------------------- : 727
AtCNGC17 : VAAQRVKDV------------ELPRFKKPEEPDFSAEHDD--------------------------------------------- : 720
BoCNGC11 : -TAHKLKDV------------EVPMLPKPDEPDFSVDGD---------------------------------------------- : 728
BoCNGC12 : -ASRKIKDV------------DVPMLPKPEEPDFSVDAD---------------------------------------------- : 733
AtCNGC14 : -TAHKLKDV------------EIPMLPKPDEPDFSVDD----------------------------------------------- : 726
BoCNGC13 : TNQKASSSSSTGKKDGSSNSLKMPQLFKPDEPDFSMDKEDV-------------------------------------------- : 701
AtCNGC18 : TNQKASSSS-TGKKDGSSTSLKMPQLFKPDEPDFSIDKEDV-------------------------------------------- : 706
BoCNGC14 : VLGNQRGSS---RIDPDDPTLKMPKMFKPEDPGFF-------------------------------------------------- : 706
AtCNGC16 : VLGNQRGST---RIDPDHPTLKMPKMFKPEDPGFF-------------------------------------------------- : 705
BoCNGC18 : ------------------------------------------------------------------------------------- :   -
BoCNGC19 : ------------------------------------------------------------------------------------- :   -
BoCNGC20 : ------------------------------------------------------------------------------------- :   -
BoCNGC21 : ------------------------------------------------------------------------------------- :   -
AtCNGC20 : ------------------------------------------------------------------------------------- :   -
BoCNGC22 : ------------------------------------------------------------------------------------- :   -
BoCNGC23 : ------------------------------------------------------------------------------------- :   -
BoCNGC24 : ------------------------------------------------------------------------------------- :   -
AtCNGC19 : ------------------------------------------------------------------------------------- :   -
BoCNGC25 : ------------------------------------------------------------------------------------- :   -
BoCNGC26 : ------------------------------------------------------------------------------------- :   -
```
