# Supplementary material for: Comprehensive genomic analysis of the CNGC gene family in Brassica oleracea: novel insights into synteny, structures, and transcript profiles
Source: BMC Genomics. 2017 Nov 13;18:869. doi: 10.1186/s12864-017-4244-y (PMC5683364; doi:10.1186/s12864-017-4244-y)
Supplement: Supplementary file 10 — Multiple sequence alignment of CNGC-encoded proteins of B. oleracea and B. rapa. Multiple sequence alignment was performed by clustal X2 and viewed by GeneDoc software package. (PDF 1436 kb) [file 12864_2017_4244_MOESM10_ESM.pdf]

Additional file 10. Multiple sequence alignment of CNGC-encoded proteins of *B. oleracea* and *B. rapa*. Multiple sequence alignment was performed by clustal X2 and viewed by GeneDoc software package.

```
                     *        20         *        40         *        60         *        80         *       100         *       120         *       140         *
Bra004537 : --------------------------------------------MEMMNLKRNTFVKFTENEDSWNRPS--------VTSVIKKTVRRSFEKGSEKIRTFKQQPLTFHSQKKNENKKK-------------------------------- :  66
BoCNGC2   : --------------------------------------------MEMMNLKRNTFVKFTENEDSWNRPS--------VTSVIKKTVRRSFEKGSEKIRNFKQQPLTFHSQKKNENKKK-------------------------------- :  66
Bra034281 : ----------------------------------------------------------------MERAS--------TIQSVHENIK--SVRGQLKKVYKTLKTLENWRK---------------------------------------- :  36
BoCNGC3   : ----------------------------------------------------------------MERAS--------TMQSVHENIK--SVRGQLKKVYKTLNTLENWRK---------------------------------------- :  36
Bra003323 : ------------------------------------------------------MTIFSVQSTLFTRAS--------VALLSSNGLKRFSFASSFSSAALYSPPLPKTKKR--------------------------------------- :  49
Bra031515 : -----------------------------------------------------------------------------------------------------MKTLN-TRK---------------------------------------- :   8
Bra000937 : -------------------------------------------MGFGRDSRVR-FKEPSSTEFGYGRRAR------PSLNAVLDNVRRGFEKGSDKIRTFKRPLSFNSHKNEEKRNATGTQ----------------------------- :  71
BoCNGC1   : -------------------------------------------MGFGRDNRVRWFKEPSSTEYGYGRRAR------PSLNAVLNNVRRGFEKGSDKIRTFKKPLSFNSHKNEEKRNATGTQ----------------------------- :  72
Bra022632 : --------------------------------------------MNFRQEKFVRFQDWKSDKTSDVEYSGRNEPPNGIFRRTITSISDKFHRSSARIKTFRRTYKSYSFKEAVSKGIDS------------------------------- :  75
Bra003081 : ------MKFRLTSLTLSSCGSRVLHSSHKEGYTTISCASTNSPTMNFRPDKSVRFHDWKSDKASDVEYS---EVPDGLYRRAISSISDKFHRSSLRIKMLR---TSYSFKETVSKGIVS------------------------------- : 107
Bra020402 : MTFASLPYHFCRASDRGLSRSSVIELAIMAGKPQTFVSVDDLDFKLPSSSSLTRQHNYSSSISGPLHPIQ-------GSHNTSGSFKKRFQKGSKGLKSIGRSLGFGVYRAVFPEDLKVS------------------------------ : 113
BoCNGC7   : ----------------------------MAGKPQTFVSVDDLDFKLPSSSSLTRQHNYSSSISGPLHPIQ-------GSHNASGSFKKRFQKGSKGLKSIGRSLGFGVYRAVFPEDLKVS------------------------------ :  85
Bra024067 : -----------------------------------------MDSRYSQ-GAEAGLNKCTLNLQGPSRANG--AGQGNNNNASSGSFKKGFRRGSKGLWSLGRSIGLGVSRAVFPEDLKVS------------------------------ :  76
BoCNGC6   : ---------------MFDCVKKSVKSQVISGQREKFVRLDSMDSRYSQ-GSEAGLSKCTLNLQGQSRANGG-TGQGNNNNASSGSFKKGFRRGSKGLWSLGRSIGL-VSRAVFPEDLKVS------------------------------ : 102
Bra039221 : ---------------------MESKSQVISGHREKFIRLDSMDPR----SPEAGLNRCTINIQRPKRFTQ-------ANKTSSGSFKKGFRKGSEGLWSIGRSIGLGVSRAVFPEDLEVS------------------------------ :  88
BoCNGC4   : ---------------------MESKSQVISGHREKFIRLDSMDPR----SPEAGLNRCTLNIQRPKRFTQ-------ATKASSGSFKKGFRKGSEGLWSIGRSIGLGVSRAVFPEDLEVS------------------------------ :  88
Bra032132 : ---------------MFDCGTNGVKSQVISGHREKFIRLESMDSRYSQSSDNTGLNKCTLNIQAPKRFAQ-------GSKTSSGSFKKGFRKGSEGLWSIGRSIGLGVSRAVFPEDLKVS------------------------------ :  98
BoCNGC5   : ---------------MFDCGTNGVKSQVISGHREKFIRLESMDSRYSQSSDNTGLNKCTLNIQAPKRFAQ-------GSKTSSGSFKNGFRKGSEGLWSIGRSIGLGVSRAVFPEDLKVS------------------------------ :  98
Bra026086 : ---------------------------------------------------MMTKRNCFG-FPVKNRGSE--------KKRASKSFREGVKIGSEGLFSIGK----SVTRAVFPEDLRIS------------------------------ :  56
BoCNGC8   : ----------------------MYKSQYISGQREKFVRLDDLDSSASHATGMMTKRNCFG-FPVKNRGSE--------KKRASKSFREGVKIGSEGLFSIGK----SVTRAVFPEDLRIS------------------------------ :  85
Bra008733 : -------------------------------------------------MNKIRSLRFL---------------------------LPETITSAASN---RGSVAVRYGSQVLPWR---------------------------------- :  37
BoCNGC13  : -------------------------------------------------MNKIRSLRFL---------------------------LPETITSAASN---RGSVAVRYGSQVLPWR---------------------------------- :  37
Bra018089 : ----------------------------------------------MSNLHLHTSARFRN--------------------------FPTAFSRRHHN---NNDLQNQRGRSVFSELG--------------------------------- :  42
BoCNGC14  : ----------------------------------------------MSNLHLHTSARFRN--------------------------FPTAFSRRHHN---NNDLQNQRGRSVFSELG--------------------------------- :  42
Bra011186 : -----------------------------------------------MELRKDKILMLYSDKKEPKEAIWAVN-DPMSKS--YKLSLPSALKPPDNNLLSGNRISRYTDNNKSKSSKPSW------------------------------ :  70
BoCNGC10  : -----------------------------------------------MELSKDKLLMLYSDKKEPKEAIWAVN-DPMSKS--YKLSLPSALKPPPDNLLAGNRISRYTDNNKTKSSKLSW------------------------------ :  70
Bra007839 : -----------------------------------------------MEFKRDNTVRFYGEEKQTLEATEKRQPLPMFKPSTTQFLKPELVIPKKTNKTRLFKLPRFGGLKVFPENFEIE------------------------------ :  73
BoCNGC12  : -----------------------------------------------MEFKRDNTVRFYGEEKQTLEATEKRQPLPMLKPSTTQFLKPELVIPKKTNKTRLFKLPRFGGLKVFPENFEIE------------------------------ :  73
Bra032081 : -----------------------------------------------MEFKRDNTVRFYGDEKQTLEVTEKRLPLPMFKSSAAPFQKQELGTSKKS----IFKIPRFGRFKVFPENFEIE------------------------------ :  69
BoCNGC11  : -----------------------------------------------MEFKRDNTVRFYGDEKQTLEVTEKRLPLPMFKSSAALFQKQELGTSKKS----IFKIPRFGRFKVFPENFEIE------------------------------ :  69
Bra011963 : -----------------------------------------------MGYGNSRSVRFEEDSEVTKPQAVHEETAVKLKFKINGAQISPRKNVKKMTR--GKSFKDKVLSRVFTEDLGRV------------------------------ :  71
BoCNGC9   : -----------------------------------------------MGYGNSRSVRFEEDSEVTKPQAVHEETAEKLKFKINGAQISPRKNVKKMTR--GKSFKAKVLSRVFTEDLGRV------------------------------ :  71
Bra021266 : --------------------------MASPKE--NDDVPML-PISDTS---RTRPFTSRSRSVSLSNTCSTIDGF-DSSTVVLGYTGPLRAQRRPPL-VQMSGPLSSTRTPEPLFLLPPPS-----DSVGISSSQPERYPSFATLEHKKS : 111
BoCNGC21  : --------------------------MASPKE--NDDVPML-PISDTS---RTRPFTSRSRSVSLSNTCSTIDGF-DSSTVVLGYTGPLRAQRRPPL-VQMSGPLSSTRNPEPLFLLPPP------DSVGISSSQPERYPSFATLEHKKS : 110
BoCNGC20  : ------------------------------ME--KDDVPML-PVSDSSSLSRTRPFTSRSRSVSLANTSSIIDGF-DSSTVVLGYTGPLRTRRRPPL-VQMSGPLSSTRSSEPLFLPPPPT-----STRDVSSSQPERYPSFTALEHKNS : 110
BoCNGC22  : --------------------------MAPPNE--KDDVPML-PISSSSSSSRTRSFTSRFRSTSLANTSSAIDGF-DSSNVVLGYTGPLQTYGRPAF-VQMSASLPSTLIPEPLFLHPTPTGG-SSHSIGVSSSQPESCP-FAALEHKNS : 117
Bra031529 : --------------------------MAPPNE--NDDALKL-PVLDTSSSSRTRPFTFRSRNVSLPNTSSTSEGL-DSSTVVLRYTDPHRTQRPPPS-VQMNGPLFSTSSPEPLILLPPPSTGGSSDPVGVSSSQPERYPSFAALEHDNS : 119
Bra001676 : --------------------------MASPME--NDDVPML-PASDTSSSSRTMPFTSRSRSTSLANNSSTIDVF-NSSTVVLGYTDPLGTQRRPPL-VQMSYPLSSTRSPEPRFALPPP-----------STGASYDSVGASSSQPNER : 108
BoCNGC18  : --------------------------MASPME--NDDVPML-PASDTSSSSRTMPFTSRSRSTSLANNSSTIDVF-NSSTVVLGYTNHLGTQRRPPL-VQMSDPLSSTRSPEPRFALPPP-----------STGASSDSVGASSSQPNER : 108
Bra001678 : --------------------------MASSNG--YDDVPML-PVSCTSSSSRTRPFTSRSRSVSLSNTSSTIDVFENSSTVVLGYTDPLGTQRQPQL-VQMGDPISSTRNLELV------------------------------------ :  84
BoCNGC19  : --------------------------MASSNG--YDDVPML-PVSCTSSSSRTRPFTFRSRSVSLSNTSSTIDVFENSSTVVLSYTDPLGTQRQPQL-VQMGDPISSTRNLELVSKHAHPY----------GTTCPSDYNREAAQIPTPR : 110
Bra021265 : --------------------------MTSPNE--NDQV----SIPEATSRAHTRAFNFKNRSVSLSNSTYYIDGC-DNSKVALGYTVPIRTQRRPP------GPLYSTPRP--ESHFPPS----------IEPPDS---SS-TVDVRSED :  95
BoCNGC24  : --------------------------MISPNE--NDQV----SIPEATSRAHTGAFNFKNRSVSLSNSTYYIDGC-DKSKVALGYTVPIRTQRRPP------GPLYSTLRP--ESLLPPS----------IEPPDS---SS-TVDVRSED :  95
Bra022232 : --------------------------MASPNE--KDEFPILLPVPEARSRANTRAFNSRNRSVSFSNSTYSTNRV-DNSSVVLGYTGPLRTQRRLPPSVQMSGPLYSTRRP-DQSFFPPSP---------VQPPDS---SS-TVDVPSEE : 107
BoCNGC25  : --------------------------MASPNE--KDEFPILLPVSEARPRANTRALNSRNRSVSFSNSTYSTNRV-DNSSVVLGYTGPLRTQRRPPPSVQMSSPLYSTRRP-DQSFFPPSP---------VQPPDSSLSSS-TVDVPSEE : 110
Bra022233 : --------------------------MASPNENESDEFPMLRQFPEARS----RALHSRNRSISFSDSTYSSNRV-ENS------SGPRRTQSRPSPSVHMSGPLYDTRRP-DQSFFPPSP---------VQPPASSLSSS-TVDIPSEE : 102
BoCNGC26  : --------------------------MAYPNE--SDEFPMLRQVPEARSRAQSRALHSRNRSVSFSDSTYSTNRV-ENS------SGPRRTQSRPSPSVHMSGPLYDTRRPPDQSFFPPSP---------VQPPESSLSSSTTVDIPSEE : 106
Bra029958 : ---------------------------------------MLPISDASSSSSQTRVFTSRTRSVPLSNPTEETG---NSKAATLGYAGSLPSQRPPLFP--MTGPLSSSTRR------------------------SSGYFGDLEEVNSSD :  82
BoCNGC23  : --------------------------MVSPNK--NDKIHILPISDASSSSSQTRVFTSRTRSVPLSNPTDETG---NSNAVTLGYAGSLLSQRPPLVP--MTGPLSSSTRRPEPLFPRPAP---------PPTRRSSGYFGDLEEVNSSD : 108
Bra022702 : ------------------------MATEQEFTR------ASRVSGASSSVGYYSDEDYKDEEEEEEEEE-----------EEMEETEKDEEEEEPRVRVTCGGRR----NGSPGSYNKWMM----------------------------- :  76
BoCNGC15  : ------------------------MATEQEFTR------ASRVSGASSSVGCYSDEDYKDEEEEEEEEE-----------EEMEETEKDEEEEEPRVRVTCGGRR----NGSPGSYNKWMM----------------------------- :  76
Bra003001 : ------------------------MATEQEFTR------ASRVSRASSSIGYYSDEDYTTDEEENEEEE-----------MEELE-EAEEEEEETHVGGTCGIRRR---NGSSSSYNKWMV----------------------------- :  76
BoCNGC16  : ------------------------MATEQEFTR------ASRVSRASSSIGYYSDEDYTTEEEEDEEEE-----------MEEQE-EEEEEEEETHVGVTCGIRRR---NGSSSSYNKWMM----------------------------- :  76
Bra008699 : ------------------------MPSHTNFLFRWIGLFSQKLRRETTEISENNGGESSSSSDDTPVLSSGECYACTQVGVPAFHSTSCDQANAPEWRASAGSSLVPIQEGSAPSPVRARIRR--------------------------- :  99
BoCNGC17  : ------------------------MPSHTNFLFRWIGLFSQKLRRETTGISENNVGESSSS-DDTPVLSSGECYACTQVGVPAFHSTSCDQANAPEWRASAGSSLVPIQEGSAPDPVRARFRR--------------------------- :  98
```

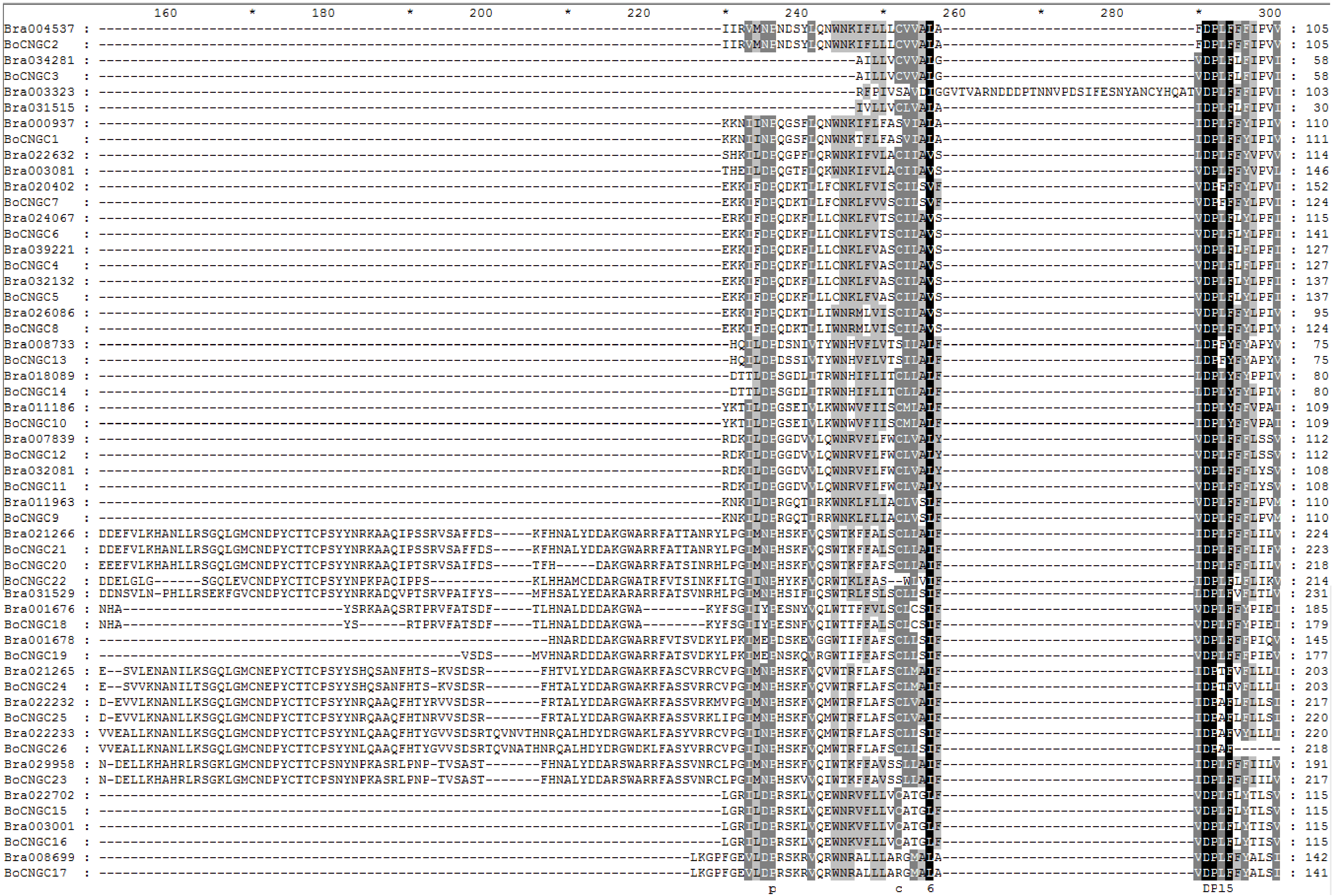
160 * 180 * 200 * 220 * 240 * 260 * 280 * 300
Bra004537 : ---IIRVMNENDSYLQNWNKIFLLLCVVALA---FDPLFFFIPVV : 105
BoCNGC2 : ---IIRVMNENDSYLQNWNKIFLLLCVVALA---FDPLFFFIPVV : 105
Bra034281 : ---AILLVCVVALG---VDPLFLFIPVI : 58
BoCNGC3 : ---AILLVCVVALG---VDPLFLFIPVI : 58
Bra003323 : ---RFPIVSAVDIGGVTVARNDDDPTNNVPDSIFESNYANCYHQATVDPLFFFIPVI : 103
Bra031515 : ---IVLLVCLVALA---VDPLFLFIPVI : 30
Bra000937 : ---KKNIINEQGSFLQNWNKIFLFASVIALA---IDPLFFYIPIV : 110
BoCNGC1 : ---KKNIINEQGSFLQNWNKTFLFASVIALA---IDPLFFYIPIV : 111
Bra022632 : ---SHKILDEQGPFLQRWNKIFVLACIIAVS---LDPLFFYVPVV : 114
Bra003081 : ---THEILDEQGTFLQKWNKIFVLACIIAVS---VDPLFFYVPVI : 146
Bra020402 : ---EKKIFDEQDKTLLFCNKLFVISCIISVF---VDPFFFYLPVI : 152
BoCNGC7 : ---EKKIFDEQDKTLLFCNKLFVVSCIISVF---VDPFFFYLPVI : 124
Bra024067 : ---ERKIFDEQDKFLLLCNKLFVTSCIIAVS---VDPLFLYLPFI : 115
BoCNGC6 : ---EKKIFDEQDKFLLLCNKLFVTSCIIAVS---VDPLFLYLPFI : 141
Bra039221 : ---EKKIFDEQDKFLLLCNKLFVASCIIAVS---VDPLFLFLPFI : 127
BoCNGC4 : ---EKKIFDEQDKFLLLCNKLFVASCIIAVS---VDPLFLFLPFI : 127
Bra032132 : ---EKKIFDEQDKFLLLCNKLFVASCIIAVS---VDPLFLYLPFI : 137
BoCNGC5 : ---EKKIFDEQDKFLLLCNKLFVASCIIAVS---VDPLFLYLPFI : 137
Bra026086 : ---EKKIFDEQDKTLLIWNRMLVISCIIAVS---VDPLFFYLPIV : 95
BoCNGC8 : ---EKKIFDEQDKTLLIWNRMLVISCIIAVS---VDPLFFYLPIV : 124
Bra008733 : ---HQILDEDSNIVTYWNHVFLVTSIIALF---LDPFYFYAPYV : 75
BoCNGC13 : ---HQILDEDSSIVTYWNHVFLVTSIIALF---LDPFYFYAPYV : 75
Bra018089 : ---DTTLDESGDLITRWNHIFLITCLIALF---LDPLYFYPPIV : 80
BoCNGC14 : ---DTTLDESGDLITRWNHIFLITCLIALF---LDPLYFYLPIV : 80
Bra011186 : ---YKTILDEGSEIVLKWNWVFIISCMIALF---IDPLYFFVPAI : 109
BoCNGC10 : ---YKTILDEGSEIVLKWNWVFIISCMIALF---IDPLYFFVPAI : 109
Bra007839 : ---RDKILDEGGDVVLQWNRVFLFWCLVALY---VDPLFFFLSSV : 112
BoCNGC12 : ---RDKILDEGGDVVLQWNRVFLFWCLVALY---VDPLFFFLSSV : 112
Bra032081 : ---RDKILDEGGDVVLQWNRVFLFWCLVALY---VDPLFFFLYSV : 108
BoCNGC11 : ---RDKILDEGGDVVLQWNRVFLFWCLVALY---VDPLFFFLYSV : 108
Bra011963 : ---KNKILDERGQTIRKWNKLFLIACLVSLF---VDPLFFFLPVM : 110
BoCNGC9 : ---KNKILDERGQTIRRWNKLFLIACLVSLF---VDPLFFFLPVM : 110
Bra021266 : DDEFVLKHANLLRSGQLGMCNDPYCTTCPSYYNRKAAQIPSSRVSAFFDS-----KFHNALYDDAKGWARRFATTANRYLPGIMNEHSKFVQSWTKFFALSCLIAIF---IDPLFFFLILV : 224
BoCNGC21 : DDEFVLKHANLLRSGQLGMCNDPYCTTCPSYYNRKAAQIPSSRVSAFFDS-----KFHNALYDDAKGWARRFATTANRYLPGIMNEHSKFVQSWTKFFALSCLIAIF---IDPLFFFLIFV : 223
BoCNGC20 : EEEFVLKHAHLLRSGQLGMCNDPYCTTCPSYYNRKAAQIPTSRVSAIFDS-----TFH-----DAKGWARRFATSINRHLPGIMNEHSKFVQSWTKFFAFSCLIAIF---IDPLFFFLILV : 218
BoCNGC22 : DDELGLG------SGQLEVCNDPYCTTCPSYYNPKPAQIPPS---KLHHAMCDDARGWATRFVTSINKFLTGIINEHYKFVQRWTKLFAS--WIVIF---IDPLFLFLIKV : 214
Bra031529 : DDNSVLN-PHLLRSEKFGVCNDPYCTTCPSYYNRKADQVPTSRVPAIFYS-----MFHSALYEDAKARARRFATSVNRHLPGIMNEHSIFIQSWTRLFSLSCLISIF---LDPLFVFLTLV : 231
Bra001676 : NHA---YSRKAAQSRTPRVFATSDF-----TLHNALDDDAKGWA--------KYFSGIIYEESNYVQLWTTFFVLSCICSIF---VDPLFFYPIEI : 185
BoCNGC18 : NHA---YS------RTPRVFATSDF-----TLHNALDDDAKGWA--------KYFSGIIYEESNFVQIWTTFFALSCICSIF---VDPLFFYPIEI : 179
Bra001678 : ---HNARDDDAKGWARRFVTSVDKYLPKIMEDDSKEVGGWTIFFAFSCLISIF---VDPLFFFPIQV : 145
BoCNGC19 : ---VSDS-----MVHNARDDDAKGWARRFATSVDKYLPKIMEDNSKQVRGWTIFFAFSCLISIF---VDPLFFFPIEV : 177
Bra021265 : E--SVLENANILKSGQLGMCNEPYCTTCPSYYSHQSANFHTS-KVSDSR-------FHTVLYDDARGWAKRFASCVRRCVPGIMNEHSKFVQVWTRFLAFSCLMAIF---IDPTFVFLLLI : 203
BoCNGC24 : E--SVVKNANILTSGQLGMCNEPYCTTCPSYYSHQSANFHTS-KVSDSR-------FHTALYDDARGWAKRFASSVRRCVPGIMNEHSKFVQVWTRFLAFSCLMAIF---IDPTFVFLLLI : 203
Bra022232 : D-EVVLKNANLLKSGQLGMCNDPYCTTCPSYYNRQAAQFHTYRVVSDSR-------FRTALYDDARGWAKRFASSVRKMVPGIMNEHSKFVQMWTRFLAFSCLVAIF---IDPAFLFLLSI : 217
BoCNGC25 : D-EVVLKNANLLKSGQLGMCNDPYCTTCPSYYNRQAAQFHTNRVVSDSR-------FRTALYDDARGWAKRFASSVRKLIPGIMNEHSKFVQMWTRFLAFSCLVAIF---IDPAFLFLLSI : 220
Bra022233 : VVEALLKNANLLKSGQLGMCNDPYCTTCPSYYNLQAAQFHTYGVVSDSRTQVNVTHNRQALHDYDRGWAKLFASYVRRCVPGIINEHSKFVQMWTRFLAFSCLISIF---IDPAFVYLLLI : 220
BoCNGC26 : VVEALLKNANLLKSGQLGMCNDPYCTTCPSYYNLQAAQFHTYGVVSDSRTQVNATHNRQALHDYDRGWDKLFASYVRRCVPGIINEHSKFVQMWTRFLAFSCLISIF---IDPAF------ : 218
Bra029958 : N-DELLKHAHRLRSGKLGMCNDPYCTTCPSNYNPKASRLPNP-TVSAST-------FHNALYDDARSWARRFASSVNRCLPGIMNEHSKFVQIWTKFFAVSSLLAIF---IDPLFFFIILV : 191
BoCNGC23 : N-DELLKHAHRLRSGKLGMCNDPYCTTCPSNYNPKASRLPNP-TVSAST-------FHNALYDDARSWARRFASSVNRCLPGIMNEHSKVVQIWTKFFAVSSLLAIF---IDPLFFFIILV : 217
Bra022702 : ---LGRILDERSKLVQEWNRVFLLVCATGLF---VDPLFLYTLSV : 115
BoCNGC15 : ---LGRILDERSKLVQEWNRVFLLVCATGLF---VDPLFLYTLSV : 115
Bra003001 : ---LGRILDERSKLVQEWNKVFLLVCATGLF---VDPLFLYTISV : 115
BoCNGC16 : ---LGRILDERSKLVQEWNKVFLLVCATGLF---VDPLFLYTISV : 115
Bra008699 : ---LKGPFGEVLDERSKRVQRWNRALLLARGMALA---VDPLFFYALSI : 142
BoCNGC17 : ---LKGPFGEVLDERSKRVQRWNRALLLARGMALA---VDPLFFYALSI : 141
p c 6 DP15

```
                              *        320         *        340         *        360         *        380         *        400         *        420         *        440         *
Bra004537 : DPD---RFCLKLDKKLEAVACVFRTFIDAFYLVHMLFQF----NTGFIAPSS---RGFGRGELVQSSKKIAVRYL--KSYFIIDVLSILPIPQ----------------------------VVVLAVVP-SMSRPASLVTKELIKWAIFC : 214
BoCNGC2   : DPG---RFCLKLDKKLEAVACVFRTFIDAFYVVHMLFQF----NTGFIAPSS---RGFGRGELVQSYKKIAVRYL--KSYFIIDVLSILPIPQ----------------------------VVVLAVVP-SMGRPASLVTKELIKWVIFC : 214
Bra034281 : DSP---NFCFTFDKKLAAVVSAIRTFIDTFYVIHIIFNF----ITEFIAPRS---QVSLRGELIVHSKAIRKRLF--FFQFIVDICSVIPIPQ----------------------------VVVLILIH-RSD---SLVSQAIIKWIIIT : 164
BoCNGC3   : DSP---NFCFTFDKKLAAVVSAIRTFIDTFYVIHIIFNF----ITEFIAPRS---QVSLRGELIVHSKATRKRLF--FFHFIVDICSVIPIPQ----------------------------VVVLILIH-RSD---SLVSQAIIKWIIIT : 164
Bra003323 : DSH---KFCFTLDKKLGVAVCVLRTLIDVFYVIHFIFHF----ITELVAPRS---QASLRG----NSKPIRKRLF--FFYFSVDIVSVLPIPQ----------------------------VMVLTLLS-RKQK-TSLVSKEIIKWAMFC : 207
Bra031515 : DSH---RLCFTYDKKLVATACVFRTLIDTFYGIHIIFHF----ITKHIAPRS---QVSFRGETTVYSVAISERHL--IFYFIVDIVSVLPIP---------------------------------------------------------- : 110
Bra000937 : DGK---KHCINIHSSLEIAASVLRTFVDAFYIIHIVFQF----RTAYVSPLS---RVFGRGELVEDPKAIALKYI--SSYFIIDVLSILPLPQ----------------------------LVVLAVIP-NVDKPVSLLTKDYLITVIFA : 219
BoCNGC1   : DGK---KHCINIHSSLEIAASVLRTFVDAFYIIHIVFQF----RTAYVSPLS---RVFGRGELVEDPKAIALKYL--SSYFIIDVLSILPLPQ----------------------------LVVLAVIP-NVEKPVSLLTKDYLITVIFA : 220
Bra022632 : DDA---KKCIGLDNKMEITASVLRSFTDIFYVIHIIFQF----RTGFIAPSS---RVFGRGVLVEDTRQIAIRYL--SSHFIIDILAVLPLPQVRIEIEIEIDLVLSTCISKPLMIVCFPQVVILIIIP-HMRGSRSLNTKNLIKFIVFF : 251
Bra003081 : DKA---NNCLDVDKKMQTTASVLRSVTDIFYAIHMVFQF----RTGFIAPSS---RVFGRGVLVEDRRKIAKRYL--SSHFIIDILAVLPLP----------------------------QMVISIIIP-RMREPKTLHTKNMIKFIVFF : 255
Bra020402 : DGE---SKCIGIDRKLAITATTFRTFIDVFYLAHMALQL----RTAYIAPSS---RVFGRGELVIDPAQIAKRYL--QRWFIIDFLSVLPVPQ----------------------------IVVWRFLQ-SSRGSDVLATKQALLFIVIV : 261
BoCNGC7   : NGE---SKCIGIDRKLAITATTFRTFIDVFYLAHMALQL----RTAYIAPSS---RVFGRGELVIDPAQIAKRYL--QRWFIIDFLSVLPVPQ----------------------------IVVWRFLQ-RSRGSDVLATKQALLFIVIV : 233
Bra024067 : NDS---GKCIGIDRRLATIATTLRTFIDVFYLFRMALQF----RTAFVAPSS---RVFGRGELVIDPAQIAKRYL--QQYFIVDFLSVLPLPQ----------------------------IVVWRFLY-TSTGGSVLETKQAIRSIIIV : 224
BoCNGC6   : NDS---GKCIGIDRRLATIATTLRTFIDVFYLFRMALQF----RTAFVAPSS---RVFGRGELVIDPAQIAKRYL--QQYFIVDFLSVLPLPQ----------------------------IVVWRFLY-TSTGGSVLETKQAIRSIIIV : 250
Bra039221 : NDK---AKCVGIDRKLAIVTTTLRTVIDSFYLFHMALRF----RTAYVAPSS---RVFGRGELVIDPAQIAKRYL--QQYFIIDLLSVLPVPQ----------------------------IIVWRFLY-SSRGANVLATKQAIRYIVIV : 236
BoCNGC4   : NDK---AKCVGIDRKLAIVTTTLRTVIDSFYLFHMALRF----RTAYVAPSS---RVFGRGELVIDPAQIAKRYL--QQYFIIDLLSVLPVPQ----------------------------IIVWRFLY-SSRGANVLATKQAIRYIVIV : 236
Bra032132 : NDK---AKCIGIDRKLAIVATTLRTVIDSFYLFHMALRF----RTAYVAPSS---RVFGRGELVIDPKQIAKRYL--RQYFIIDLLSVLPLPQ----------------------------ITVWRFLY-TSKGANVLATKQAIRYIVIV : 246
BoCNGC5   : NDK---AKCIGIDRKLAIVATTLRTVIDSFYLFHMALRF----RTAYVAPSS---RVFGRGELVIDPKQIAKRYL--QQYFIIDLLSVLPLPQ----------------------------ITVWRFLY-TSKGANVLATKQAIRYIVIV : 246
Bra026086 : DNS--GSSCIGIDTKLAVTTTTLRTILDVFYLTRMALQF----RTAYIAPSS---RVFGRGELVIDPAKIAQRYL--TRYFIVDFLAVLPLPQ----------------------------IAVWKFLH-GSKGMDVLPTKTALLNIVIT : 205
BoCNGC8   : DNS--GSSCIGIDTKLAVTTTTLRTILDVFYLTRMALQF----RTAYIAPSS---RVFGRGELVIDPAKIAQRYL--TRYFIVDFLAVLPLPQ----------------------------IAVWKFLH-GSKGMDVLPTKTALLNIVIT : 234
Bra008733 : G---G-PACLSVDVGLAATVTFFRSVADLFHLLHIFMKF----RTAFVARSS---RVFGRGELVRDPREIAMKYL--KSDFIVDVAAMLPLPQ----------------------------LVIWLVIP-AATNGTANHANSTIALIVIV : 183
BoCNGC13  : G---G-PACLSVDVGLAATVTFFRSVADLFHLLHIFMKF----RTAFVARSS---RVFGRGELVRDPREIAMKYL--KSDFIVDVAAMLPLPQ----------------------------LVIWLVIP-AATNGTANHANSTIALIVIV : 183
Bra018089 : QA--G-TACMSIDIGFGILVTFFRTLADFSFLIHILLKF----KTAFVSKSS---RVFGRGELVIDRREIAIRYL--KSEFIIDLAATLPLPQ----------------------------IMIWFVIPNAGEFRYAAHQNHTISLVVII : 190
BoCNGC14  : QA--G-TACMSIDIGFGILVTFFRTLADFSFLIHILLKF----KTAFVSKSS---RVFGRGELVMDRREIAIRYL--KSEFIIDLAATLPLPQ----------------------------IMIWFVIPNAGEFRYAAHQNHTISLVVII : 190
Bra011186 : GGNKD-YPCAKTDTNLRILVTFFRTIADLFYLLHIFIKF----RTGFIAPNSST-RVFGRGELVMDPKAIAWRYL--KSDFIIDLVATLPLPQ----------------------------IVIWFVMP-ATRSYRFDHSNNDIALIVIL : 222
BoCNGC10  : GGNKD-YPCAKTDTNLRILVTFFRTIADLFYLLHIFIKF----RTGFIAPNSST-RVFGRGELVMDPKAIAWRYL--KSDFIIDLVATLPLPQ----------------------------IVIWFVMP-ATRSYRFDHSNNDIALIVIL : 222
Bra007839 : KNTGR-SSCMTTDLKLGIVITFFRTLADLFYVLHIVIKF----RTAYVSRTS---RVFGRGELVKDPKLIARRYL--RSDFIVDLIACLPLPQ----------------------------IVSWFILP-SIRSSHSDHTTNAIVLIVIV : 223
BoCNGC12  : KNTGR-SSCMTTDLKLGIVVTFFRTLADLFYVLHIVIKF----RTAYVSRTS---RVFGRGELVKDPKLIARRYL--RSDLIVDLIACLPLPQ----------------------------IVSWFILP-SIRSSHSDHTTNAIVLIVIV : 223
Bra032081 : KRTGR-SSCMTTDLNLGIVVTFFRTLADLFYVLHIVIKF----RTAYVSRTS---RVFGRGELVKDPKLIARRYL--RSDFIVDLIACLPLPQ----------------------------IVSWFILP-SIRSSHSDHTTNAIVLIVIV : 219
BoCNGC11  : KRTGR-SSCMTTDLNLGIVVTFFRTLADLFYVLHIVIKF----RTAYVSRTS---RVFGRGELVKDPKLIARRYL--RSDFIVDLIACLPLPQ----------------------------IVSWFILP-SIRSSHSDHTTNAIVLIVIV : 219
Bra011963 : R---K-EACITIGIRLEVVLTVIRSLADAFYIAQIVIRF----RTAYIAPSS---RVFGRGELVIDSRKIAWRYL--NKSFWIHIVAALPLPQ----------------------------VLIWIVIP-NLRGSPMTNTKNTIRFIIIF : 218
BoCNGC9   : R---K-EACITIGIRLEVVLTVIRSLADAFYIAQIVIRF----RTAYIAPSS---RVFGRGELVIDSRKIAWRYL--NKSFWIHIVAALPLPQ----------------------------VLIWIVIP-NLRGSPMTNTKNTIRFIIIF : 218
Bra021266 : KQNDK---CIVIDWPMAKAFVAVRSVTDILFSVNILIQF----RLAYVAPES---TVVGAGQLVAHPRKIARHYF--RGKFLLDLFIVMPLPQ----------------------------ILILWIIPAHLGASGANYAKNLIRAAVIF : 334
BoCNGC21  : KQNDK---CIMIDWPMAKAFVAVRSVTDILFSVNILIQF----RLAYVAPES---TVVGAGQLVAHPRKIARHYF--RGKFLLDLFIVMPLPQ----------------------------ILILWIIPAHLGASGANYAKNLIRAAVIF : 333
BoCNGC20  : KQNNN---CIEIDWPMAHTFVAVRSVTDVLFSVNILIQF----RLAYVAPES---TVVGAGQLVDHPRKIASHYF--RGKFLLDLFIVMPLPQ----------------------------ILILWIIPAHLAISGANYAKNLIRAAVIF : 328
BoCNGC22  : KENDK---CIMIDWPVAKAFIAVRSVTDVLFFVNIMIQF----RLAYATLES---TVVGVGHIFDHPRKIARNYL--RVKFLLDLFIVMPLPQ----------------------------IWIFWIIPVQLGASGDNYAKNFITAAVIF : 324
Bra031529 : KQNNK---CIVIDWPMAKAFIIVRSVTDALFSVNILIQF----RLAYVSPES---MVVGVTWLVDHPVKIARHYF--QGNFFLDIFIVMPLPQ----------------------------ILISWITPAWLGGSWENNAYSLIQAAVIL : 341
Bra001676 : YKEER---CIKIDWWTTNVFVIVRTITDGLYALNIVIQF----RLAYVDLES---TVAGAGQLVDDPKKIASHYL--RGKFLTDFFIVLPIPQ----------------------------ILILWIIPQLLGTSGANNTKNYIRAAIIV : 295
BoCNGC18  : YKEER---CIKIDWWTTNVFVIVRSITDGLYALNIVIQF----RLAYVDLES---TVVGAGQLVDDPKKIASHYL--RGKFLTDFFIVLPIPQ----------------------------ILILWIIPQLLGTSGANNTKNYIRAAIIV : 289
Bra001678 : SESGK---CIRIDEKMARVLVALRSLTDFLYFVNTLIQC----RLAYTDPKS---TVVGSGQLVKGSVEIAKRYY--RGNFLLDIVIVIPLPQ----------------------------ILILWIIPHVLVIYGANTIKNLICTAVVV : 255
BoCNGC19  : SQSGK---CIRIDEDMAIVLVALRSLTDVLYFVNILIQ---------------------------------------------------------------------------------------------------------------- : 212
Bra021265 : RHDNK---CIEIDWPKTTVLVSLRSMFDLIFFINILIQ----FRMAYVAPES---RIVGAGQLVDHPRKIARNYL--RGKFFLDLFIVLPIPQ----------------------------IMTLSIIPAHLGTSTAGFERNIIRSTFIV : 313
BoCNGC24  : RHDNK---CIEIDWTKTTVLVSLRSMFDLIFFINILIQ----FRMAYVAPES---RIVGAGQLVDHPRKIARNYL--RGKFFLDLFIVLPIPQ----------------------------IMTLSIIPAHLGTSTAGFERNITRSIFIV : 313
Bra022232 : RQDNK---CIEFDWPKTKVFVSLRSMSDLIFFMNILIQ----FRLAYVAPES---RIVGAGQLVDHPRKIASNYL--RGKFLLDLLIVLPMPQ----------------------------IMILSMIPAHLGTPRSELEKNIIRIVFLF : 327
BoCNGC25  : RQDNK---CIEIDWPKTKVFVSLRTMSDLIFFINILIQ----FRLAYVAPES---RIVGAGQLVDHPRKIASKYF--RGKFFLDLLIVVPMPQ----------------------------IMILSMIPAHLGTPRSELEKNIIRIVFLF : 330
Bra022233 : RDDNK---CTDIDWPKATLFLFLRSMSDFIFFINILIQ----FRLAYVAPES---RIVGAGQLVDHPRKIACNYL--RGKFFLDVVIVYPVPQ----------------------------LMILSIIP------KSEFEENAIIGIFLF : 324
BoCNGC26  : --DNK---CTDIDWPKATIFAFLRSMSDFIFFINILIQ----FRLAYVAPES---RIVGAGQLVDHPRKIACNYL--RGKFFLDLLIVYPVPQ----------------------------IMIFSIIP------KSEFEENTVIALFLF : 320
Bra029958 : QKNNK---CIVIDWPIATAFVIVRTLTDVIFFANMLIQ----FRLAYVARES---TVVGAGQLV----------------------------------------------------------LVLSVIPAQLAISGTNYAKNLIRTGIIV : 273
BoCNGC23  : QKNNK---CVVIDWPIATAFVIFRTLTDVIFFANMLIQAPVPFRLAYVARES---TVVGAGQLVDRPKKIALHYF--RGYFIVDLIIVMPFPQ----------------------------VLVLSVIPAQLAISGTNYAKNLIRAGIIV : 331
Bra022702 : N---DACMCLLVDGWLALTVTALRSMTDLLHLWNILIQFKIARWWPYRGGDSDGDINKGDGTRVRM--RGAPPYVKKNG-FFFDIFVILPLPQ----------------------------VVIWVVIPSLLKRGSVTLVVSIILLTFLF : 231
BoCNGC15  : N---DACMCLLVDGWLALTVTALRSVTDLLHLWNILIQFKIARRWPYPSGDSDGDINKGDGTRVRT--RVAPPYVKKNG-FFFDIFVILPLPQ----------------------------VVIWVVIPSLLKRGSVTLVVSIILLTFLF : 231
Bra003001 : N---DACMCLLVDGWLALSITAVRSMTDLLHLWNIWIQFKIARRWPYPGGDSDGDTNKGDETRIRTSRRVAPPYVKKKGTFFFDIFVILPLPQ----------------------------VVIWVVIPSLLKRGSVTLVVSVILVTFLF : 234
BoCNGC16  : N---DACMCLLVDGWLALTITAVRSMTDLLHLWNIWIQFKIARRWPYPGGDSDGDTNKGDETRIRTSRRVAPPYVKKKGTFFFDIFVILPLPQ----------------------------VVIWVVIPSLLKRGSVTLVVSVILVTFLF : 234
Bra008699 : GRT-TGPACLYMDGAFAAVVTVVRTCLDALHLWHVWIQFRLA----YVSRES---LVVGCGKLVWDPRAIASHYARSLTGFWFDVIVILPVPQ----------------------------AVFWLVVPKLIREEKVKLIMTIILLIFLF : 256
BoCNGC17  : GRT-TGPACLYMDGAFAAVVTVVRTCLDALHLWHVWIQFRLA----YVSRES---LVVGCGKLVWDPRAIASHYARSLTGFWFDVIVILPVPQ----------------------------AVFWLVVPKLIREEKVKLIMTIILLIFLF : 255
                    C   d          R3  D                            s      g g l      a  y      f d      p pq
```

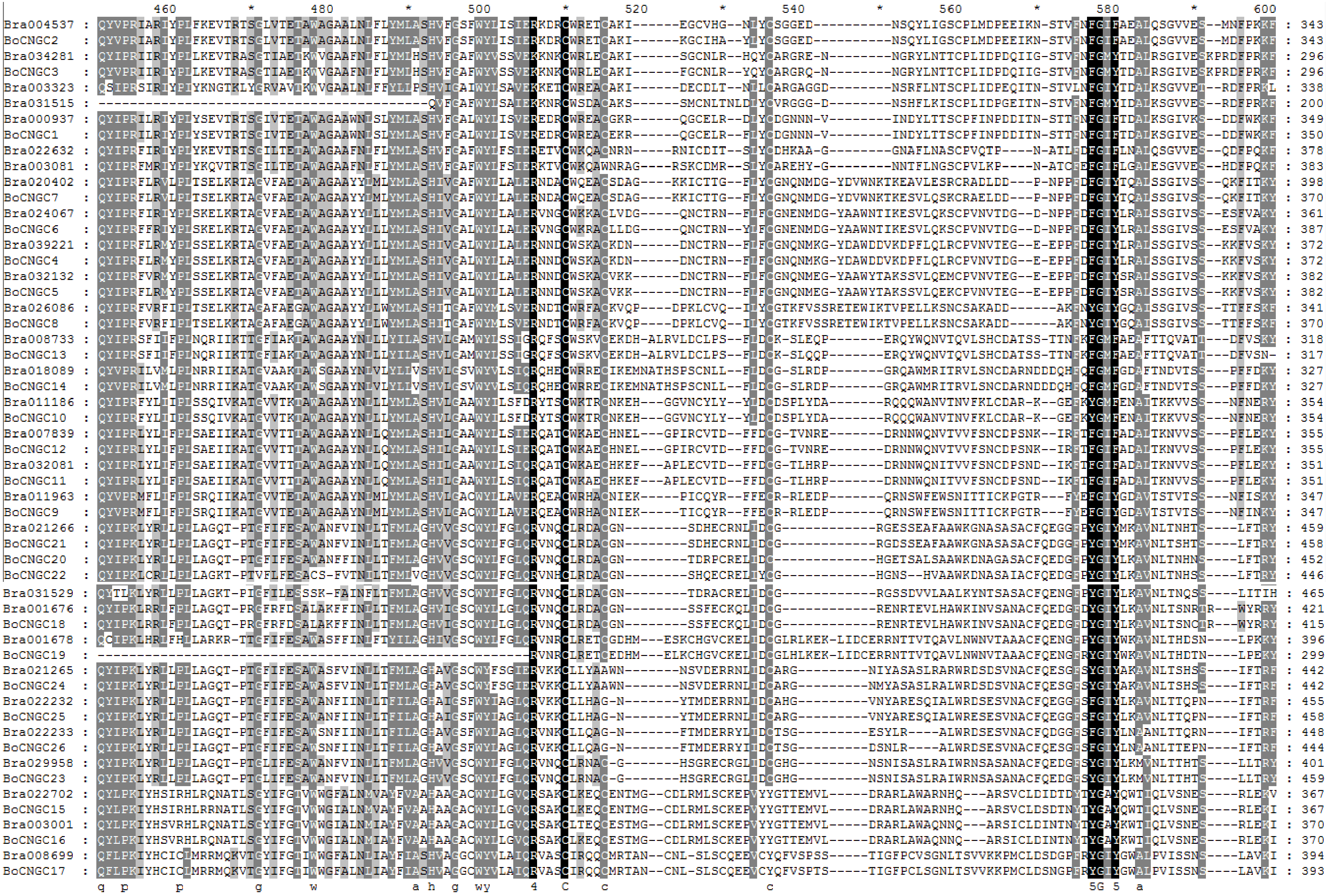

460 480 500 520 540 560 580 600
Bra004537 : 343
BoCNGC2 : 343
Bra034281 : 296
BoCNGC3 : 296
Bra003323 : 338
Bra031515 : 200
Bra000937 : 349
BoCNGC1 : 350
Bra022632 : 378
Bra003081 : 383
Bra020402 : 398
BoCNGC7 : 370
Bra024067 : 361
BoCNGC6 : 387
Bra039221 : 372
BoCNGC4 : 372
Bra032132 : 382
BoCNGC5 : 382
Bra026086 : 341
BoCNGC8 : 370
Bra008733 : 318
BoCNGC13 : 317
Bra018089 : 327
BoCNGC14 : 327
Bra011186 : 354
BoCNGC10 : 354
Bra007839 : 355
BoCNGC12 : 355
Bra032081 : 351
BoCNGC11 : 351
Bra011963 : 347
BoCNGC9 : 347
Bra021266 : 459
BoCNGC21 : 458
BoCNGC20 : 452
BoCNGC22 : 446
Bra031529 : 465
Bra001676 : 421
BoCNGC18 : 415
Bra001678 : 396
BoCNGC19 : 299
Bra021265 : 442
BoCNGC24 : 442
Bra022232 : 455
BoCNGC25 : 458
Bra022233 : 448
BoCNGC26 : 444
Bra029958 : 401
BoCNGC23 : 459
Bra022702 : 367
BoCNGC15 : 367
Bra003001 : 370
BoCNGC16 : 370
Bra008699 : 394
BoCNGC17 : 393

```
                          *        620         *        640         *        660         *        680         *        700         *        720         *        740         *
Bra004537 : FYCFW----------------------------------------WGLRNLSAVGQNLKTSDFEGEIIFAIIICISGLVLFALLIGNMQKYLQSTT-----------------------VRIEEMRVKRRDAEQWMSHRMLPDDLRKRVR : 430
BoCNGC2   : FYCFW----------------------------------------WGLRNLSALGQNLKTSAFEGEIIFAVIICISGLVLFALLIGNMQKYLQSTT-----------------------VRVEEMRVKRRDAEQWMSHRMLPDDLRKRVR : 430
Bra034281 : FYCFW----------------------------------------WGLRNISALGQNLKTSNSVGDIVFALIICVSGLLLFAVLIGNIQKYLQSTT-----------------------IRLDEMEEKKRDTEKWMSNRMLPEYLKERIR : 383
BoCNGC3   : FYCFW----------------------------------------WGLRNISALGQNLKTSNSVGDIVFALIICVSGLLLFAVLIGNIQKYLQSTT-----------------------IRLDEMEEKKRDTEKWMSNRMLPEYLKERIR : 383
Bra003323 : LYCFW----------------------------------------WGLRNISALGQNLKTSNSAGEVFFAIIICVSGVLLFAGLIGNVQKYLQSTS-----------------------IRVDEWEAKKRDTEQWMPSEDLPDDIEKCI- : 424
Bra031515 : FYCFW----------------------------------------WGLRNLSALGQNLETSNSVGEIFFAITICVSGLLLFSVLIGNVQKYLQSTI-----------------------VRINEMEEKRRDTEKWMSYRMLPESLKERIR : 287
Bra000937 : FYCFW----------------------------------------WGLRNLSALGQNLNTSKFVGEIIFAVLICISGLVLFALLIGNMQKYLESTT-----------------------VREEEMRVKRRDAEQWMAHRMLPEDLRKRIR : 436
BoCNGC1   : FYCFW----------------------------------------WGLRNLSALGQNLNTSKFVGEIIFAVLICISGLVLFALLIGNMQKYLESTT-----------------------VREEEMRVKRRDAEQWMAHRMLPEDLRKRIR : 437
Bra022632 : FYCFW----------------------------------------WGLQNLSSLGQNLKTSTYIWEICFAVFISISGLVLFSFLIGNMQTYLQSTT-----------------------TRLEEMRVKRRDAEQWMAHRLLPESLRKRIR : 465
Bra003081 : FYCFW----------------------------------------WGLQNLSSLGQNLKTSTYIWENCFAVFISISGLVLFSFLIGNMQTYLQSTT-----------------------TRLEEMRVKRRDAEQWMSHRLLPDNLRKRIR : 470
Bra020402 : CYCLW----------------------------------------WGLQNLSTLGQGLETSTYPLEIMFSITIAISGLILFALLIGNMQTYLQSLT-----------------------IRLEEMRVKRRDSEQWMHHRMLPQDLRQRVR : 485
BoCNGC7   : CYCLW----------------------------------------WGLQNLSTLGQGLETSTYPLEIMFSITIAISGLILFALLIGNMQTYLQSLT-----------------------IRLEEMRVKRRDSEQWMHHRMLPQDLRQRVR : 457
Bra024067 : FFCLW----------------------------------------WGLQNLSTLGQGLETSTYPGEIIFSIAIAVAGLLLFALLIGNMQTYLQSLT-----------------------IRLEEMRVKRRDSEQWMHHRMLPPDLRERVR : 448
BoCNGC6   : FFCLW----------------------------------------WGLQNLSTLGQGLETSTYPGEIIFSIAIAVAGLLLFALLIGNMQTYLQSLT-----------------------IRLEEMRVKRRDSEQWMHHRMLPPDLRERVR : 474
Bra039221 : FFCLW----------------------------------------WGLQNLSTLGQGLETSTYPGEVIFSIVIAIAGLLLFALLIGNMQTYLQSLT-----------------------IRLEEMRVKRRDSEQWMHHRMLPPELRERVR : 459
BoCNGC4   : FFCLW----------------------------------------WGLQNLSTLGQGLETSTYPGEVIFSIVIAIAGLLLFALLIGNMQTYLQSLT-----------------------IRLEEMRVKRRDSEQWMHHRMLPPELRERVR : 459
Bra032132 : FFCLW----------------------------------------WGLQNLSTLGQGLETSTYPGEVIFSIAIAIAGLLLFALLIGNMQTYLQSLT-----------------------IRLEEMRVKRRDSEQWMHHRMLPPELRERVR : 469
BoCNGC5   : FFCLW----------------------------------------WGLQNLSTLGQGLETSTYPGEVIFSIAIAIAGLLLFALLIGNMQTYLQSLT-----------------------IRLEEMRVKRRDSEQWMHHRMLPPELRERVR : 469
Bra026086 : CYCLW----------------------------------------WGLQNLSTLGQGLQTSTFPGEVLFSIAIAIAGLLLFALLIGNMQTYLQSLT-----------------------VRLEEMRIKRRDSEQWMHHRSLPQNLRERVR : 428
BoCNGC8   : CYCLW----------------------------------------WGLQNLSTLGQGLQTSTFPGEVLFSIAIAIAGLLLFALLIGNMQTYLQSLT-----------------------VRLEEMRIKRRDSEQWMHHRSLPQNLRERVR : 457
Bra008733 : LYCLW----------------------------------------WGLRNLSSYGQNITTSVYLGETLFCITICIFGLILFTLLIGNMQTSLQSMS-----------------------VRVEEWRIKRRDTEEWMRHRQLPPELQERVR : 405
BoCNGC13  : -------------------------------------------------------SYGQNITTSVYLGETLFCITICIFGLILFTLLIGNMQTSLQSMS-----------------------VRVEEWRVKRRDTEEWMRHRQLPPELQERVR : 392
Bra018089 : FYCLW----------------------------------------WGLRNLSSYGQSLAASTLSSETLFSCFICVAGLVFFSHLIGNVQNYLQSTT-----------------------ARLDEWRVRRRDTEEWMRHRQLPQELQERVR : 414
BoCNGC14  : FYCLW----------------------------------------WGLRNLSSYGQSLAASTLSSETLFSCFICVAGLVFFSHLIGNVQNYLQSTT-----------------------ARLDEWRVRRRDTEEWMRHRQLPQELQERVR : 414
Bra011186 : FYCLW----------------------------------------WGLQQLSSYGQNLSTTTFIGETTFAVLIAIFGLVLFAHLIGNMQTYLQSLT-----------------------VRLEEWRLKKRDTEEWMRHRQLPEDLRDRVR : 441
BoCNGC10  : FYCLW----------------------------------------WGLQQLSSYGQNLSTTTFIGETTFAVLIAIFGLVLFAHLIGNMQTYLQSLT-----------------------VRLEEWRLKKRDTEEWMRHRQLPEDLRDRVR : 441
Bra007839 : LYCLW----------------------------------------FGLQQLSSYGQNLDTSTSVLETMFAILVAIFGLVLFALLIGNMQTYLQSIT-----------------------VRLEEWRLKRRDTEEWMGHRQLPQNLRERVR : 442
BoCNGC12  : LYCLW----------------------------------------FGLQQLSSYGQNLDTSTSVLETMFAILVAIFGLVLFALLIGNMQTYLQSIT-----------------------VRLEEWRLKRRDTEEWMGHRQLPQNLRERVR : 442
Bra032081 : LYCLW----------------------------------------FGLQNLSSYGQNLDTSTSVLETMFAILVAIFGLVLFALLIGNMQTYLQSIT-----------------------VRLEEWRLKRRDTEEWMGHRQLPQDLRERVR : 438
BoCNGC11  : LYCLW----------------------------------------FGLQNLSSYGQNLDTSTSVLETMFAILVAIFGLVLFALLIGNMQTYLQSIT-----------------------VRLEEWRLKRRDTEEWMGHRQLPQDLRERVR : 438
Bra011963 : FYCLW----------------------------------------WGLKNLSSLGQNLSTSTYVGEIIFAVVMATLGLVLFALLIGNMQTYLQSTT-----------------------MRLEEWRIRRTDTEQWMHHRQLPPELRQAVR : 434
BoCNGC9   : FYCLW----------------------------------------WGLKNLSSLGQNLSTSTYVGEIIFAVVMATLGLVLFALLIGNMQTYLQSTT-----------------------MRLEEWRIRRTDTEQWMHHRQLPPELRQAVR : 434
Bra021266 : SYSLF----------------------------------------WGFQQISTLAGNQVPSYFLGEVFFTMGIIGLGLLLFALLIGNMQNFLQALG-----------------------RRNLEMTLRRRDVEQWMSHRRLPEGIRKRVR : 546
BoCNGC21  : SYSLF----------------------------------------WGFQQISTLAGNQVPSYFLGEVFFTMGIIGLGLLLFALLIGNMQNFLQALG-----------------------RRNLEMTLRRRDVEQWMSHRRLPEGIRKRVR : 545
BoCNGC20  : SYSLF----------------------------------------WGFQQISTLAGNLIPSYFLGEVFFTMGIIGLGLLLFALLIGNMQNFLQSLG-----------------------RRNLEMTLRRRDVEQWMRHRRLPGDIRKRVR : 539
BoCNGC22  : IYSIL----------------------------------------WGFQQISTLAGNQVPSYFLGEASFTMVIIALGLLLFPLLIGNMQNFLQALG-----------------------GRSLEMS----------HKRFLEGIRGRVR : 522
Bra031529 : KIHILSFLGFSANLIRMFYGFDIKSALVGICGSLTLAYCQLSIVYYSPKQISTLAGNQVPSYFIWEVLFTMGIIGLGLLLFAFLIGNMQNFLQTLG-----------------------QRNLEMTLRQRDAEQWMSHRRFPEGIRKRVR : 592
Bra001676 : SYSLF----------------------------------------WGFQQISTLAGNQVPSYFFGEVLFTMCIIGLGIFLFALLIGNMQNFLQSLG-----------------------RRDTEMTVRRRDVEQWMSHRRFPKDIRKRVR : 508
BoCNGC18  : SYSLF----------------------------------------WGFQQISTLAGNQVPSYFFGEVLFTMCIIGLGIFLFALLIGNMQNFLQSLG-----------------------RRDTEMTVRRRDVEQWMSHRRFPKDIRKRVR : 502
Bra001678 : IYSLF----------------------------------------WGFQQISTLAGNIVPTDFVWEVLFTMAIIGLGIFLFALLIGNMQNFLQSLG-----------------------RRNMEMTLRRRDVNNWMSHRQFPDDIRTRVK : 483
BoCNGC19  : IYSLF----------------------------------------WGFQQISTLGGNQVPSYFIWEVLFTMAIIGLGIFLFALLIGNMQNFLQSLG-----------------------RRNMEMTLRRRDVNKWMSHRQFPEVIRERVR : 386
Bra021265 : SYSLF----------------------------------------WGFQQISTLAGNLSPSYSVGEVFFTMGITGLGLLLFARLIGNMHNFLQAL-----------------------DRRRMEMMLRRRDVEQWMSHRLLPEDIRKRVR : 529
BoCNGC24  : SYSLF----------------------------------------WGFQQISTLAGNLSPSYSVGEVFFTMGITGLGLLLFARLIGNMHNFLQAL-----------------------DRRRMEMMLRRRDVEQWMSHRLLPEDIRKRVR : 529
Bra022232 : SYSLF----------------------------------------WGFQQISTLAGNQTPTYSVGEVYFTMGIIGLGLLLFARLIGNMHNFLQAL-----------------------DRRRMEMMLRRRDVEQWMSHRLLPEDIRKRVR : 542
BoCNGC25  : SYSLF----------------------------------------WGFQQISTLAGNQTPTYSVGEVYFTMGIIGLGLLLFARLIGNMHNFLQAL-----------------------DRRRMEMMLRRRDVEQWMSHRLLPEDIRKRVR : 545
Bra022233 : SFSLF----------------------------------------WGFQQISTLAGNQTPSYSVGEVYFTMGIIGLGLLLFARLIGNMHNFLQAL-----------------------DRRRTEMMLRQRDVEQWMSHRLLPDDIRKRVR : 535
BoCNGC26  : SFSLF----------------------------------------WGFQQISTLAGNQTPSYSLGEVYFTMGIIGLGLLLFARLIGNMHNFLQAL-----------------------DRRRTEMMLRQRDVEQWMSHRLLPDDIRKRVR : 531
Bra029958 : SYSIL----------------------------------------WGFQVVVYSN----------------------------------------------------------NVWSLEMTLRRRDVEQWMSHRRLPEGIRKRVR : 448
BoCNGC23  : SYSIL----------------------------------------WGFQQISTLAGNQVPSYFFPEVVFTMGIIGLGLLLFALLIGNMQNFLQSLGKRMIIFGGWKHKLHWLPSKTGSDRRSLEMTLRRRDVEQWMSHRRLPEGIRKRVR : 569
Bra022702 : LFPIF----------------------------------------WGLMTLSTFGN-LESTTEWSEVVFNIIVLTSGLLIVTMLIGNIKVFLHATT-----------------------SKKQAMHLKMRNIEWWMKKRHLPLGFRQRVR : 453
BoCNGC15  : LFPIF----------------------------------------WGLMTLSTFGN-LESTTEWSEVVFNIIVLTSGLLIVTMLIGNIKVFLHATT-----------------------SKKQAMHLKMRNIEWWMKKRHLPFGLRQRVR : 453
Bra003001 : LFPIF----------------------------------------WGLMTLSTFGN-LESTTEWSEVVFNIIVLTSGLLIVTMLIGNIKVFLHATT-----------------------SKKQAMHLKMRNIEWWMKKRQLPLGYRQRVR : 456
BoCNGC16  : LFPIF----------------------------------------WGLMTLSTFGN-LESTTEWSEVVFNIIVLTSGLLIVTMLIGNIKVFLHATT-----------------------SKKQAMHLKMRNIEWWMKKRQLPLGYRQRVR : 456
Bra008699 : LYPIF----------------------------------------WGLMTLSTFGNDLEPTSNWLEVVFSIVVVLSGLLLFTLLIGNIQVFLHAVM-----------------------AKKRKMQIRCRDMEWWMKRRQLPSRLRQRVR : 481
BoCNGC17  : LYPIF----------------------------------------WGLMTLSTFGNDLEPTSNWLEVVFSIVVVLSGLLLFTLLIGNIQVFLHAVM-----------------------AKKRKMQIRCRDMEWWMKRRQLPSRLRQRVR : 480
                                                   g    s              e  f       g  lf  lign q  l                                e     r  e wm h  lp     r6
```

```
                    760         *        780         *        800         *        820         *        840         *        860         *        880         *        900
Bra004537 : EYEQYKWQETRGVEEEALLSSLPKDIRKEIKRHICLNLLKKVPWFKAMDD-RLLDALCARIKNTVLYTENSYIVREGEPVEDMVFIMRGKLISTTTYGGQTGFFNIAH-LEAGDFCGDLLTWALDP----------NTS-HLPISTRTVQA : 567
BoCNGC2   : KYEQYKWQETRGVEEPVLLSSLPKDIRKDIKRHICLNMLKTVPWFKAMDD-RLLDALCARIKPALYTENSYIVREGEPLEDMVFIMRGKLTSTTTYGGKSGFFNSVS-LGVGQFCGDLLTWALDP----------NTS-HFPISTSTVQA : 567
Bra034281 : RYENYKWRKTRGIEEEALLHSLPKDIRLETKRHIYLTLLNSVPWLNMMDDSWLLEALCDRVKSVFYSANSYIVKEGDPVAEMLIITKGSLKSMIGSSDITGYYDSSY-LQAGDICGDLLFWVLDP----------HSSSSLPISDRSVLT : 522
BoCNGC3   : RYENYKWRKTRGIEEEALLHSLPKDIRLETKRHIYLTLLNSVPWLNMMDDSWLLEALCDRVKSVFYSANSYIVKEGDPVAEMLIITKGSLKSMIGFSDITGYYDSSY-LQAGDICGDLLFWVLDP----------HSSSSLPISDRSVLT : 522
Bra003323 : ------EWEKNSSLERPAHLRSLPKDIRVEAKRNIYLYSLENVPWISFIDDWLLNEIYDRVKPVFYSQKSYIIGEGDPVKEMLIVTYGELESMTESFETSSYSDIQIRLMKGDVWEDLLFWALDP----------HTSPSLPISNGTVTT : 559
Bra031515 : KSEDHKWRETRGTKEESFLRGLPKNIRLEIERQIYQKLLKHMPWFEDMDD-RLLDSVCARIKTVIYTEDSYIVDEGEQIENMLFINRG---DVIVTEKKTTFFRSVG----EFCGEELLSWALLD----------PHSSCVPISSMIIKA : 419
Bra000937 : RYEQYKWQETRGVEENNLLRNLPKDIRRDIKRHFCLDLLKKVPLFEIMDE-QLLDAVCDKIRPVLYTENSYAIREGDPVEEMLFVMRGKLMSATTNGGRTGFFNAVYLKASDFCGEDLLTWALDP----------QSSSHFPISPRTVQA : 575
BoCNGC1   : RYEQYKWQETRGVEENNLLRNLPKDIRRDIKRHFCLDLLKKVPLFEIMDE-QLLDAVCDKIRPVLYTENSYAIREGDPVEEMLFVMRGKLMSATTNGGRTGFFNAVYLNASDFCGEDLLTWALDP----------QSSSHFPISPRTVQA : 576
Bra022632 : RYEQYKWQETRGVDEENLLSNLPKDIRRDIKRHLCLALLMRVPMFEKMDE-QLLDALCDRLQPVLYTEESYIVREGDPVDEMLFIMRGKLLTMTTNGGRTGFFNSEHLGAGDFCGEELLTWALDP----------HTSSNLPISTRTVRA : 604
Bra003081 : RYEQYKWQETRGVNEENLLSNLPKDIRRDIKRHLCLALVMRVPMFEQMDE-QLLDALCDRLQPVLYTEESYIVREGDPVDEMLFIMRGKLLTMTTNGGRTGFFNSEHLGAGDFCGEELLTWALDP----------HTSTNLPISTRTVQA : 609
Bra020402 : RYDQYKWLETRGVDEEYLVQNLPKDIRRDIKRHLCLALVRRVPLFESMDD-KLLDALCMRLKPCLFTESTYLVREGDPVDEMLFIIRGRLESVTTDGGRSGFFNYSLLKEGEFCGEELLTWALDP----------KSGVNLPSSTRTVKA : 624
BoCNGC7   : RYDQYKWLETRGVDEEYLVQNLPKDIRRDIKRHLCLALVRRVPLFESMDE-KLLDALCMRLKPCLFTESTYLVREGDPVDEMLFIIRGRLESVTTDGGRSGFFNYSLLKEGEFCGEELLTWALDP----------KSGVNLPSSTRTVKA : 596
Bra024067 : RYDQYKWLETKGVDEENLVQNLPKDIRRDIKRHLCLALVRRVPLFENMEE-RLLDALCERLKPCLYTEKSYLVREGDPVNEMLFIIRGRLESVTTDGGRSGFFNRSLLKEGDFCGEELLTWALDP----------KSGSNLPSSTRTAKA : 587
BoCNGC6   : RYDQYKWLETKGVDEENLVQNLPKDIRRDIKRHLCLALVRRVPLFENMEE-RLLDALCERLKPCLYTEKSYLVREGDPVNEMLFIIRGRLESVTTDGGRSGFFNRSLLKEGDFCGEELLTWALDP----------KSGSNLPSSTRTAKA : 613
Bra039221 : RYDQYKWLETRGVDEENLVSNLPKDIRRDIKRHLCLALVRRVPLFENMDE-RLLDALCERLKPCLYTEKSFLVREGDPVNEMLFIIRGRLESVTTDGGRSGFYNRSLLKEGDFCGDELLTWALDP----------KSGSNLPSSTRTVKA : 598
BoCNGC4   : RYDQYKWLETRGVDEENLVSNLPKDIRRDIKRHLCLALVRRVPLFENMDE-RLLDALCERLKPCLYTEKSFLVREGDPVNEMLFIIRGRLESVTTDGGRSGFFNRSLLKEGDFCGDELLTWALDP----------KSGSNLPSSTRTVKA : 598
Bra032132 : RYDQYKWLETRGVDEENLVSNLPKDIRRDIKRHLCLALVRRVPLFDNMDE-RLLDALCMRLKPCLYTEKSFLVREGDPVNEMLFIIRGRLECVTTDGGRSGFYKRSLLKEGDFCGDELLTWALDP----------KSGSNLPSSTRTVKA : 608
BoCNGC5   : RYDQYKWLETRGVDEENLVSNLPKDIRRDIKRHLCLALVRRVPLFENMDE-RLLDALCMRLKPCLYTEKSFLVREGDPVNEMLFIIRGRLESVTTDGGRSGFYNRSLLKEGDFCGDELLTWALDP----------KSGSNLPSSTRTVKA : 608
Bra026086 : RYDQYKWLETRGVDEENLVQSLPKDIRRDIKRHLCLNLVRRVPLFANMDE-RLLDALCERLKPSLYTESTYIVREGDPVNEMLFIIRGRLESVTTDGGRSGFFNRGLLKEGDFCGEELLTWALDP----------KAGSNLPSSTRTVKA : 567
BoCNGC8   : RYDQYKWLETRGVDEENLVQSLPKDIRRDIKRHLCLNLVRRVPLFANMDE-RLLDALCERLKPSLYTESTYIVREGDPVNEMLFIIRGRLESVTTDGGRSGFFNRGLLKEGDFCGEELLTWALDP----------KAGSNLPSSTRTVKA : 596
Bra008733 : RFVQYKWLATRGVDEESLLQSLPTDIRREIQRHLCLALVRRVPFFSQMDD-QLLDALCGCLVSSLSTAGTYIFREGDPVDEMLFVIRGQLESSTTNGGRSGFFNSTTLRPGDFCGEELLTWALMP----------NSTLNFPSSTRSVRA : 544
BoCNGC13  : RFVQYKWLATRGVDEESLLQSLPTDIRREIQRHLCLALVRRVPFFSQMDD-QLLDALCGCLVSSLSTAGTYIFREGDPVDEMLFVIRGQLESSTTNGGRSGFFNSTTLRPGDFCGEELLTWALMP----------NSTLNFPSSTRSVRA : 531
Bra018089 : RFVQYKWLTTRGVDEEAILRALPLDIRRQIQRHLCLALVRRVPFFAQMDD-QLLDALCERLVPSLNTKDTYVTREGDPVNEMLFIIRGQMESSTTDGGRSGFFNSITLRPGDFCGEELLTWALMP----------NINQNLPLSTRTVRT : 553
BoCNGC14  : RFVQYKWLTTRGVDEEAILRALPLDIRRQIQRHLCLALVRRVPFFAQMDD-QLLDALCERLVPSLNTKDTYVTREGDPVNEMLFIIRGQMESSTTDGGRSGFFNSITLRPGDFCGEELLTWALMP----------NINQNLPLSTRTVRT : 553
Bra011186 : RYEQYKWLAARGVDEEVLLQSLPTDIRRDIQRHLCLDLVRRVPFFSQMDD-QLLDALCERLVSSLCTEGTYLVREGDLITEMLFIIRGRLESSTTNGGRTGFFNSIILRPGDFCGEELLSWALLP----------KSTLNLPSSTRTVRA : 580
BoCNGC10  : RYEQYKWLAARGVDEEVLLQSLPTDIRRDIQRHLCLDLVRRVPFFSQMDD-QLLDALCERLVSSLCTEGTYLVREGDLITEMLFIIRGRLESSTTNGGRTGFFNSIILRPGDFCGEELLSWALLP----------KSTLNLPSSTRTVRA : 580
Bra007839 : RFVQYKWLATRGVDEETLLHSLPADIRRDIQRHLCLDLVRRVPLFAQMDD-QLLDALCERLVSSLSTQGNYIVREGDPVTEMLFIIRGKLESSTTNGGRTGFFNSITLKPGEFCGEELLAWALLP----------KSKVNLPSSTRTVRA : 581
BoCNGC12  : RFVQYKWLATRGVDEETLLHSLPADIRRDIQRHLCLDLVRRVPLFAQMDD-QLLDALCERLVSSLSTQGNYIVREGDPVTEMLFIIRGKLESSTTNGGRTGFFNSITLKPGEFCGEELLVWALLP----------KSKVNLPSSTRTVRA : 581
Bra032081 : RFVQYKWLATRGVDEETLLHSLPADIRRDIQRHLCLDLVRRVPLFAQMDD-QLLDALCERLVSSLSTQGNYIVREGDPVTEMLFIIRGKLDSSTTNGGRTGFFNSITLKPGEFCGEELLAWALLP----------KSKVNLPSSTRTVRA : 577
BoCNGC11  : RFVQYKWLATRGVDEETLLHSLPADIRRDIQRHLCLDLVRRVPLFAQMDD-QLLDALCERLVSSLSTQGNYIVREGDPVTEMLFIIRGKLDSSTTNGGRTGFFNSITLKPGEFCGEELLAWALLP----------KSKVNLPSSTRTVRA : 577
Bra011963 : KYDQYKWLATRGVDEEAILISLPLDIRRDIKRHLCFDLVRRVPLFDQMDE-RMLDALSERLKPALFTEGTFLVREGDPVNEMLFIIRGHLDSYTTNGGRTGFFNSCLLGPGDFCGEELLTWALDP----------RPVVILPSSTRTVKA : 573
BoCNGC9   : KYDQYKWLATRGVDEEAILISLPLDIRRDIKRHLCFDLVRRVPLFDQMDE-RMLDALSERLKPALCTEGTFLVREGDPVNEMLFIIRGHLDSYTTNGGRTGFFNSCLLGPGDFCGEELLTWALDP----------RPVVILPSSTRTVKA : 573
Bra021266 : EAERFNWAATRGVNEELLFENMPDDLQRDIRRHLFI-FLKKVRIFSLMDE-SILDAIRERLKQRTYISSSTVLHRGGLVEKMVFIVRGEMESIGEDG------SVLPLSEGDVCGEELLTWCLERSSVNPDGTRIRIPSKGLLSYRNVKC : 688
BoCNGC21  : EAERFNWAATRGVNEELLFENMPDDLQRDIRRHLFI-FLKKVRIFSLMDE-SILDAIRERLKQRTYISSSTVLHRGGLVEKMVFIVRGEMESIGEDG------SVLPLSEGDVCGEELLTWCLERSSVNPDGTRIRIPSKGLLSYRNVKC : 687
BoCNGC20  : EAERFNWAATRGVNEELLFKNMPDDLQREIRRHLFI-FLKKVRIFSVMDE-SILDAIRERLKQRTYIRSSMVLHRGGLVEKMVFIVRGEIESVGEDG------YVLPLSEGDVCGEELLTWCLERSSVNPDGTRIRMPSKGLLSNRNVRC : 681
BoCNGC22  : LAEWVNWAATRGFNEKIVFENMPNDLQRDIRRHLFI-FLKKVRVFSKMDE-LILDAIRERLTYRRYISSSTVLHRGGVVEKMVFIVRGEMESFGEDG------YVLPLSEGDVCGEELLAWCLGRSSVNPDGTRIRMPSKGLVSNRNVRC : 664
Bra031529 : EVERFHWAARRGVDEELLFNNMPDDLQRDIRRHLFK-FLNKVRIFTLMDE-SIINAIRERLKHITYISSSVVFSAGDVIEKIVFIVRGEMESIGKDG------SVISLSEGDVFGEELLSWCLERAASNTDGTRIWIKRKGLLSYRSVRC : 734
Bra001676 : EVERLNWNATRGVNEDLLFENMPDDLQRDIRRHLFA-FLKKVRIFSEIDE-SILDAMRARLKQRTYLKNNKVLHRGGVVKKMVFILRGKMESIGEDG------YRTLLSEGDVCGEELLTWCQERSSVNPDGTIIRMPSKGLLSSRDVWC : 650
BoCNGC18  : EVERLNWNATRGVNEELLFENMPDDLQRDIRRHLFA-FLKKVRIFSEIDE-SILDAMRARLKQRTYLKNNKVLHRGGVVKKMVFILRGKMESIGEDG------YRILLSEGDVCGEELLTWCQERSSVNPDGTMIRMPSKGLLSSRDVWC : 644
Bra001678 : NAERLNWSSTRGVNEELLFENMSDDLQRDIRRHLFA-FLKKVRIFSKMDE-FILDAIRERLKHKTYLNGSMVLHSGDLVKKMVFIVRGQMESTREDG------SCTRLSKRDVYGEELLTWY-ERSSLNPDGTRIRMPSKGLLSNRDVKC : 624
BoCNGC19  : DAERLNWSATRGVHEELLFENMPDDLQRDIRRHLFA-FLKKVRIFSKMDE-SILDAIRERLKHKTYVNGSMVLHSGDLVKKMVIIVRGTMESIGEDG------YRTVLSEEFVCGEELLTWCQERSSVNPDGTMIRMPSKGLLSSRAVWC : 528
Bra021265 : EAERFNWAATRGVNEEFLFENMPEDLLRDIRRHLFK-FLKKVRIFSLMDE-SILDSIRERLKQRTYISSSTVLHRRGLVEKMVFIVRGEMESIGQDG------SVLLLSEGDVCGEELLTWCLERSSVNPDGRSIKLPLKGLVSNRSVRC : 671
BoCNGC24  : EAERFNWAATRGVNEELLFENMPEDIRRDIKRHLFK-FLKKVRIFSLMDE-SILDSIRERLKQRTYISSSTVLHRRGLVEKMVFIVRGGMESIGQDG------SVLLLSEGDVFGEELLTWCLERSSVNPDGRSIKLPLKGLVSNRSVRC : 671
Bra022232 : EAERFNWAATRGVNEELLFENMPDDLQRDIKRHLFK-FLKKVRIFSLMDE-SILDAIRERLKQRTYIRSSTVLHRKGLVEKMVFIVRGEMESIGEDG------SVLPLSEGDVCGEELLTWCLERSSVNPDGTRIKMPTKGLVSNRNVRC : 684
BoCNGC25  : EAERFNWAATRGVNEELLFENMPDDLQRDIKRHLFK-FLKKVRIFSLMDE-SILDAIRERLKQRTYIRSSTVLHRKGLVEKMVFIVRGEMESIGEDD------SVLPLSEGDVCGEELLTWCLERSSVNPDGTRIKMPTKGLVSNRNVRC : 687
Bra022233 : EAERFNWAATRGVKEELLFENMPDDLQRDIKRHLFK-FLKKVWIFSLMDE-LILDAIRERLKQRRYIRSSTVLRRKGLVEKMVFIVRGERESIGEDG------SVLPLSEGDVCGEELLTWCLERSSVDPDGTRIKMPTKGLVSNRSVRC : 677
BoCNGC26  : EAERFNWAATRGVKEELLFENMPDDLQRDIKRHLFK-FLKKVWIFSLMDE-LILDAIRERLKQRRYIRSSTVLRRKGLVEKMVFIVRGEMESIGEDG------SVLPLSQGDVCGEELLTWCLERSSVDPDGTRIKMPTKGLVSNRSVRC : 673
Bra029958 : EAERFNWAATRGVNEELLFENMPDDLQGDIRRHLFI-FLKKVRIFSLMDE-SILDSIRERLRQRTYISGSTVLHRWGPVEKIVFVVRGEMESTGEDG------SVLPLSEGDVCGEELLTWCLERSAVNLDG---RMLSKGLLSSRNVKC : 587
BoCNGC23  : EAERFNWAATRGVNEELLFENMPDDLQGDIRRHLFI-FLKKVRIFSLMDE-SILDSIRERLRQRTYISGSTVLHRWGLVEKIVFVVRGEMESIGEDG------SVLPLSEGDVCGEELLTWCLERSAVNLDG---RMLSKGLLSSRNVKC : 708
Bra022702 : NYERQRWAAMRGVDECEMVQNLPEGIRRDIKYHLCLDLVRQVPLFQHMDD-LVLENICDRVKSLIFTKGETIQKEGDAVQRMLFVVRGHLQSSQLLR--DGVKSCCMLGPGNFSGDELLSWCLRR----------PFVERLPPSSSTLVT : 590
BoCNGC15  : NYERQRWAAMRGVDECEMVQNLLEGIRRDIKYHLCLDLVRQVPLFQHMDD-LVLENICDRVKSLIFTKGETIQKEGDAVQRMLFVVRGHLQSSQLLR--DGVKSCCMLGPGNFSGDELLSWCLRR----------PFVERLPPSSSTLVT : 590
Bra003001 : NYERQRWAAMRGVDECEMVQNLPEGIRRDIKYHLCLDLVRQVPLFQHMDD-LVLENICDRVKSLIFTKGETIQKEGDAVQRMLFVVRGHLQSSQLLR--DGVRSCCMLGPGNFSGDELLSWCLRR----------PFVERLPPSTSTLVT : 593
BoCNGC16  : NYERQRWAAMRGVDECEMVQNLPEGIRRDIKYHLCLDLVRQVPLFQHMDD-LVLENICDRVKSLIFTKGETIQKEGDAVQRMLFVVRGHLQSSQLLR--DGVRSCCMLGPGNFSGDELLSWCLRR----------PFVERLPPSTSTLVT : 593
Bra008699 : RFERQRWTALGGEDELEPIQDLPPGIRRDIKRYLCVDLINNVPLFRGMDD-LILDNICDRAKPRVYSKDEKIIREGDPVQRMIFIMRGRVKRNQSLS--KGVVATSTLEPGGYLGDELLSWCLRR----------PFIDRLPPSSATFVC : 618
BoCNGC17  : RFERQRWTALGGEDELEPIHDLPPGIRRDIKRYLCVDLINKVPLFRGMDD-LILDNICDRAKPRVYSKDEKIIREGDPVQRMIFIMRGRVKRNQSLS--KGVVATSTLEPGGYLGDELLSWCLRR----------PFIDRLPPSSATFVC : 617
                  W t g  ee     6p 6  di rh      6  6  f   6d  66 6                        g 6  66f6 G       g               g  g eLL W l                S r
```

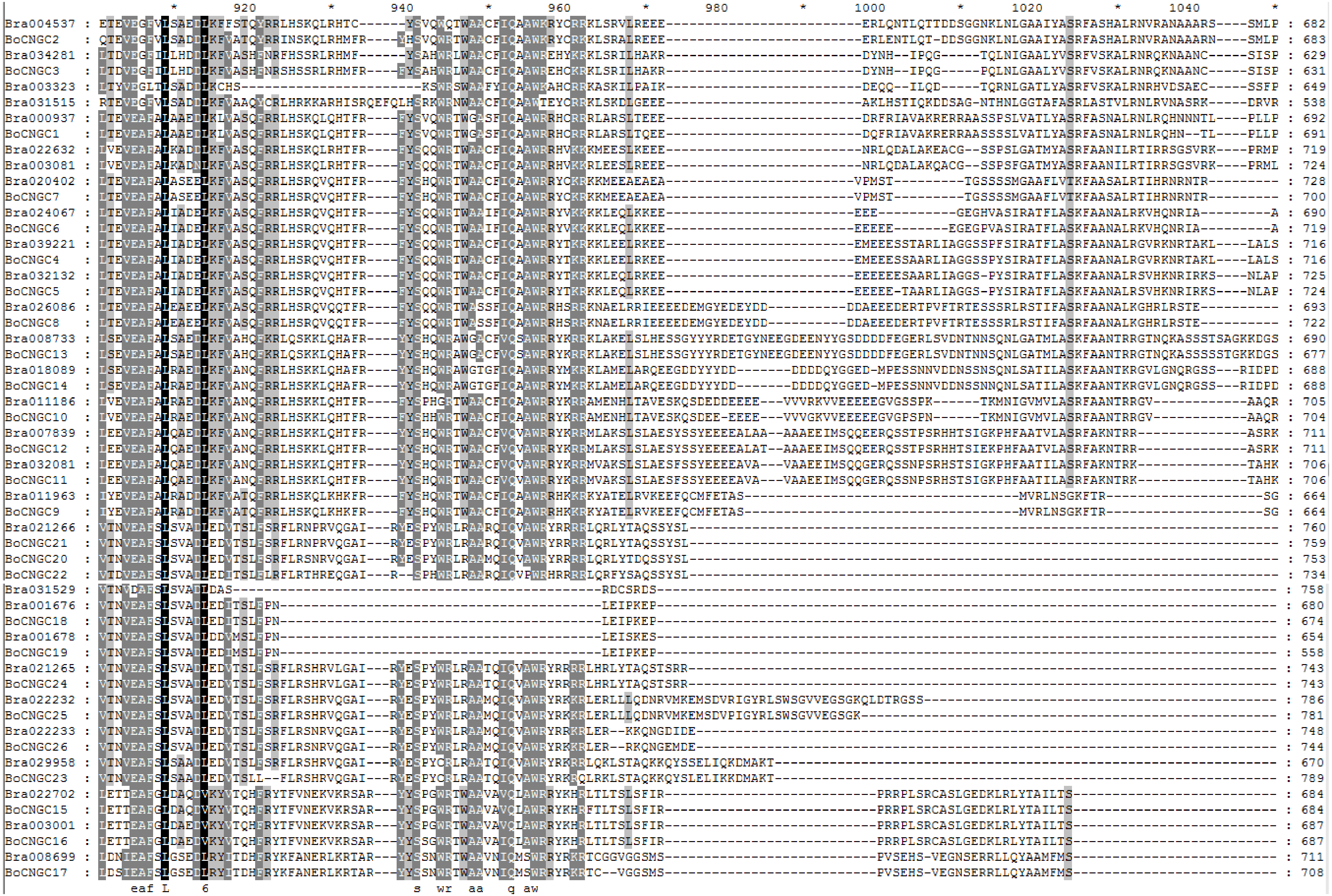
* 920 * 940 * 960 * 980 * 1000 * 1020 * 1040 *
Bra004537 : ETEVEGFVLSAEDLKFFSTQYRRLHSKQLRHTC------YSVQQQTWAACFIQAAWKRYCRRKLSRVLREEE------------------------ERLQNTLQTTDDSGGNKLNLGAAIYASRFASHALRNVRANAAARS----SMLP : 682
BoCNGC2 : QTEVEGFVLSADDLKFVATQYRRINSKQLRHMFR----YHSVQQRTWAACFIQAAWKRYCRKKLSRALREEE------------------------ERLENTLQT-DDSGGNKLNLGAAIYASRFASHALRNVRANAAARN----SMLP : 683
Bra034281 : ITDVEGFILLHDDLKFVASHENRFHSSRLRHMF------YSAHWRLWAACFIQAAWREHYKRKLSRILHAKR------------------------DYNH--IPQG-----TQLNIGAALYVSRFVSKALRNRQKNAANC-----SISP : 629
BoCNGC3 : ITDVEGFILLHDDLKFVASHENRSHSSRLRHMFR----FYSAHWRLWAACFIQAAWREHCKRKLSRILHAKR------------------------DYNH--IPQG-----PQLNLGAALYVSRFVSKALRNRQKNAANC-----SISP : 631
Bra003323 : ITYVEGLTLSADDLKCHS---------------------KSWRSWAAFYIQAAWKAHCRRKASKILPAIK------------------------DEQQ--ILQD-----TQRNLGATLYASRFVSKALRNRHVDSAEC-----SSFP : 649
Bra031515 : RTEVEGFVLSADDLKFVAAQYCRLHRKKARHISRQEFQLHSRKWRNWAACFIQAAWTEYCRRKLSKDLGEEE------------------------AKLHSTIQKDDSAG-NTHNLGGTAFASRLASTVLRNLRVNASRK-----DRVR : 538
Bra000937 : ITEVEAFALAAEDLKLVASQFRRLHSKQLQHTFR----FYSVQQRTWGASFIQAAWRRHCRRRLARSLTEEE------------------------DRFRIAVAKRERRAASSPSLVATLYASRFASNALRNLRQHNNNTL----PLLP : 692
BoCNGC1 : ITEVEAFALAAEDLKLVASQFRRLHSKQLQHTFR----FYSVQQRTWGASFIQAAWRRHCRRRLARSLTQEE------------------------DQFRIAVAKRERRAASSSSLVATLYASRFASNALRNLRQHN--TL----PLLP : 691
Bra022632 : IVEVEAFALKADDLKFVASQFRRLHSKQLRHTFR----FYSQQQRTWAACFIQAAWRRHVKKKMEESLKEEE------------------------NRLQDALAKEACG--SSPSLGATMYASRFAANILRTIRRSGSVRK----PRMP : 719
Bra003081 : IVEVEAFALKADNLKFVASQFRRLHSKQLRHTFR----FYSQQQRTWAACFIQAAWRRHVKKRLEESLREEE------------------------NRLQDALAKQACG--SSPSFGATMYASRFAANILRTIRRSGSVRK----PRML : 724
Bra020402 : ITEVEAFALASEELKFVASQFRRLHSRQVQHTFR----FYSHQQRTWAACFIQAAWRRYCKRKKMEEAEAEA------------------------VPMST---------TGSSSSMGAAFLVTKFAASALRTIHRNRNTR--------- : 728
BoCNGC7 : ITEVEAFALASEELKFVASQFRRLHSRQVQHTFR----FYSHQQRTWAACFIQAAWRRYCKRKKMEEAEAEA------------------------VPMST---------TGSSSSMGAAFLVTKFAASALRTIHRNRNTR--------- : 700
Bra024067 : ITEVEAFALIADELKFVASQFRRLHSRQVQHTFR----FYSQQQRTWAAIFIQAAWRRYVKKKKLEQLKKEE------------------------EEE----------GEGHVASIRATFLASKFAANALRKVHQNRIA---------A : 690
BoCNGC6 : ITEVEAFALIADELKFVASQFRRLHSRQVQHTFR----FYSQQQRTWAAIFIQAAWRRYVKKKKLEQLKKEE------------------------EEEEE---------EGEGPVASIRATFLASKFAANALRKVHQNRIA---------A : 719
Bra039221 : ITEVEAFALIADELKFVASQFRRLHSRQVQHTFR----FYSQQQRTWAACFIQAAWRRYTKRKKLEELRKEE------------------------EMEEESSTARLIAGGSSPFSIRATFLASRFAANALRGVRKNRTAKL----LALS : 716
BoCNGC4 : ITEVEAFALIADELKFVASQFRRLHSRQVQHTFR----FYSQQQRTWAACFIQAAWRRYTKRKKLEELRKEE------------------------EMEEESSAARLIAGGSSPYSIRATFLASRFAANALRGVRKNRTAKL----LALS : 716
Bra032132 : ITEVEAFALIADELKFVASQFRRLHSRQVQHTFR----FYSQQQRTWAACFIQAAWRRYTKRKKLEQLRKEE------------------------EEEEESAARLIAGGS-PYSIRATFLASRFAANALRSVHKNRIRKS----NLAP : 725
BoCNGC5 : ITEVEAFALIADELKFVASQFRRLHSRQVQHTFR----FYSQQQRTWAACFIQAAWRRYTKRKKLEQLRKEE------------------------EEEEE-TAARLIAGGS-PYSIRATFLASRFAANALRSVHKNRIRKS----NLAP : 724
Bra026086 : ITEVEAFALEAEELKFVASQFRRLHSRQVQQTFR----FYSQQQRTWASSFIQAAWRRHSRRKNAELRRIEEEEDEMGYEDEYDD----------DDAEEEDERTPVFTRTESSSRLRSTIFASRFAANALKGHRLRSTE---------- : 693
BoCNGC8 : ITEVEAFALEAEELKFVASQFRRLHSRQVQQTFR----FYSQQQRTWASSFIQAAWRRHSRRKNAELRRIEEEEDEMGYEDEYDD----------DDAEEEDERTPVFTRTESSSRLRSTIFASRFAANALKGHRLRSTE---------- : 722
Bra008733 : ISEVEAFALSAEDLKFVAHQFKRLQSKKLQHAFR----YYSHQQRAWGACFVQSAWRRYKRRKLAKELSLHESSGYYYRDETGYNEEGDEENYYGSDDDDFEGERLSVDNTNNSQNLGATMLASKFAANTRRGTNQKASSSTSAGKKDGS : 690
BoCNGC13 : ISEVEAFALSAEDLKFVAHQFKRLQSKKLQHAFR----YYSHQQRAWGACFVQSAWRRYKRRKLAKELSLHESSGYYYRDETGYNEEGDEENYYGSDDDDFEGERLSVDNTNNSQNLGATMLASKFAANTRRGTNQKASSSSSTGKKDGS : 677
Bra018089 : ISEVEAFALRAEDLKFVANQFRRLHSKKLQHAFR----YYSHQQRAWGTGFIQAAWRRYMKRKLAMELARQEEGDDYYYDD-------DDDDQYGGED-MPESSNNVDDNSSNSQNLSATILASKFAANTKRGVLGNQRGSS---RIDPD : 688
BoCNGC14 : ISEVEAFALRAEDLKFVANQFRRLHSKKLQHAFR----YYSHQQRAWGTGFIQAAWRRYMKRKLAMELARQEEGDDYYYDD-------DDDDQYGGED-MPESSNNVDDNSSNNQNLSATILASKFAANTKRGVLGNQRGSS---RIDPD : 688
Bra011186 : IVEVEAFALRAEDLKFVANQFRRLHSKKLQHTFR----FYSPHGRTWAACFIQAAWRRYKRRAMENHLTAVESKQSDEDDEEE---VVVRKVVEEEEEGVGSSPK------TKMNIGVMVLASRFAANTRRGV------------AAQR : 705
BoCNGC10 : IVEVEAFALRAEDLKFVANQFRRLHSKKLQHTFR----FYSHHWRTWAACFIQAAWRRYKRRAMENHLTAVESKQSDEE-EEEE---VVVGKVVEEEEEGVGPSPN------TKMNIGVMVLASRFAANTRRGV------------AAQR : 704
Bra007839 : IEEVEAFALQAEDLKFVANQFRRLHSKKLQHTFR----YYSHQWRTWAACFVQVAWRRYKRRMLAKSLSLAESYSSYEEEEALAA--AAAEEIMSQQEERQSSTPSRHHTSIGKPHFAATVLASRFAKNTRR--------------ASRK : 711
BoCNGC12 : IEEVEAFALQAEDLKFVANQFRRLHSKKLQHTFR----YYSHQWRTWAACFVQVAWRRYKRRMLAKSLSLAESYSSYEEEEALAT--AAAEEIMSQQEERQSSTPSRHHTSIEKPHFAATVLASRFAKNTRR--------------ASRK : 711
Bra032081 : IEEVEAFALQAEDLKFVANQFRRLHSKKLQHTFR----YYSHQWRTWAACFVQVAWRRYKRRMVAKSLSLAESFSSYEEEEAVA---VAAEEIMSQQGERQSSNPSRHSTSIGKPHFAATILASRFAKNTRK--------------TAHK : 706
BoCNGC11 : IEEVEAFALQAEDLKFVANQFRRLHSKKLQHTFR----YYSHQWRTWAACFVQVAWRRYKRRMVAKSLSLAESFSSYEEEEAVA---VAAEEIMSQQGERQSSNPSRHSTSIGKPHFAATILASRFAKNTRK--------------TAHK : 706
Bra011963 : IYEVEAFALRADDLKFVATQFRRLHSKQLKHKFR----FYSHQWRTWAACFIQAAWRRHKKRKYATELRVKEEFQCMFETAS------------------------------------MVRLNSGKFTR-------------------SG : 664
BoCNGC9 : IYEVEAFALRADDLKFVATQFRRLHSKQLKHKFR----FYSHQWRTWAACFIQAAWRRHKKRKYATELRVKEEFQCMFETAS------------------------------------MVRLNSGKFTR-------------------SG : 664
Bra021266 : VTNVEAFSLSVADLEDVTSLFSRFLRNPRVQGAI---RYESPYWRLRAARQIQVAWRYRRRRLQRLYTAQSSYSL--- : 760
BoCNGC21 : VTNVEAFSLSVADLEDVTSLFSRFLRNPRVQGAI---RYESPYWRLRAARQIQVAWRYRRRRLQRLYTAQSSYSL--- : 759
BoCNGC20 : VTNVEAFSLSVADLEDVTSLFSRFLRSNRVQGAI---RYESPYWRLRAAMQIQVAWRYRRRRLQRLYTDQSSYSL--- : 753
BoCNGC22 : VIDVEAFSLSVADLEDITSLFLRFLRTHREQGAI---R--SPHWRLRAARQIQVPWRHRRRRLQRFYSAQSSYSL--- : 734
Bra031529 : VTNVDAFSLSVADLDAS--RDCSRDS-- : 758
Bra001676 : VTNVEAFSLSVADLEDITSLFPN------------------------------------LEIPKEP-- : 680
BoCNGC18 : VTNVEAFSLSVADLEDITSLFPN------------------------------------LEIPKEP-- : 674
Bra001678 : VTNVEAFSLSVADLDDVMSLFPN------------------------------------LEISKES-- : 654
BoCNGC19 : VTNVEAFSLSVADLEDIMSLFPN------------------------------------LEIPKEP-- : 558
Bra021265 : VTNVEAFSLSVADLEDVTSLFSRFLRSHRVLGAI---RYESPYWRLRAATQIQVAWRYRRRRLHRLYTAQSTSRR--- : 743
BoCNGC24 : VTNVEAFSLSVADLEDVTSLFSRFLRSHRVLGAI---RYESPYWRLRAATQIQVAWRYRRRRLHRLYTAQSTSRR--- : 743
Bra022232 : VTNVEAFSLSVADLEDVTSLFSRFLRSHRVQGAI---RYESPYWRLRAAMQIQVAWRYRKRRLERLLLQDNRVMKEMSDVRIGYRLSWSGVVEGSGKQLDTRGSS-- : 786
BoCNGC25 : VTNVEAFSLSVADLEDVTSLFSRFLRSHRVQGAI---RYESPYWRLRAAMQIQVAWRYRKRRLERLLLQDNRVMKEMSDVPIGYRLSWSGVVEGSGK-- : 781
Bra022233 : VTNVEAFSLSVADLEDVTSLFSRFLRSNRVQGAI---RYESPYWRLRAAMQIQVAWRYRRKRLER--KKQNGDIDE--- : 748
BoCNGC26 : VTNVEAFSLSVADLEDVTSLFSRFLRSNRVQGAI---RYESPYWRLRAAMQIQVAWRYRKRRLER--RKQNGEMDE--- : 744
Bra029958 : VTNVEAFSLSAADLEDVTSLFSRFLRSHRVQGAI---RYESPYCRLRAATQIQVAWRYRKRRLQKLSTAQKKQYSSELIQKDMAKT--- : 670
BoCNGC23 : VTNVEAFSLSAADLEDVTSLL--FLRSHRVQGAI---RYESPYCRLRAATQIQVAWRYRKRRQLRKLSTAQKKQYSLELIKKDMAKT--- : 789
Bra022702 : IETTEAFGLDAQDVKYVTQHFRYTFVNEKVKRSAR---YYSPGWRTWAAVAVQLAWRRYKHRLTLTSLSFIR------------------------PRRPLSRCASLGEDKLRLYTAILTS------------------------------ : 684
BoCNGC15 : IETTEAFGLDAQDVKYVTQHFRYTFVNEKVKRSAR---YYSPGWRTWAAVAVQLAWRRYKHRFTLTSLSFIR------------------------PRRPLSRCASLGEDKLRLYTAILTS------------------------------ : 684
Bra003001 : IETTEAFGLDAEDVKYVTQHFRYTFVNEKVKRSAR---YYSPGWRTWAAVAVQLAWRRYKHRLTLTSLSFIR------------------------PRRPLSRCASLGEDKLRLYTAILTS------------------------------ : 687
BoCNGC16 : IETTEAFGLDAEDVKYVTQHFRYTFVNEKVKRSAR---YYSSGWRTWAAVAIQLAWRRYKHRLTLTSLSFIR------------------------PRRPLSRCASLGEDKLRLYTAILTS------------------------------ : 687
Bra008699 : IDNIEAFSLGSEDLRYITDHFRYKFANERLKRTAR---YYSSNWRTWAAVNIQMSWRRYRKRTCGGVGGSMS------------------------PVSEHS-VEGNSERRLLQYAAMFMS------------------------------ : 711
BoCNGC17 : IDSIEAFSLGSEDLRYITDHFRYKFANERLKRTAR---YYSSNWRTWAAVNIQMSWRRYRKRTC--VGGSMS------------------------PVSEHS-VEGNSERRLLQYAAMFMS------------------------------ : 708
eaf L 6 s wr aa q aw

```
                       1060         *       1080
Bra004537 : HMLSLL--PQKPADPEFPMDET------------ : 702
BoCNGC2   : HMLSLL--PQKPADPEFPMDET------------ : 703
Bra034281 : HMLPPI--PHKPADPEFSKN-------------- : 647
BoCNGC3   : HMLPPI--PHKPADPEFSKN-------------- : 649
Bra003323 : EMLP-----DKPADPEFSKKEA------------ : 666
Bra031515 : QISLP----EKPVDPKFPMDEI------------ : 556
Bra000937 : ---------PKPSEPDFSVDDD------------ : 705
BoCNGC1   : ---------PKPSEPDFGVDDD------------ : 704
Bra022632 : ERM----LLQKPAEPDFNSDDYCI---------- : 739
Bra003081 : ERMPPMLLLQKPAEPDFNSDDYMRIVPIYKNRSL : 758
Bra020402 : --IRELVKLQKPPEPDFTAEDAD----------- : 749
BoCNGC7   : --IRELVKLQKPPEPDFTAEDAD----------- : 721
Bra024067 : KSTKELVIFQKPSEPDFSADDP------------ : 712
BoCNGC6   : KSTKELVIFQKPSEPDFSADDP------------ : 741
Bra039221 : QPTKELLKVQKPPEPDFSADC------------- : 737
BoCNGC4   : QPTKELLKVQKPPEPDFSADC------------- : 737
Bra032132 : PSTKELVKFQKPPEPDFSADC------------- : 746
BoCNGC5   : PSTKELVKFQKPPEPDFSADC------------- : 745
Bra026086 : -SSKRLLNLQKPPEPDFDAE-------------- : 712
BoCNGC8   : -SSKRLLNLQKPPEPDFDAE-------------- : 741
Bra008733 : SNSLKMPQLFKPDEPDFSMDKEDV---------- : 714
BoCNGC13  : SNSLKMPQLFKPDEPDFSMDKEDV---------- : 701
Bra018089 : DPTLKMPKMFKPEDPGFF---------------- : 706
BoCNGC14  : DPTLKMPKMFKPEDPGFF---------------- : 706
Bra011186 : VKDVEMPRFKKPEEPDFSAEPDD----------- : 728
BoCNGC10  : VKDVEMPRFKKPEEPDFSAEPDD----------- : 727
Bra007839 : MKDVDVPMLPKPEEPDFSVDAD------------ : 733
BoCNGC12  : IKDVDVPMLPKPEEPDFSVDAD------------ : 733
Bra032081 : LKDVEVPMLPKPDEPDFSVDGD------------ : 728
BoCNGC11  : LKDVEVPMLPKPDEPDFSVDGD------------ : 728
Bra011963 : SDSGMVSSIQKPVEPDFSSE-------------- : 684
BoCNGC9   : SDSGMVSSIQKPVEPDFSSE-------------- : 684
Bra021266 : ---------------------------------- :   -
BoCNGC21  : ---------------------------------- :   -
BoCNGC20  : ---------------------------------- :   -
BoCNGC22  : ---------------------------------- :   -

Bra031529 : ---------------------------------- :   -
Bra001676 : ---------------------------------- :   -
BoCNGC18  : ---------------------------------- :   -
Bra001678 : ---------------------------------- :   -
BoCNGC19  : ---------------------------------- :   -
Bra021265 : ---------------------------------- :   -
BoCNGC24  : ---------------------------------- :   -
Bra022232 : ---------------------------------- :   -
BoCNGC25  : ---------------------------------- :   -
Bra022233 : ---------------------------------- :   -
BoCNGC26  : ---------------------------------- :   -
Bra029958 : ---------------------------------- :   -
BoCNGC23  : ---------------------------------- :   -
Bra022702 : ---------PKPNPDDFDDY-------------- : 695
BoCNGC15  : ---------PKPNPDDFDDY-------------- : 695
Bra003001 : ---------PKPNPDDFDDY-------------- : 698
BoCNGC16  : ---------PKPNPDDFDDY-------------- : 698
Bra008699 : ---------IRPH-DHLE---------------- : 719
BoCNGC17  : ---------IRPH-DHLE---------------- : 716
```
