# Supplementary material for: Comprehensive genomic analysis of the CNGC gene family in Brassica oleracea: novel insights into synteny, structures, and transcript profiles
Source: BMC Genomics. 2017 Nov 13;18:869. doi: 10.1186/s12864-017-4244-y (PMC5683364; doi:10.1186/s12864-017-4244-y)
Supplement: Supplementary file 21 — Cumulative values of expression for Arabidopsis CNGC genes in different developmental samples. The expression data for 21 days old of wild type and mutant plants was obtained from Schmid et al. [48]. The information about different genotype mutants is given below the figures. (PDF 225 kb) [file 12864_2017_4244_MOESM21_ESM.pdf]

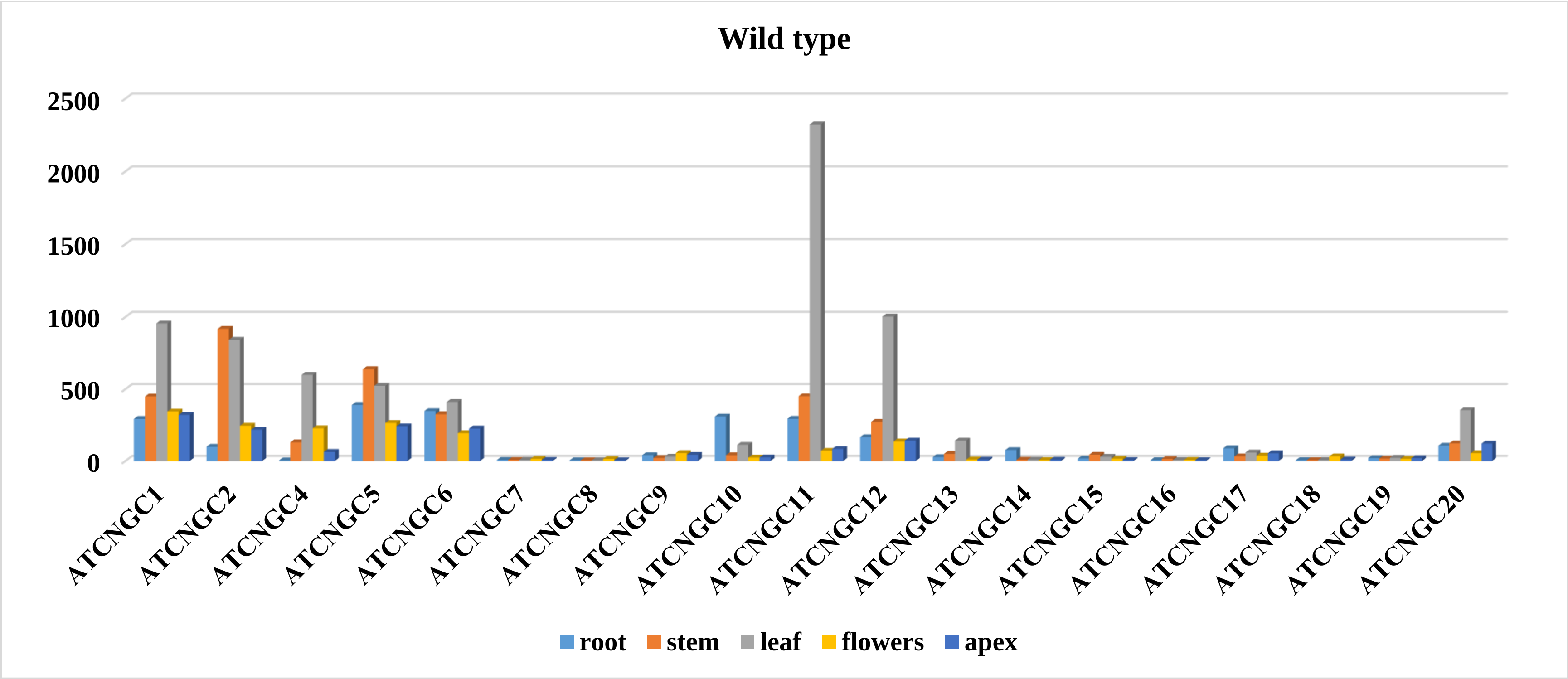

Wild type
2500
2000
1500
1000
500
0
ATCNGC1
ATCNGC2
ATCNGC4
ATCNGC5
ATCNGC6
ATCNGC7
ATCNGC8
ATCNGC9
ATCNGC10
ATCNGC11
ATCNGC12
ATCNGC13
ATCNGC14
ATCNGC15
ATCNGC16
ATCNGC17
ATCNGC18
ATCNGC19
ATCNGC20
root
stem
leaf
flowers
apex

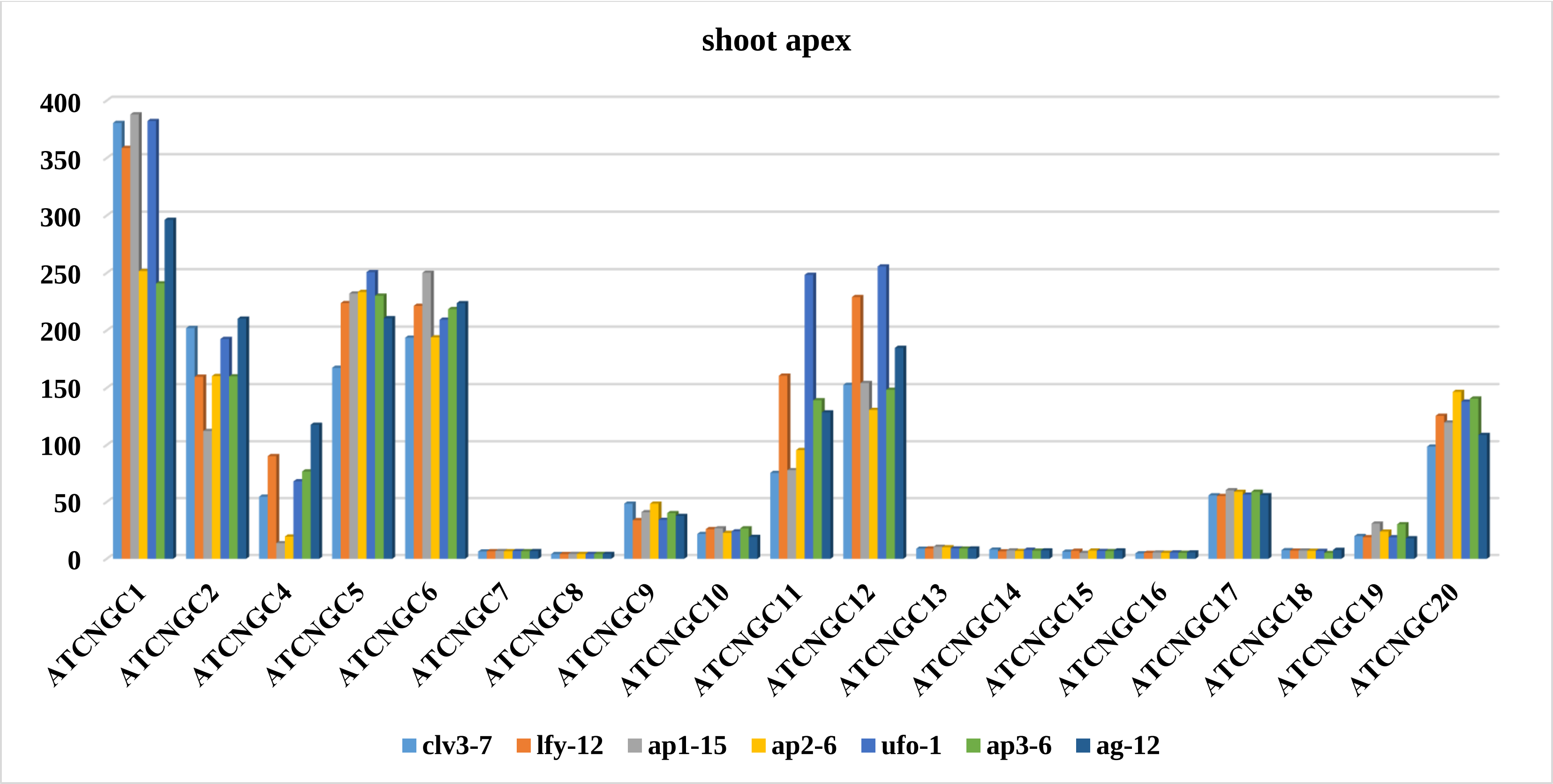

Genotype	Genotype Tissue	Age	Photoperiod	Substrate
clv3-7	shoot apex, inflorescence (after bolting)	21+ days	continuous light	soil
lfy-12	shoot apex, inflorescence (after bolting)	21+ days	continuous light	soil
ap1-15	shoot apex, inflorescence (after bolting)	21+ days	continuous light	soil
ap2-6	shoot apex, inflorescence (after bolting)	21+ days	continuous light	soil
ap3-6	shoot apex, inflorescence (after bolting)	21+ days	continuous light	soil
ag-12	shoot apex, inflorescence (after bolting)	21+ days	continuous light	soil
ufo-1	shoot apex, inflorescence (after bolting)	21+ days	continuous light	soil

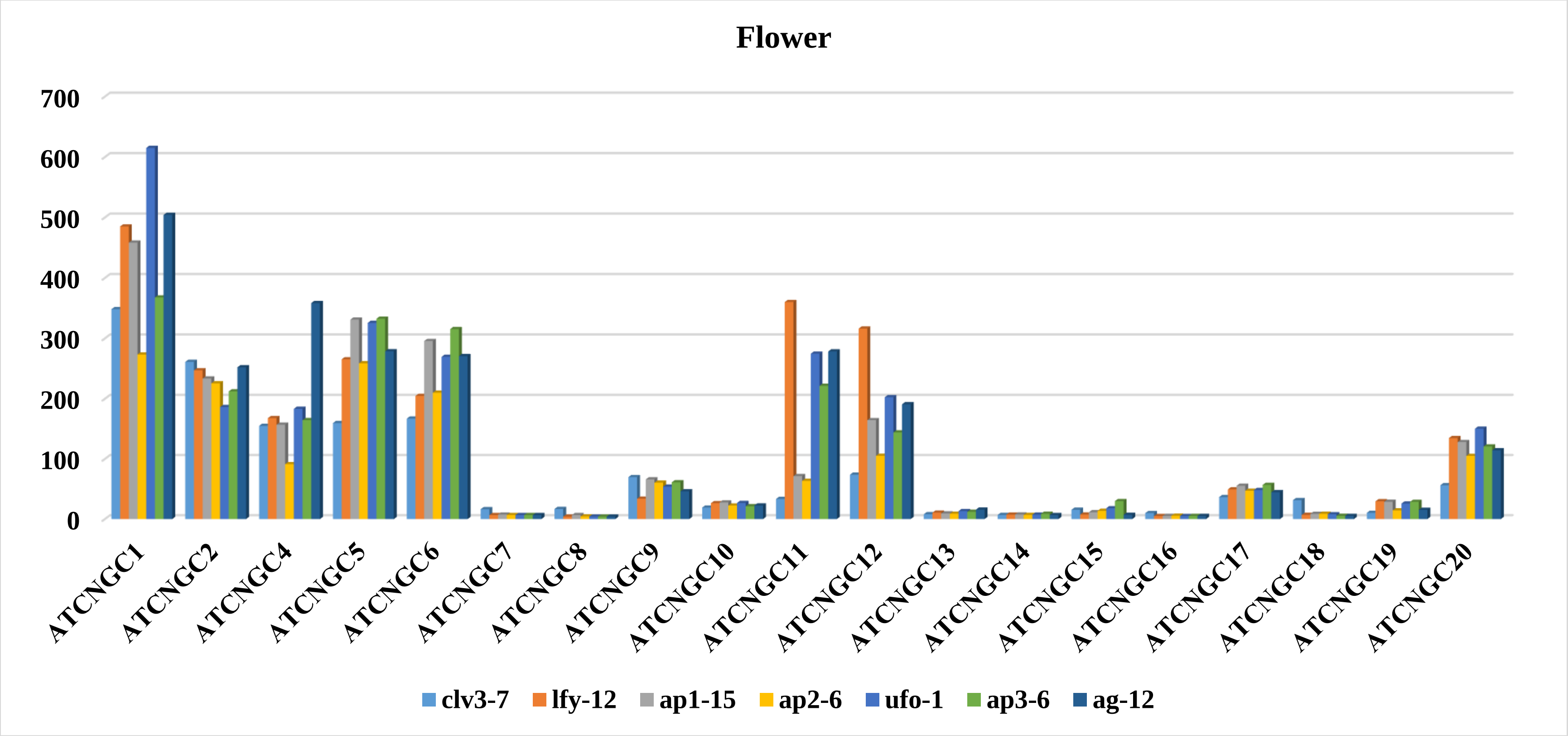

Genotype	Tissue	Age	Photoperiod	Substrate
clv3-7	flower stage 12; multi-carpel gynoeceum; enlarged meristem; increased organ number	21+ days	continuous light	soil
lfy-12	flower stage 12; shoot characteristics; most organs leaf-like	21+ days	continuous light	soil
ap1-15	flower stage 12; sepals replaced by leaf-like organs, petals mostly lacking, 2° flowers	21+ days	continuous light	soil
ap2-6	flower stage 12; no sepals or petals	21+ days	continuous light	soil
ap3-6	flower stage 12; no petals or stamens	21+ days	continuous light	soil
ag-12	flower stage 12; no stamens or carpels	21+ days	continuous light	soil

ufo-1	flower stage 12; filamentous organs in whorls two and three	21+ days	continuous light	soil
