# Supplementary material for: Comprehensive genomic analysis of the CNGC gene family in Brassica oleracea: novel insights into synteny, structures, and transcript profiles
Source: BMC Genomics. 2017 Nov 13;18:869. doi: 10.1186/s12864-017-4244-y (PMC5683364; doi:10.1186/s12864-017-4244-y)
Supplement: Supplementary file 11 — Schematic diagram showing the structures of Arabidopsis CNGC family genes. The exons-introns indicated as red boxes and black lines respectively, and the intron phases are displayed as numbers [0, 1 and 2]. The lengths of each exon and intron can be mapped to the scale given in the bottom. (PDF 154 kb) [file 12864_2017_4244_MOESM11_ESM.pdf]

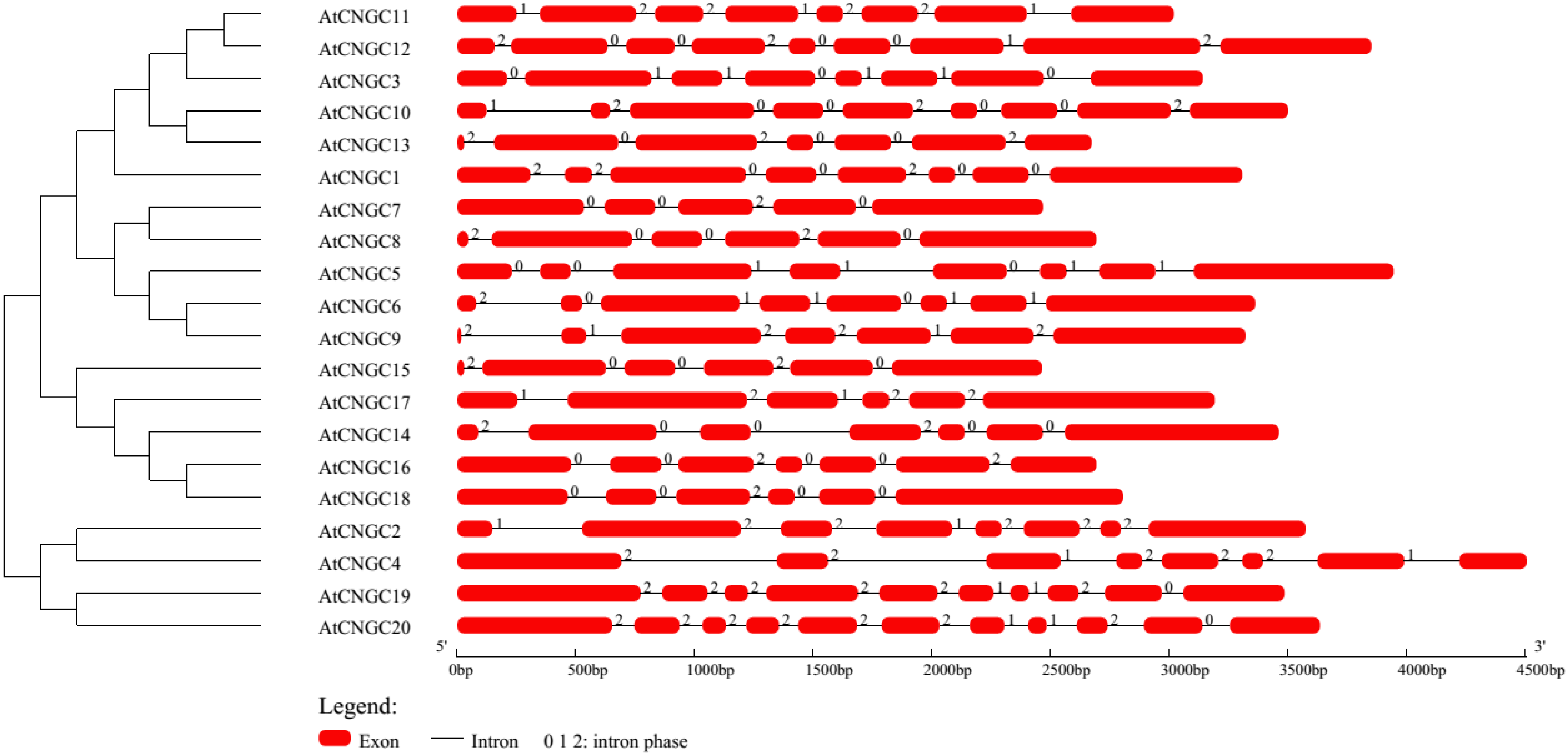
AtCNGC11
AtCNGC12
AtCNGC3
AtCNGC10
AtCNGC13
AtCNGC1
AtCNGC7
AtCNGC8
AtCNGC5
AtCNGC6
AtCNGC9
AtCNGC15
AtCNGC17
AtCNGC14
AtCNGC16
AtCNGC18
AtCNGC2
AtCNGC4
AtCNGC19
AtCNGC20
5'
3'
0bp
500bp
1000bp
1500bp
2000bp
2500bp
3000bp
3500bp
4000bp
4500bp
Legend:
Exon
Intron
0 1 2: intron phase
